# New Metabolites and Bioactive Actinomycins from Marine-Derived *Streptomyces* sp. ZZ338

**DOI:** 10.3390/md14100181

**Published:** 2016-10-11

**Authors:** Xiufang Zhang, Xuewei Ye, Weiyun Chai, Xiao-Yuan Lian, Zhizhen Zhang

**Affiliations:** 1 Ocean College, Zhejiang University, Zhoushan 316021, China; xiufangzhang623@163.com (X.Z.); moshangmowei@163.com (X.Y.); chaiweiyun@163.com (W.C.); 2 College of Pharmaceutical Sciences, Zhejiang University, Hangzhou 310058, China

**Keywords:** marine *Streptomyces* sp. ZZ338, new metabolites, actinomycins, antimicrobial activity, antiproliferative activity, glioma cells, glioma metabolic enzymes

## Abstract

An extract prepared from the culture of a marine-derived actinomycete *Streptomyces* sp. ZZ338 was found to have significant antimicrobial and antiproliferative activities. A chemical investigation of this active extract resulted in the isolation of three known bioactive actinomycins (**1**–**3**) and two new metabolites (**4** and **5**). The structures of the isolated compounds were identified as actinomycins D (**1**), V (**2**), X_0β_ (**3**), 2-acetylamino-3-hydroxyl-4-methyl-benzoic acid methyl ester (**4**), and *N*-1*S*-(4-methylaminophenylmethyl)-2-oxo-propyl acetamide (**5**) based on their nuclear magnetic resonance (NMR) and high resolution electrospray ionization mass spectroscopy (HRESIMS) data as well as their optical rotation. This class of new compound **5** had never before been found from a natural resource. Three known actinomycins showed activities in inhibiting the proliferation of glioma cells and the growth of methicillin-resistant *Staphylococcus aureus*, *Escherichia coli*, and *Candida albicans* and are responsible for the activity of the crude extract. Actinomycin D (**1**) was also found to downregulate several glioma metabolic enzymes of glycolysis, glutaminolysis, and lipogenesis, suggesting that targeting multiple tumor metabolic regulators might be a new anti-glioma mechanism of actinomycin D. This is the first report of such a possible mechanism for the class of actinomycins.

## 1. Introduction

Gliomas are the most aggressive and frequently diagnosed human brain tumors. Despite advances in therapies including chemotherapy, radiotherapy, and surgical resection, the prognosis is still very poor [1]. While chemotherapy has played an important role in the treatment and prevention of cancer, very few drugs have been approved for treating gliomas including temozolomide (TMZ), carmustine, lomustine, and bevacizumab [2]. Furthermore, only TMZ has been independently used for the treatment of gliomas and the efficacy of TMZ remains unsatisfactory [2]. Therefore, there is an urgent need to discover lead compounds for the development of novel anti-glioma drugs.

Many anti-cancer bioactive compounds, originally isolated from marine invertebrates are actually produced by marine microorganisms [3,4]. For example, several anti-cancer metabolites from marine sponges that have progressed to preclinical or clinical trial phases, such as discodermolide, halichondrin B, bryostatin1, and phorboxazole A, may actually be products derived from their microbiotic consortia [5,6,7]. Therefore, bioactive natural products produced by marine microorganisms are promising sources for the discovery and development of novel anti-glioma drugs or drug leads [7,8,9].

During the course of our ongoing project to discover novel antiglioma agents from marine sources [10,11,12,13,14,15], a marine-derived actinomycete strain, ZZ338, was isolated from sea squirts grown on coastal rocks. A crude extract prepared from the culture of this isolated strain ZZ338 in Gause’s liquid medium showed antimicrobial and antiproliferative activities. A chemical investigation of this bioactive extract resulted in the isolation of three known actinomycins (**1**–**3**) and new compound **4**. In order to obtain more bioactive metabolites, a second medium of BMPM was also used to culture this isolated strain ZZ338, which produced actinomycin **2** and new compound **5**. In this study, we report the isolation and culture of strain ZZ338, the structural elucidation of the new metabolites, and the bioactive evaluation of the isolated compounds against the proliferation of glioma cells and the growth of methicillin-resistant *Staphylococcus aureus*, *Escherichia coli*, and *Candida albicans*.

## 2. Results and Discussion

**Figure 1:**
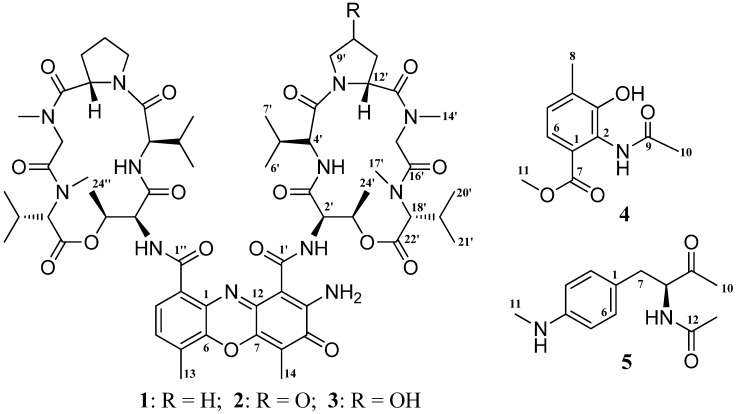
The structures of compounds **1**–**5**.

The isolated strain, ZZ338, was assigned as *Streptomyces* sp. ZZ338 based on its 16S rDNA gene sequence, which completely (99% identity for a 1396 bp stretch of sequence) matched those of several *Streptomyces* strains (Supplementary Materials, Figure S1 and Table S1) in the GenBank database. Two different liquid media of Gause and Bristol Myers Production Medium (BMPM) were used to culture this actinomycete, which produced three known actinomycins (**1**–**3**) and two new metabolites (**4** and **5**) (Figure 1).

The known compounds were proved to be actinomycin D (**1**) [16,17,18], actinomycin V (**2**) [17,19], and actinomycin X_0β_ (**3**) [17,20] based on their nuclear magnetic resonance (NMR) and high resolution electrospray ionization mass spectroscopy (HRESIMS) (Figures S2–S70) data as well as a comparison of published data. The ^13^C and ^1^H NMR data of the three actinomycins are summarized in the Tables S2–S4 (Supplementary Materials).

**Figure 2:**
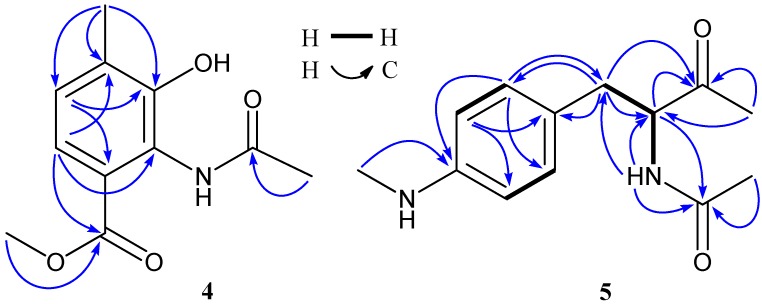
^1^H–^1^H COSY and key HMBC correlations of compounds **4** and **5**.

Compound **4** has a molecular formula of C_11_H_13_NO_4_ deduced from its HRESIMS at *m*/*z* [M − H]^−^ 222.0779 (calcd for C_11_H_12_NO_4_, 222.0766) and ^13^C NMR data. The ^13^C NMR spectrum of **1** exhibited 11 carbons including two carbonyls (δ 170.2, 166.6), six aromatic carbons (δ 149.9, 131.1, 127.4, 124.9, 124.3, 120.7), one methoxy (δ 51.8), and two methyls (δ 23.1, 16.6). In the ^1^H NMR spectrum, two aromatic proton signals appeared as doublets at δ 7.19 (7.9 Hz) and 7.07 (7.9 Hz) and were attributed to H-6 and H-5, three were singlets at δ 3.71, 2.21, 2.07 and easily assigned to one methoxy and two methyls. In addition, a singlet at δ 9.68 indicated the presence of an NH unit. HMBC correlations (Table 1 and Figure 2) of both H-6 (δ 7.19) and H-11 (δ 3.71) with C-7 (δ 166.6) indicated the methoxy at C-7 position. Similarly, HMBC correlations of H-8 (δ 2.21) with C-3 (δ 149.9), C-4 (δ 131.1), and C-5 (δ 127.4) determined the position of a methyl at C-4, while the other methyl related to the acetylamino unit was deduced from a HMBC correlation of H-10 (δ 2.07) with C-9 (δ 170.2). The downfield chemical shift at δ 149.9 (C-3), in combination of the HRESIMS data, suggested a hydroxyl at C-3. Based on the above NMR and MS data analyses, the structure of **4** was elucidated as 2-acetylamino-3-hydroxyl-4-methyl-benzoic acid methyl ester, a new compound. The ^13^C and ^1^H NMR data of **4** are listed in the Table 1. NMR and MS spectra of **4** see Figures S71–S81.

Compound **5** was isolated as a yellow oily and has UV absorbance at 252 and 303 nm. Its HRESIMS showed [M + H]^+^ ion at *m*/*z* 235.1443 and [M + Na]^+^ ion at 257.1263, supporting a molecular formula of C_13_H_18_N_2_O_2_. The ^13^C and ^1^H NMR spectra of **5** showed one ketone (δ_C_ 208.5), one carbonyl (δ_C_ 170.4), six aromatic carbons with four protons (δ_C_ 148.8, 129.9, 129.9, 124.5, 112.1, 112.1; δ_H_ 6.92, 2H, d, 6.43, 2H, d), one nitrogenated methine (δ_C_ 61.0; δ_H_ 4.27), one methylene (δ_C_ 35.0; δ_H_ 2.56, 2.80), and three methyls (δ_C_ 30.2, 27.5, 22.5; δ_H_ 2.61, 2.02, 1.80). The ^1^H NMR spectrum also exhibited a doublet at δ 8.21(1H, d, 6.8 Hz), which was attributed to the NH unit at C-8 position. Two doublets at δ_H_ 6.92 (2H, d, 8.2 Hz) and δ_H_ 6.43 (2H, d, 8.2 Hz) correlating with the carbon signals at δ_C_ 129.9 (CH) and δ_C_ 112.1(CH) observed in the HSQC spectrum, together with the COSY correlation of H-5 (δ 6.43) and H-6 (δ 6.92), indicated that the benzene ring had a symmetrical structure. ^1^H–^1^H COSY correlations (Figure 2) of H-7 (δ 2.56, 2.80) with H-8 (δ 4.27) and H-8 with NH-8 (δ 8.21) confirmed the connection of CH_2_(7)–CH(8)–NH(8). The HMBC correlations (Table 1 and Figure 2) of H-7, H-8, H-10 with C-9 (δ 208.5) and H-11 (δ 2.61) with C-4 (δ 148.8) determined the ketone at C-9 position and the –NHCH_3_ unit at C-4, while the acetylamino group at C-8 position was indicated by HMBC correlations of H-8 and H-13 (δ 1.80) with C-12 (δ 170.4) and NH-8 with C-7 (δ 35.0), C-8 (δ 61.0), and C-12. The planar structure of **5** was thus determined as *N*-1-(4-methylaminophenylmethyl)-2-oxo-propyl acetamide. Compound **5** is closely related to *N*-acetyl-phenylalanine. The (*S*)-*N*-acetyl-phenylalanine and (*R*)-*N*-acetyl-phenylalanine could be assigned based on their optical rotation values, whereby a positive value indicated a *S*-configuration, while a negative value was suggestive of a *R*-configuration [21,22]. Accordingly, the stereochemistry of C-8 in compound **5** was deduced to be *S*-configuration because **5** had a positive optical rotation. Based on the above evidences, compound **5** was identified as *N*-1*S*-(4-methylaminophenylmethyl)-2-oxo-propyl acetamide, a new compound. The ^13^C and ^1^H data of **5** were unambiguously assigned by using HSQC and HMBC correlations (Table 1 and Figure 2). NMR and MS spectra of **5** see Figures S82–S95. To the best of our knowledge, this class of new compound **5** is found from a natural resource for the first time.

All the isolated compounds (**1**–**5**) from this study were evaluated for their activity against the growth of methicillin-resistant *S. aureus*, *E. coli*, and *C. albicans* using the micro broth dilution method. Gentamicin (an antibiotic against both Gram-positive and negative bacteria) and amphotericin B (an antifungal drug) were used as positive control. The results (Table 2) showed that all three actinomycins significantly inhibited the growth of both bacteria and fungi with MIC values of 0.08 to 9.96 μM for actinomycin D (**1**), 0.08 to 9.85 μM for actinomycin V (**2**), and 0.61 to 9.83 μM for actinomycin X_0β_ (**3**). The control gentamicin showed activity with MIC 0.26 to 0.51 μM against both *S. aureus* and *E. coli*, and amphotericin B was active with MIC 0.05 μM for *C. albicans*. However, new compounds **4** and **5** at concentration of 100 μM had no activity against the three tested pathogens.

Compounds **1**–**5** were also tested for their activity in inhibiting the proliferation of human glioma U251 and SHG44 cells and rat glioma C_6_ cells by an SRB assay, a method that measures total cellular protein content. Doxorubicin (DOX) [23] was used as a positive control. Glioma cells were treated with tested compounds for 72 h at different concentrations. The data (Table 3) obtained from this study indicated that three actinomycins had potent activity against the proliferation of the three tested tumor cell lines with IC_50_ values from 1.01 to 10.06 nM for actinomycin D (**1**), 0.42 to 1.80 nM for actinomycin V (**2**), 3.26 to 25.18 nM for actinomycin X_0__β_ (**3**), while the control DOX showed activity with IC_50_ values in a range from 0.70 to 9.61 μM. Unfortunately, new compounds **4** and **5** were inactive.

**Figure 3:**
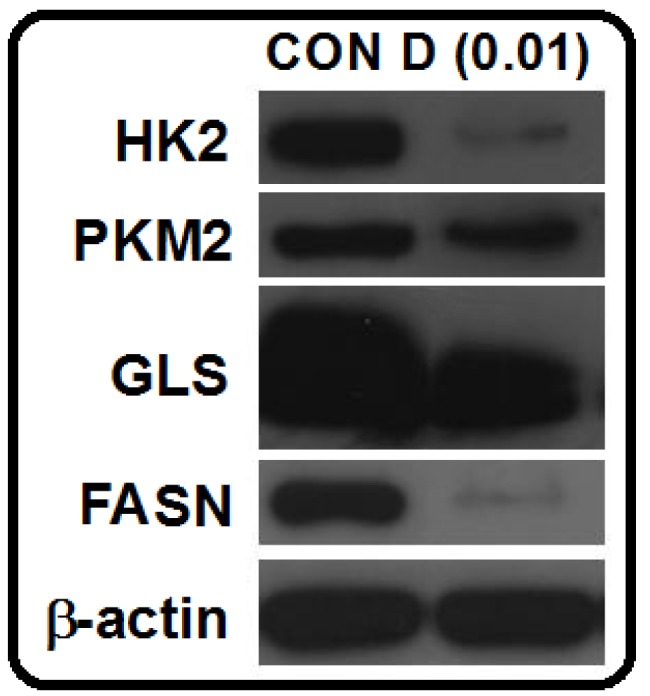
Effects of actinomycin D (**1**) on the expression levels of HK2, PKM2, GLS, and FASN in U87-MG cells. U87-MG cells were treated with actinomycin D (**1**, 0.01 nM) for 48 h. Protein extracted from cells was subjected to a Western blot analysis (HK2: hexokinase 2; PKM2: pyruvate kinase M2; GLS: glutaminase; FASN: fatty acid synthase; β-actin: internal control).

Actinomycin D (**1**) was further investigated for its effects on several important tumor metabolic enzymes from different metabolic pathways including HK2 [24,25] and PKM2 [25,26] from glycolysis, GLS [27,28] from glutaminolysis, and FASN [28,29] from lipogenesis. These metabolic regulators have been demonstrated to be upregulated in the glioma cells and are preferentially used by cancer cells, making them promising targets for the discovery of novel anticancer drugs [24,25,26,27,28,29]. U87-MG cells were treated by actinomycin D (**1**, 0.01 nM) for 48 h. Protein prepared from the actinomycin D-treated U87-MG cells was subjected to a Western blot analysis. The results showed that actinomycin D (**1**) significantly reduced the expression levels of HK2, GLS, and FASN (Figure 3), suggesting that targeting multiple metabolic enzymes may be one of actinomycin D’s anti-glioma mechanisms.

Actinomycins are a family of chromopeptide lactone antibiotics, among which actinomycin D is one of the older anticancer drugs and has been studied extensively and widely used clinically for the treatment of several types of malignant tumors, such as Wilms’ tumor and childhood rhabdomyosarcoma [20]. Despite their initial discovery more than 70 years ago, actinomycins continue to be a focus of many research areas, especially in their biological activity and medicinal use. For example, actinomycin D has been reported to have potent activities against HIV and tuberculosis [20,30,31]. It is known that the antitumor mechanism of actinomycin D is to inhibit transcription by binding DNA at the transcription initiation complex and preventing elongation of the RNA chain by RNA polymerase [32]. In this study, actinomycin D was found to significantly downregulate the expression levels of several glioma metabolic enzymes, suggesting that targeting multiple metabolic regulators might be a new anti-glioma mechanism of actinomycin D.

## 3. Materials and Methods

### 3.1. General Experimental Procedures

NMR spectra were recorded on a Bruker 500 spectrometer (Fällanden, Switzerland) using standard pulse programs and acquisition parameters and chemical shifts were reported in δ (ppm) referencing to the NMR solvent used. Octadecyl-functionalized silica gel (Octadecylsilyl (ODS), Cosmosil 75C_18_-Prep, Nacalai Tesque Inc., Kyoto, Japan) was used for open column chromatography. HPLC separation was performed on an Agilent 1260 HPLC system with a Diode Array Detector (DAD) detector using a Zorbax SB-C_18_ column (250 × 9.4 mm, 5 μm, Agilent Technologies, Palo Alto, CA, USA). All solvents used for this study were purchased from the Sinopharm Chemical Reagent Co. Ltd. (Shanghai, China). Human glioma U251, HSG44, and U87-MG cells and rat glioma C6 cells were purchased from the Cell Bank of the Chinese Academy of Sciences. Methicillin-resistant *S. aureus* ATCC 43300, *E. coli* ATCC 25922, and *C. albicans* were gifts from Zhongjun Ma, Pinmei Wang, and Bin Wu, respectively. Doxorubicin (DOX, >98.0%) was ordered from Sigma-Aldrich, gentamicin (99.6%) and amphotericin B (>95.0%) from Meilune Biotechnology Co. Ltd. (Dalian, China). Nutrient Broth (NB), Mueller-Hinton Broth (MHB), and Gause’s agar were purchased from Hangzhou Microbial Reagent Co. Ltd. (Hangzhou, China). BMPM liquid medium (glucose 20 g, glycerol 20 g, soy flour 10 g, cotton seed embryo meal 10 g, (NH_4_)_2_SO_4_ 1 g, CaCO_3_ 10 g, 1.0 L water) was made in our laboratory.

### 3.2. Isolation and Identification of Strain ZZ338

The strain ZZ338 was isolated from sea squirts collected in January 2016 off the coastal rocks of DongJi Island, close to the East China Sea. The sea squirts were grinded with sterile water in a mortar and then diluted to 1 × 10^−3^ g/mL. The diluted liquid was coated on the surface of Gause’s agar medium. After incubation at 28 °C for 7 days, the ZZ338 colonies were grown and then transferred to a Gause’s agar plate by using a sterile needle. After being cultured at 28 °C for 3 days, the single colony (ZZ338) grew well was transferred onto Gause’s agar slants and stored at 4 °C for later use.

The taxonomic identity of strain ZZ338 was determined by 16S rDNA analysis, which was conducted by Majorbio (Shanghai, China). The top sequence using BLAST (nucleotide sequence comparison) was compared to the GenBank database. A voucher strain of *Streptomyces* sp. ZZ338 was preserved at the Laboratory of Institute of Marine Biology, Ocean College, Zhejiang University, China.

### 3.3. Fermentation of Strain ZZ338

Two different liquid media of Gause and BMPM were applied to culture the strain ZZ338. First, the single colony (ZZ338) was transferred into 250 mL of Gause’s liquid media in a 500 mL Erlenmeyer flask and then incubated at 28 °C for 4 days on a rotary shaker (180 rpm) to prepare the seed broth. Secondly, the seed broth (4 mL) was inoculated into 250 mL of Gause’s liquid media and BMPM’s liquid media in 500 mL Erlenmeyer flasks and then incubated at 28 °C for 8 days on a rotary shaker (180 rpm). A total of 20.0 L of Gause’s fermentation broth and 0.25 L of BMPM’s culture were made for this study.

### 3.4. Isolation of Compounds **1**–**5**

The Gause’s fermentation broth of strain ZZ338 (20.0 L) was filtered to harvest mycelia and filtrate. The mycelia were extracted with MeOH three times to give a MeOH extract and the filtrate was extracted with ethyl acetate three times to afford an EtOAc extract. The two extracts were combined and then fractionated on a column of ODS (600 g) eluting with 40%, 70%, and 100% MeOH to give three fractions of 40 M, 70 M, and 100M. Fraction 100 M was separated by HPLC using a Zorbax SB-C_18_ column (250 × 9.4 mm, 5 μm; mobile phase 90% MeOH; flow rate 1.0 mL/min; detection wavelength: 440 nm) to give compounds **1** (22.1 mg, t_R_ 19.0 min), **2** (96.10 mg, t_R_ 16.9 min), and **3** (5.40 mg, *t*_R_ 15.5 min). Compound **4** (3.60 mg, t_R_ 20.0 min) was obtained from fraction 70 M by HPLC purification (mobile phase: 70% MeOH; detection wavelength: 250 nm).

The BMPM’s fermentation broth (0.25 L) was extracted with MeOH three times. The concentrated MeOH extract was separated by an ODS column eluting with 30%, 50%, 70% and 100% MeOH to give Frs. 30 M, 50 M, 70 M, and 100 M. Compound **5** (26.20 mg, t_R_ 19.0 min) was obtained from fraction 70 M by HPLC purification (mobile phase: 50% MeOH; flow rate: 1.0 mL/min; detection wavelength: 252 nm). Compound **2** was also found in the fraction 100 M by HPLC analysis.

Actinomycin D (**1**): Red amorphous powder; molecular formula C_62_H_86_N_12_O_16_; mp 242–245 °C (lit. 244–246 °C) [18]; UV λ_max_ 240, 445 nm; ^13^C NMR data (125 MHz, in CDCl_3_), see Table S2, ^1^H NMR data (500 MHz, in CDCl_3_) see Table S3; HRESIMS *m*/*z* [M + H]^+^ 1255.6426 (calcd for C_62_H_87_N_12_O_16_, 1255.6363).

Actinomycin V (**2**): Red amorphous powder; molecular formula C_62_H_84_N_12_O_17_; UV λ_max_ 242, 445 nm; ^13^C NMR data (125 MHz, in CDCl_3_), see Table S2, ^1^H NMR data (500 MHz, in CDCl_3_) see Table S3; HRESIMS *m*/*z* [M + H]^+^ 1269.6131 (calcd for C_62_H_85_N_12_O_17_, 1269.6156), [M + Na]^+^ 1291.5945 (calcd for C_62_H_84_N_12_NaO_17_, 1291.5975), [M − H]^−^ 1267.5967 (calcd for C_62_H_83_N_12_O_17_, 1267.5999).

Actinomycin X_0β_ (**3**): Red amorphous powder; molecular formula C_62_H_86_N_12_O_17_; UV λ_max_ 239, 445 nm; ^1^H NMR data (500 MHz, in CDCl_3_) see Table S4; HRESIMS *m*/*z* [M + H]^+^ 1271.6309 (calcd for C_62_H_87_N_12_O_17_, 1271.6312), [M − H]^−^ 1269.6110 (calcd for C_62_H_85_N_12_O_17_, 1269.6156).

Compound **4**: faint yellow amorphous powder; molecular formula C_11_H_13_NO_4_; UV (MeOH) λ_max_ (log ε) 252 (3.21), 304 (2.95) nm; ^13^C NMR data (125 MHz, in DMSO-*d*_6_) and ^1^H NMR data (500 MHz, in DMSO-*d*_6_) see Table 1; HRESIMS *m*/*z* [M − H]^−^ 222.0779 (calcd for C_11_H_12_NO_4_, 222.0766).

Compound **5**: yellow oily; molecular formula C_13_H_18_N_2_O_2_; UV (MeOH) λ_max_ (log ε) 208 (4.41), 252 (4.13), 303 (3.32) nm; ${\left[\alpha \right]}_{D}^{25}$ + 25.6 (*c* 0.10, MeOH); ^13^C NMR data (125 MHz, in DMSO-*d*_6_) and ^1^H NMR data (500 MHz, in DMSO-*d*_6_) see Table 1; HRESIMS *m*/*z* [M + H]^+^ 235.1443 (calcd for C_13_H_19_N_2_O_2_, 235.1447), [M + Na]^+^ 257.1263 (calcd for C_13_H_18_N_2_NaO_2_, 257.1266), [2M + Na]^+^ 491.2626 (calcd for C_13_H_18_N_2_NaO_2_, 491.2634).

### 3.5. Antimicrobial Assay

The micro broth dilution method, as described in the previous study [14], was used to determine the antimicrobial activity of the isolated compounds against the growth of methicillin-resistant *Staphylococcus aureus* ATCC 43300, *Escherichia coli* ATCC 25922, and *Candida albicans*. Gentamicin (an antibiotic against both Gram-positive and Gram-negative bacteria) and amphotericin B (an antifungal drug) were used as positive control.

### 3.6. Tumor Cell Culture

Human glioma U251 and rat glioma C6 cells were cultured in DMEM (Dulbecco’s Modified Eagle Medium, Gibco, Grand Island, NY, USA) with 10% FBS (Fetal Bovine Serum, PAA Laboratories Inc., Toronto, ON, Canada), human glioma SHG44 in RPMI-1640 (Roswell Park Memorial Institution 1640, Gibco, Grand Island, NY, USA), and human glioma U87-MG cells in MEM (Minimum Essential Medium, Gibco, Grand Island, NY, USA). All cells were incubated at 37 °C in a humidified incubator with 5% CO_2_. Cells were used for experiment after the third generation.

### 3.7. Sulforhodamine B (SRB) Assay

The previously described SRB assay [10,11,15] was applied to evaluate the activity of isolated compounds to inhibit the proliferation of different glioma cell lines. Doxorubicin was used as a positive control. Briefly, glioma cells were treated with different concentrations of tested compounds after cells adhesion for 24 h. After 72 h of the treatment, compound-treated cells were fixed with 50 μL of 10% cold TCA solution at 4 °C for 1 h, washed with distilled water five times, and then dried at room temperature. The dried cells were stained with 50 μL of 0.4% SRB for ten minutes and rinsed with 1% acetic acid solution five times. After being dried, dye was dissolved in 10 mM Tris buffer and measured at 515 nm on a microplate reader (Bio-Tech, Winooski, VT, USA).

### 3.8. Western Blot Analysis

A Western blot was used to determine the expression levels of tumor metabolic regulators. The detailed procedures, including protein sample preparation, determination of protein concentration, and a Western blot analysis was referred to a previous publication [12].

## 4. Conclusions

The bioactive metabolite-produced *Streptomyces* sp. strain, ZZ338, was isolated from sea squirts. This isolated actinomycete, cultured in two different media, produced known actinomycins D (**1**), V (**2**), X_0β_ (**3**) and new compounds of 2-acetylamino-3-hydroxyl-4-methyl-benzoic acid methyl ester (**4**) and *N*-1*S*-(4-methylaminophenylmethyl)-2-oxo-propyl acetamide (**5**). The three actinomycins significantly inhibited the growth of both bacteria and fungi and the proliferation of different glioma cell lines; and are the components responsible for the activity of the crude extract of strain ZZ338. Interestingly, actinomycins D (**1**) was found to downregulate several glioma metabolic enzymes of different metabolic pathways (glycolysis, glutaminolysis, and lipogenesis), suggesting that targeting multiple glioma metabolic regulators might be a new antitumor mechanism of actinomycin D. To the best of our knowledge, such a possible mechanism for this class of actinomycins is the first report herein. Additionally, for the first time, the class of new compound 5 is found from natural resources.

## Figures and Tables

**Table 1:** ^13^C and ^1^H NMR data of compounds **4** and **5**.

No.	4			5		
	^13^C, Type	^1^H (*J* = Hz)	HMBC (H → C)	^13^C, Type	^1^H (*J* = Hz)	HMBC (H → C)
1	124.3, C	–	–	124.5, C	–	–
2	124.9, C	–	–	129.9, CH	6.92, d (8.2)	C-4, 6, 7
3	149.9, C	–	–	112.1, CH	6.43, d (8.2)	C-1, 4, 5
4	131.1, C	–	–	148.8, C	–	–
5	127.4, CH	7.07, d (7.9)	C-1, 3	112.1, CH	6.43, d (8.2)	C-1, 3, 4
6	120.7, CH	7.19, d (7.9)	C-2, 4, 7	129.9, CH	6.92, d (8.2)	C-2, 4, 7
7	166.6, C	–	–	35.0, CH_2_	2.56, dd (14.0, 9.0); 2.80, dd (14.0, 5.4)	C-1, 2, 6, 8, 9
8	16.6, CH_3_	2.21, s	C-3, 4, 5	61.0, CH	4.27, m	C-1, 7, 9, 12
9	170.2, C	–	–	208.5, C	–	–
10	23.1, CH_3_	2.07, s	C-9	27.5, CH_3_	2.02, s	C-8, 9
11	51.8, CH_3_	3.71, s	C-7	30.2, CH_3_	2.61, s	C-4
12				170.4, C	–	–
13				22.5, CH_3_	1.80, s	C-12
NH		9.68, s			8.21, d (6.8)	C-7, 8, 12

**Table 2:** Antimicrobial activity of actinomycins D (**1**), V (**2**), and X_0β_ (**3**) (MIC: μM).

Microbes	Actinomycin D (1)	Actinomycin V (2)	Actinomycin X_0__β_ (3)	Gentamicin	Amphotericin
*S. aureus*	0.08	0.08	0.61	0.26	-
*E. coli*	0.12	0.12	0.61	0.51	-
*C. albicans*	9.96	9.85	9.83	-	0.05

**Table 3:** Antiproliferative activity of actinomycins D (**1**), V (**2**), and X_0__β_ (**3**) against glioma cells (IC_50_).

Glioma Cells	Actinomycin D (1, nM)	Actinomycin A_5_ (2, nM)	Actinomycin X_0__β_ (3, nM)	DOX (μM)
U251	10.06 ± 0.68	1.80 ± 0.19	8.71 ± 0.66	9.61 ± 1.25
SHG44	3.31 ± 0.25	1.37 ± 0.07	3.26 ± 0.32	2.54 ± 0.23
C_6_	1.01 ± 0.05	0.42 ± 0.23	25.18 ± 0.47	0.70 ± 0.01

## Supplementary Materials

The following are available online at www.mdpi.com/1660-3397/14/10/181/s1, NMR and HRESIMS spectra (Figures S1–S95) of compounds **1**–**5** as well as other supporting data.
