# Supplementary material for: New Metabolites and Bioactive Actinomycins from Marine-Derived Streptomyces sp. ZZ338
Source: Mar Drugs. 2016 Oct 11;14(10):181. doi: 10.3390/md14100181 (PMC5082329; doi:10.3390/md14100181)
Supplement: Supplementary file 1 [file marinedrugs-14-00181-s001.pdf]

Supplementary Materials: New Metabolites and Bioactive Actinomycins from Marine-derived *Streptomyces* sp. ZZ338

Xiufang Zhang, Weiyun Chai, Xuewei Ye, Xiao-Yuan Lian and Zhizhen Zhang

CONTENT

Table S1. Sequences producing significant alignments……………………………….….…….. 2

Table S2. ^{13}C NMR data of actinomycins D (**1**) and V (**2**) (in $CDCl_3$-*d*)…..….….……….....…. 2

Table S3. ^{1}H NMR data of actinomycins D (**1**) and V (**2**) (in $CDCl_3$-*d*)…..……….……….…….. 3

Table S4. Main ^{1}H NMR data of actinomycin X0_{β} (**3**), in $CDCl_3$-*d*)……………………..…..…. 3

Figure S1. 16S rDNA sequence of *Streptomyces* sp. ZZ338………………….…………....…...…. 4

Figures S2–S7. ^{1}H NMR spectra of actinomycin D (**1**)……………….…………….…............. 4–7

Figures S8-S14. ^{13}C NMR spectra of actinomycin D (**1**)……………...........……….…...…... 7–10

Figures S15–S20. DEPT spectra of actinomycin D (**1**)…………………………...….............. 11–13

Figures S21–S24. ^{1}H–^{1}H COSY spectra of actinomycin D (**1**)……….............…...……..…... 14–15

Figures S25–S28. HSQC spectra of actinomycin D (**1**)………………..........…………...….. 16–17

Figures S29–S34. HMBC spectra of actinomycin D (**1**)………………………..…........…..... 18–20

Figure S35. HRESIMS of actinomycin D (**1**)……...……............………….…...……... 21

Figures S36–S40. ^{1}H NMR spectra of actinomycin V (**2**)………...…………….…..…............. 21–23

Figures S41–S48. ^{13}C NMR spectra of actinomycin V (**2**)…………....……….....….............. 24–27

Figures S49–S53. DEPT spectra of actinomycin V (**2**)……………………….……...…............ 28–30

Figures S54–S57. ^{1}H-^{1}H COSY spectra of actinomycin V (**2**)……………….………..…............ 30–32

Figures S58–S63. HSQC spectra of actinomycin V (**2**)…………………………............….... 32–35

Figure S64. HRESIMS of actinomycin V (**2**)……………………...………..............…...….... 35

Figures S65–S69. ^{1}H NMR spectra of actinomycin X0_{β} (**3**)………....……………...…............... 36–38

Figure S70. HRESIMS of actinomycin X0_{β} (**3**)………....……………..……………..................... 38

Figures S71–S72. ^{1}H spectra of compound **4**……………………………………………............... 39

Figures S73–S75. ^{13}C spectra of compound **4**………………………………...………................. 40–41

Figure S76. HSQC spectrum of compound **4**……………………………...……….................. 41

Figures S77–S80. HMBC spectra of compound **4**……………………...…...……............... 42–43

Figure S81. HRESIMS of compound **4**……………….…………………...…....…….................. 44

Figures S82–S84. ^{1}H spectra of compound **5**………………………………………….............. 44–45

Figures S85–S87. ^{13}C spectra of compound **5**…………………………………….................. 46–47

Figures S88–S90. ^{1}H-^{1}H COSY spectra of compound **5**……………………................... 47–48

Figures S91–S94. HMBC spectra of compound **5**………………………...….................. 49–50

Figure S95. HRESIMS of compound **5**……………………………………....…................... 51

Table S1. Sequences producing significant alignments.

Accession	Description	Max Score	Total Score	Query Coverage	Evalue	Ident
NC_016114.1	*Streptomyces pratensis* ATCC 33331, complete genome	2558	25317	100%	0.0	99%
NC_010572.1	*Streptomyces griseus subsp. griseus* NBRC 13350 DNA, complete genome	2558	25352	100%	0.0	99%
NZ_CP013738.1	*Streptomyces globisporus* C-1027, complete genome	2553	15319	100%	0.0	99%
NZ_JOAZ01000047.1	*Streptomyces halstedii* strain NRRL ISP-5068 contig47.1, whole genome shotgun sequence	2547	2547	100%	0.0	99%
NZ_JQJU01000080.1	*Streptomyces atratus* strain OK008 EW57DRAFT_scaffold00076.76_ C, whole genome shotgun sequence	2519	2519	100%	0.0	99%
NZ_LGDD01000116.1	*Streptomyces* sp. WM6378 P402contig199.1, whole genome shotgun sequence	2483	2483	100%	0.0	99%

Table S2. ^{13}C NMR data of actinomycins D (**1**) and V (**2**) (in $CDCl_3$-*d*).

No.	1	2	No.	1	2
1	129.1, C	129.2, C	2	132.4, C	132.1, C
3	125.7, CH	126.1, CH	4	130.4, CH	130.5, CH
5	127.8, C	128.0, C	6	140.5, C	140.6, C
7	145.1, C	145.1, C	8	113.5, C	113.6, C
9	179.1, C	179.1, C	10	147.8, C	147.6, C
11	101.6, C	101.7, C	12	145.9, C	146.0, C
13	15.1, CH3	15.1, CH3	14	7.7, CH3	7.7, CH3
1′	166.6 ^{a}, C	166.4, C	1″	166.6 ^{a}, C	166.4, C
2′	55.2, CH	54.8, CH	2″	54.9, CH	55.1, CH
3′	168.6, C	168.9, C	3″	169.1, C	169.1, C
4′	58.9, CH	58.6, CH	4″	58.7, CH	57.2, CH
5′	31.5, CH	31.7, CH	5″	31.8, CH	31.9, CH
6′	19.0 ^{b}, CH3	18.9 ^{a} CH3	6″	19.0 ^{b}, CH3	19.0 ^{a}, CH3
7′	19.3 ^{b}, CH3	19.2 ^{a}, CH3	7″	19.3 ^{b}, CH3	19.3 ^{a}, CH3
8′	173.3, C	174.1, C	8″	173.7, C	173.6, C
9′	47.4, CH2	53.0, CH2	9″	47.7, CH2	47.5, CH2
10′	23.0, CH2	208.9, C	10″	22.8, CH2	23.0, CH2
11′	31.0, CH2	42.0, CH2	11″	31.3, CH2	31.1, CH2
12′	56.4, CH	54.4, CH	12″	56.6, CH	56.6, CH
13′	173.4 ^{c}, C	172.9, C	13″	173.5 ^{c}, C	173.6, C
14′	35.0, CH3	34.9 ^{b}, CH3	14″	35.0, CH3	35.0 ^{b}, CH3
15′	51.3, CH2	51.3, CH2	15″	51.4, CH2	51.4, CH2
16′	166.4, C	166.1, C	16″	166.7 ^{a}, C	166.8, C
17′	39.3, CH3	39.4, CH3	17″	39.2, CH3	39.2, CH3
18′	71.3, CH	71.5 ^{c}, CH	18″	71.2, CH	71.2 ^{c}, CH
19′	27.0, CH	27.1 ^{d}, CH	19″	27.0, CH	27.2 ^{d}, CH
20′	19.1 ^{d}, CH3	19.0 ^{e}, CH3	20″	19.1 ^{d} CH3	19.1 ^{e}, CH3
21′	21.6 ^{d}, CH3	21.6 ^{e,} CH3	21″	21.7 ^{d}, CH3	21.7 ^{e}, CH3
22′	167.7, C	167.7, C	22″	167.8, C	167.7, C
23′	75.0, CH	74.7, CH	23″	75.1, CH	74.8, CH
24′	17.3, CH3	17.3, CH3	24″	17.8, CH3	17.8, CH3

$^{a–e}$ The data with the same labels in each column may be interchanged.

Table S3. ^{1}H NMR data of actinomycins D (**1**) and V (**2**) (in $CDCl_3$-*d*).

No.	1 (*J* = Hz)	2 (*J* = Hz)	No.	1 (*J* = Hz)	2 (*J* = Hz)
3	7.56, d (7.8)	7.61, d (7.7)	4	7.33, d (7.8)	7.36, d (7.7)
2′	4.47, dd (6.9, 2.4)	4.56, dd (7.3, 2.7)	2″	4.56, dd (6.7, 2.4)	4.49, dd (6.6, 2.6)
NH-2′	7.14, d (7.0)	7.19, d (7.3)	NH-2″	7.69, d (7.0)	7.68, d (7.2)
4′	3.49, dd (10.0, 6.0)	3.57, dd (9.5, 6.0)	4″	3.52, dd (10.0, 6.1)	3.70, dd (9.8, 6.0)
NH-4′	8.13, d (5.9)	7.68, d (7.2)	NH-4″	7.94, d (6.1)	8.21, d (6.0)
5′	2.14, m	2.13, m	5″	2.08, m	2.23, m
6′	1.06, d (6.8) [a]	1.12, d (6.8)[a]	6″	1.05, d (6.8) [a]	1.14, d (6.8) [a]
7′	0.82, d (6.8) [a]	0.90, d (6.8)[a]	7"	0.84, d (6.8) [a]	0.91, d (6.8) [a]
9′	3.68, m; 3.93, m	3.96, d (19.5); 4.55, d (19.5)	9″	3.65, m; 3.78, m	3.73, m; 3.92, m
10′	2.05, m; 2.20, m	–	10″	2.05, m; 2.20, m	2.21, m; 2.27, m
11′	1.77, m; 2.62, m	2,31, d (17.5); 3.63, d (17.5)	11″	1.81, m; 2.89, m	1.87, m; 2.76, m
12′	5.96, d (9.2)	6.56, d (10.0)	12″	5.88, d (9.2)	5.96, d (9.3)
14′	2.82, s	2.89 [b], s	14″	2.82, s	2.92 [b], s
15′	3.62, d (17.5); 4.67, d (17.5)	3.69, d (17.7) 4.58, d (17.7)	15″	3.58, d (17.5); 4.77, d (17.5)	3.66, d (17.5) 4.71, d (17.5)
17′	2.90, s	2.94, s	17″	2.87, s	2.93, s
18′	2.66, d (9.4)	2.69, d (9.6) [c]	18″	2.66, d (9.4)	2.71, d (9.6) [c]
19′	2.59, m	2.65, m	19″	2.59, m	2.65, m
20′	0.69, d (6.7) [b]	0.74, d (6.5) [d]	20″	0.69, d (6.7) [b]	0.75, d (6.5) [d]
21′	0.89, d (6.7) [b]	0.95, d (6.3) [d]	21″	0.91, d (6.7) [b]	0.98, d (6.3) [d]
23′	5.15, dd (6.5, 2.6)	5.24, dd (6.2, 2.6)	23″	5.11, dd (6.5, 2.6)	5.15, dd (6.2, 2.6)
24′	1.20, d (6.3)	1.26, d (6.7)	24″	1.20, d (6.3)	1.12, d (6.7)

[a–d] The data with the same labels in each column may be interchanged.

Table S4. Main ^{1}H NMR data of actinomycin X0$_\beta$ (**3**, in $CDCl_3$-*d*).

No.	3 (*J* = Hz)	No.	3 (*J* = Hz)
3	7.66, d (7.8)	4	7.36, d (7.8)
2′	4.84, dd (7.0, 2.4)	2″	4.50, dd (7.0, 2.4)
NH-2′	7.44, d (6.5)	NH-2″	7.92, d (7.5)
4′	3.56, dd (10.3, 5.5)	4″	3.74, dd (10.1, 6.5)
NH-4′	7.48, d (6.9)	NH-4″	8.20 d (5.6)
6′	1.12, d (6.7) [a]	6″	1.14, d (6.7) [a]
7′	0.86, d (6.7) [a]	7″	0.91, d (6.7) [a]
12′	6.05, dd (9.3, 3.0)	12″	5.98, d (9.0)
14′	2.90, s	14″	2.90, s
15′	3.60, d (17.5); 4.56, d (17.5)	15″	3.64, d (17.6); 4.74, d (17.6)
17′	2.96 [b], s	17″	2.97 [b], s
18′	2.66, d (9.0)	18″	2.68, d (9.3)
19′	2.66, m	19″	2.66, m
20′	0.75, d (6.6) [c]	20″	0.76, d (6.7) [c]
21′	0.96, d (6.7) [c]	21″	0.98, d (6.7) [c]
23′	5.25, m	23″	5.25, m
24′	1.30, d (6.4) [d]	24″	1.26, d (6.1)[d]

[a–d] The data with the same labels in each column may be interchanged.

TTCGAAGCTCCCTCCCACAAGGGGTTGGGCCACCGGCTTCGGGTGTTACCGACTTTCGTG
ACGTGACGGGCGGTGTGTACAAGGCCCGGGAACGTATTCACCGCAGCAATGCTGATCTG
CGATTACTAGCAACTCCGACTTCATGGGGTCGAGTTGCAGACCCCAATCCGAACTGAGAC
CGGCTTTTTGAGATTCGCTCCGCCTCACGGCATCGCAGCTCATTGTACCGGCCATTGTAGC
ACGTGTGCAGCCCAAGACATAAGGGGCATGATGACTTGACGTCGTCCCCACCTTCCTCCG
AGTTGACCCCGGCAGTCTCCTGTGAGTCCCCATCACCCCGAAGGGCATGCTGGCAACAC
AGAACAAGGGTTGCGCTCGTTGCGGGACTTAACCCAACATCTCACGACACGAGCTGACG
ACAGCCATGCACCACCTGTATACCGACCACAAGGGGGGCACCATCTCTGATGCTTTCCGG
TATATGTCAAGCCTTGGTAAGGTTCTTCGCGTTGCGTCGAATTAAGCCACATGCTCCGCTG
CTTGTGCGGGCCCCCGTCAATTCCTTTGAGTTTTAGCCTTGCGGCCGTACTCCCCAGGCG
GGGAACTTAATGCGTTAGCTGCGGCACCGACGACGTGGAATGTCGCCAACACCTAGTTCC
CAACGTTTACGGCGTGGACTACCAGGGTATCTAATCCTGTTCGCTCCCCACGCTTTCGCTC
CTCAGCGTCAGTAATGGCCCAGAGATCCGCCTTCGCCACCGGTGTTCCTCCTGATATCTGC
GCATTTCACCGCTACACCAGGAATTCCGATCTCCCCTACCACACTCTAGCTAGCCCGTATC
GAATGCAGACCCGGGGTTAAGCCCCGGGCTTTCACATCCGACGTGACAAGCCGCCTACG
AGCTCTTTACGCCCAATAATTCCGGACAACGCTTGCGCCCTACGTATTACCGCGGCTGCTG
GCACGTAGTTAGCCGGCGCTTCTTCTGCAGGTACCGTCACTTTCGCTTCTTCCCTGCTGAA
AGAGGTTTACAACCCGAAGGCCGTCATCCCTCACGCGGCGTCGCTGCATCAGGCTTTCGC
CCATTGTGCAATATTCCCCACTGCTGCCTCCCGTAGGAGTCTGGGCCGTGTCTCAGTCCCA
GTGTGGCCGGTCGCCCTCTCAGGCCGGCTACCCGTCGTCGCCTTGGTAGGCCATTACCCC
ACCAACAAGCTGATAGGCCGCGGGCTCATCCTTCACCGCCGGAGCTTTTAACCCCGTCCC
ATGCGGGACAGAGTGTTATCCGGTATTAGACCCCGTTTCCAGGGCTTGTCCCAGAGTGAA
GGGCAGATTGCCCACGTGTTACTCACCCGTTCGCCACTAATCCACCCCGAAAGGCTTCAT
CGTTCG ACTGCA

Figure S1. 16S rDNA sequence of *Streptomyces* sp. ZQ388.

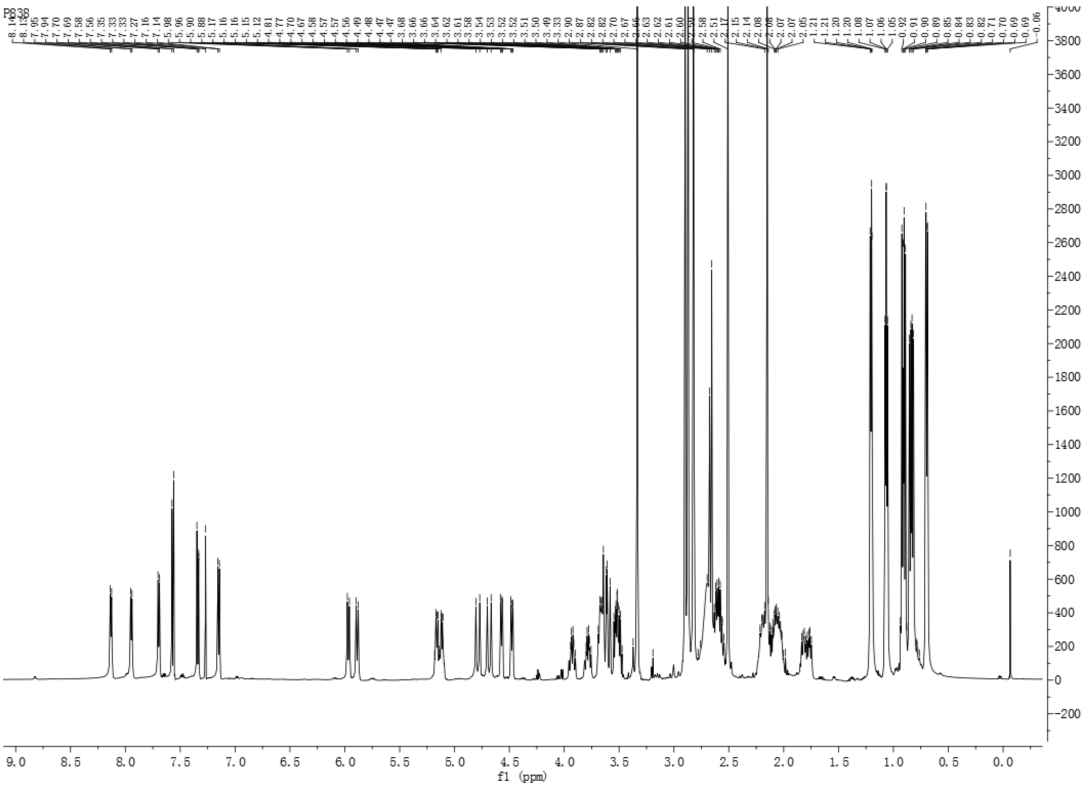

Figure S2. ^{1}H NMR spectrum of actinomycin D (**1**, in $CDCl_3$-*d*).

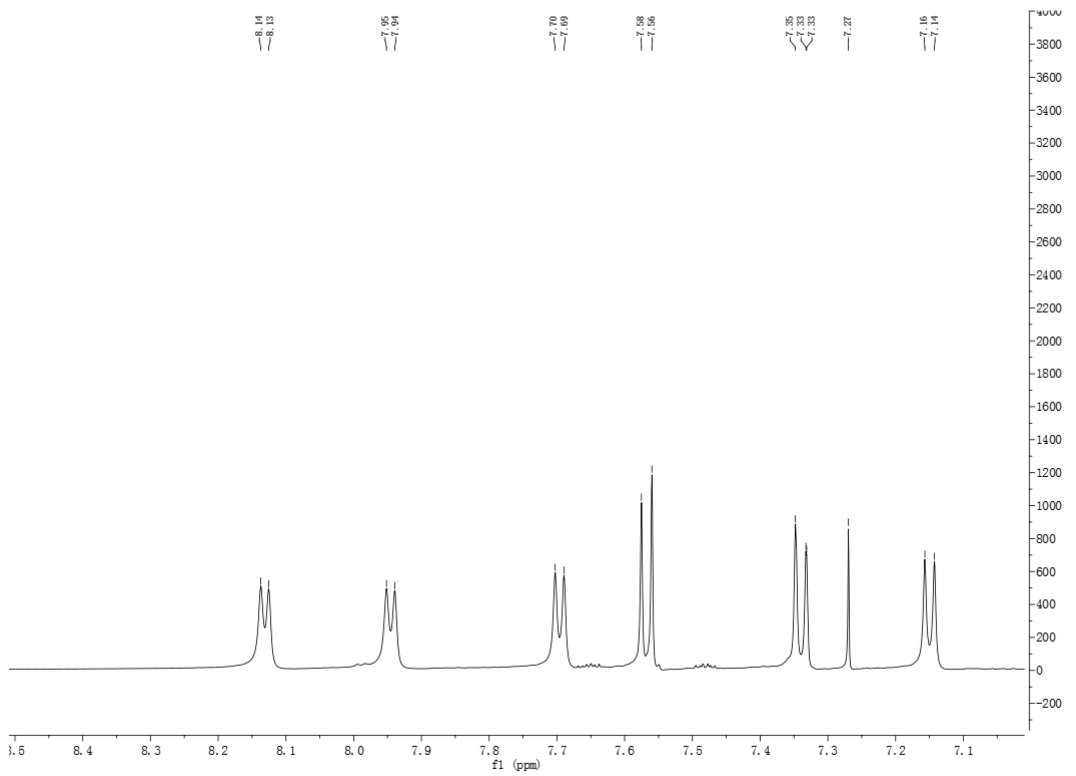

Figure S3. ^{1}H NMR spectrum of actinomycin D (**1**, in $CDCl_3$-*d*).

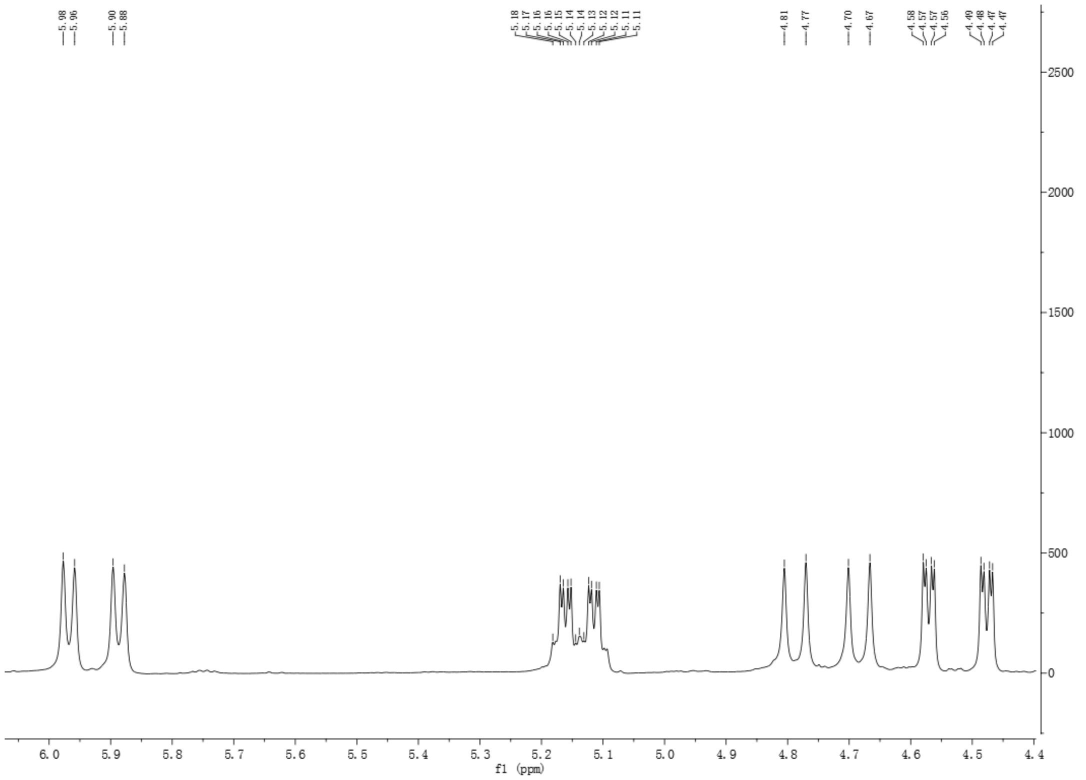

Figure S4. ^{1}H NMR spectrum of actinomycin D (**1**, in $CDCl_3$-*d*).

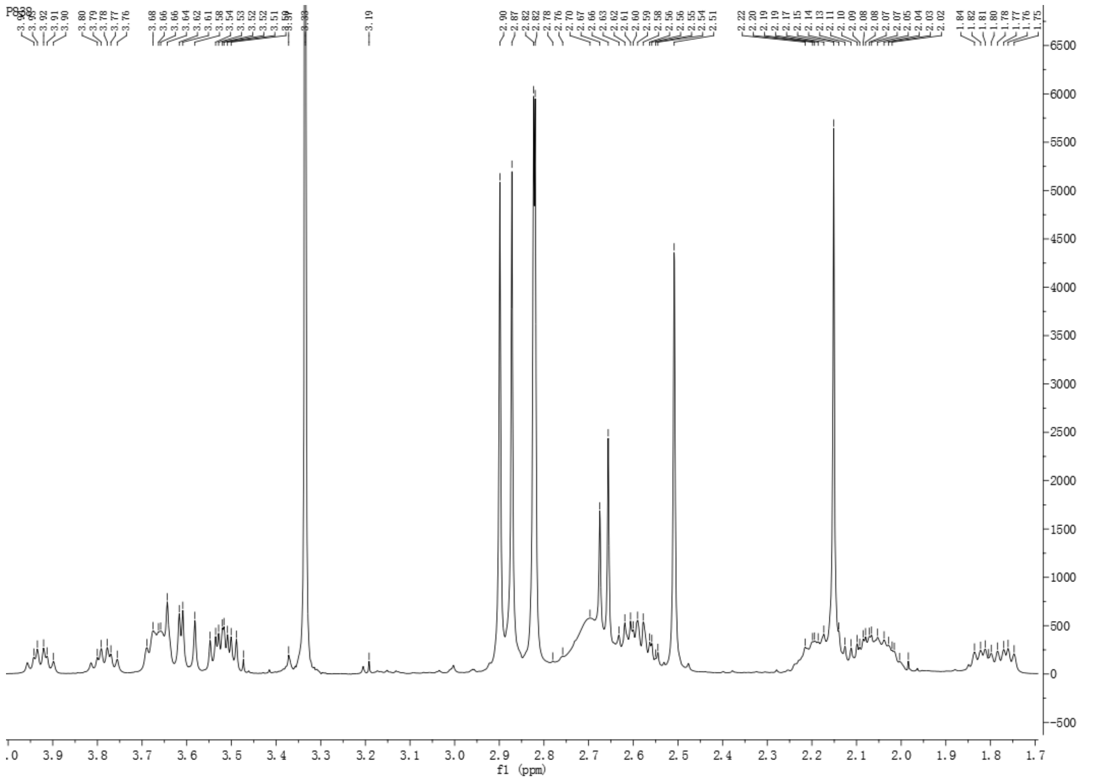

Figure S5. ^{1}H NMR spectrum of actinomycin D (**1**, in $CDCl_3$-*d*).

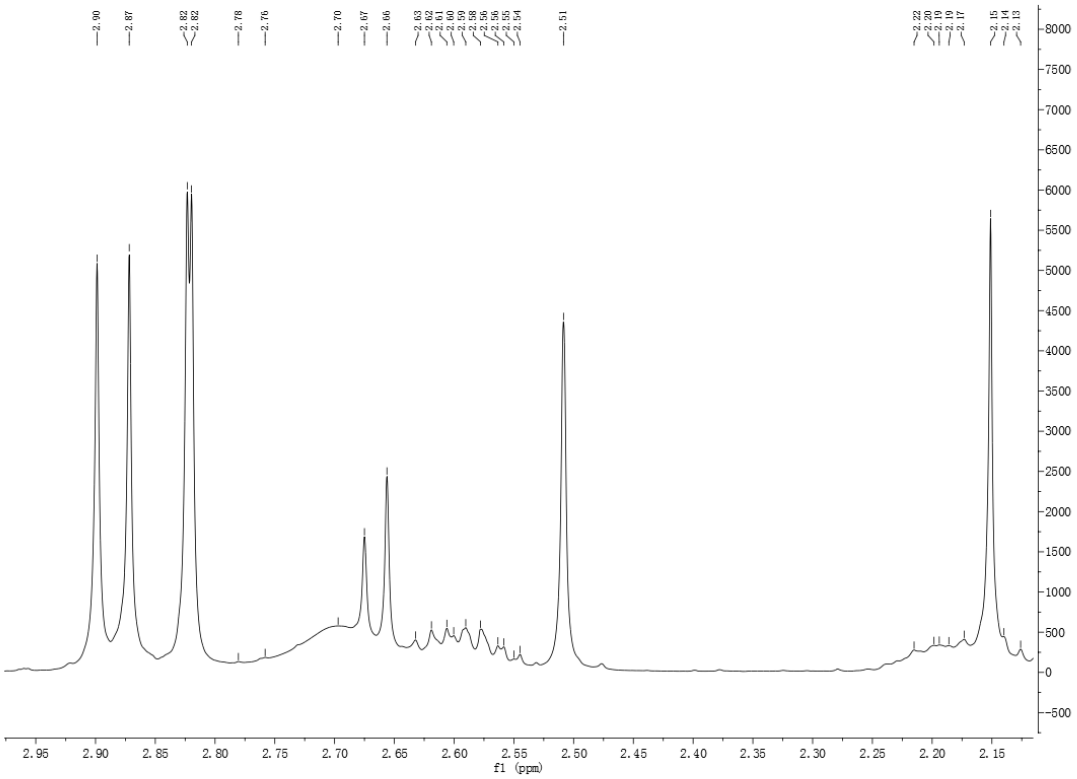

Figure S6. ^{1}H NMR spectrum of actinomycin D (**1**, in $CDCl_3$-*d*).

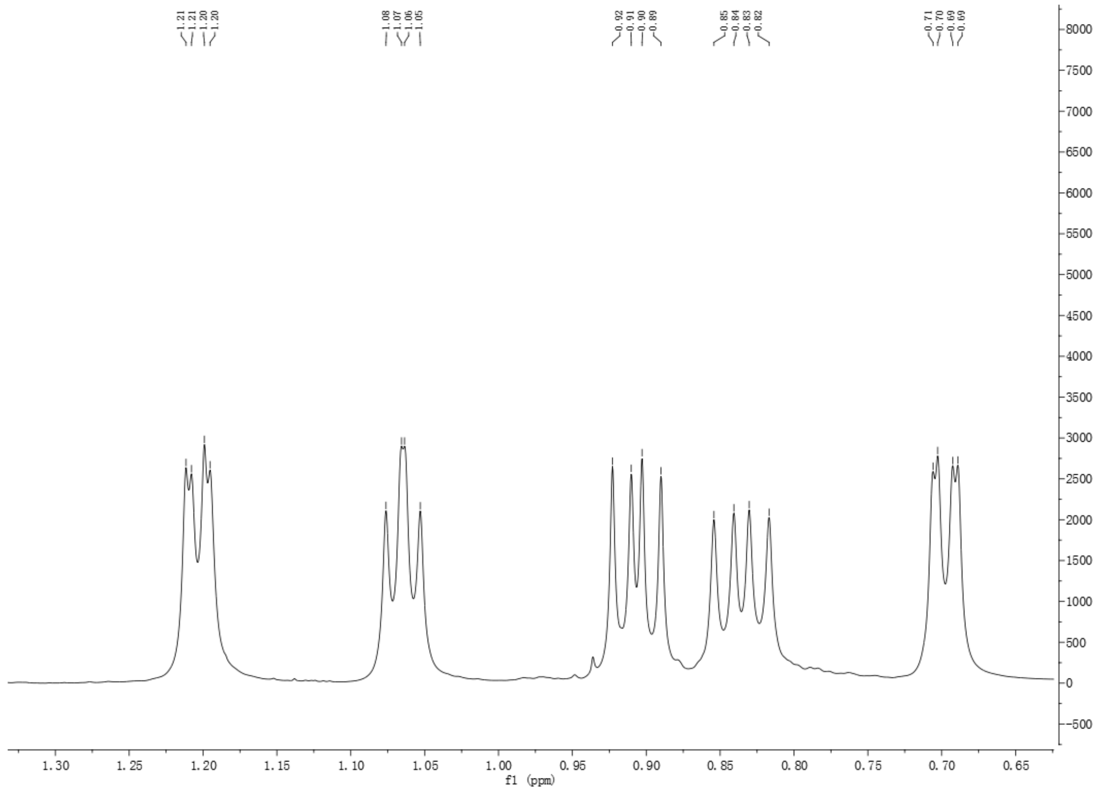

Figure S7. ^{1}H NMR spectrum of actinomycin D (**1**, in $CDCl_3$-*d*).

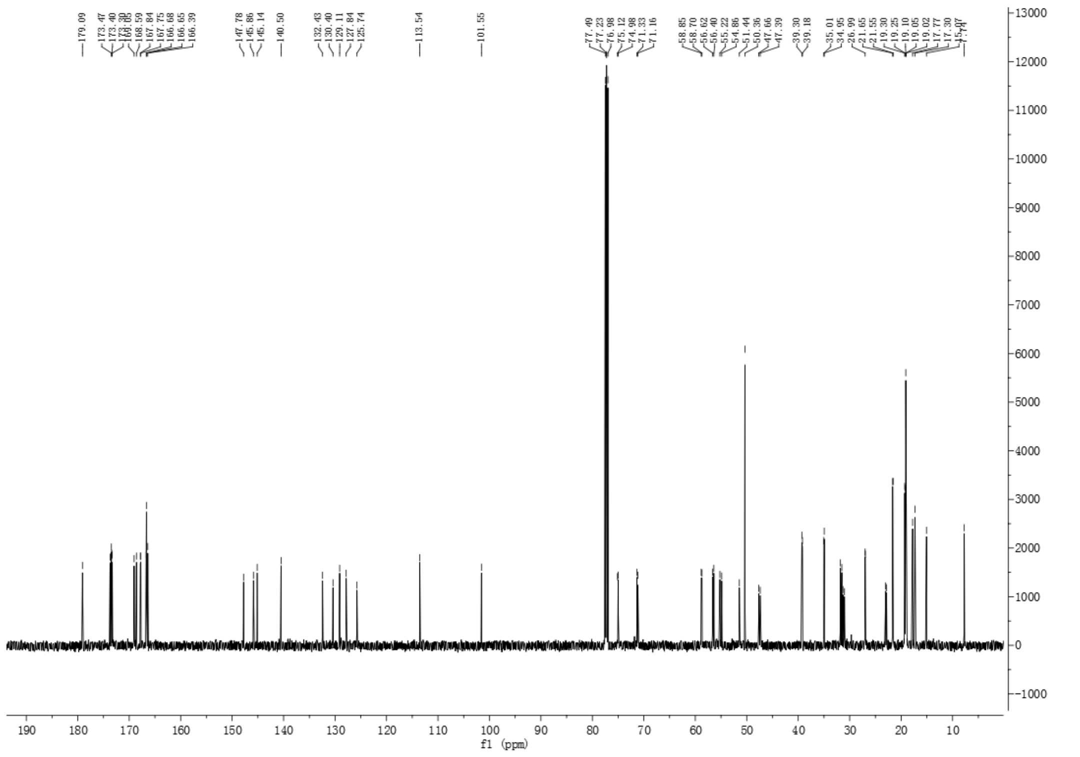

Figure S8. ^{13}C NMR spectrum of actinomycin D (**1**, in $CDCl_3$-*d*).

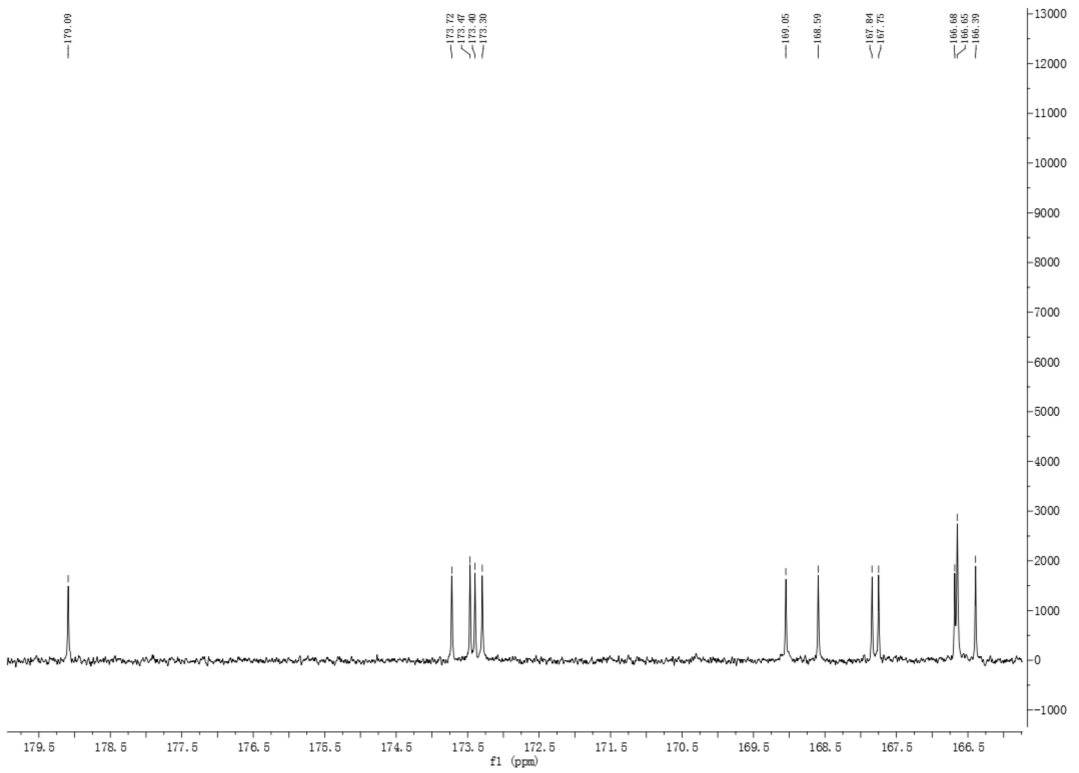

Figure S9. ^{13}C NMR spectrum of actinomycin D (**1**, in $CDCl_3$-*d*).

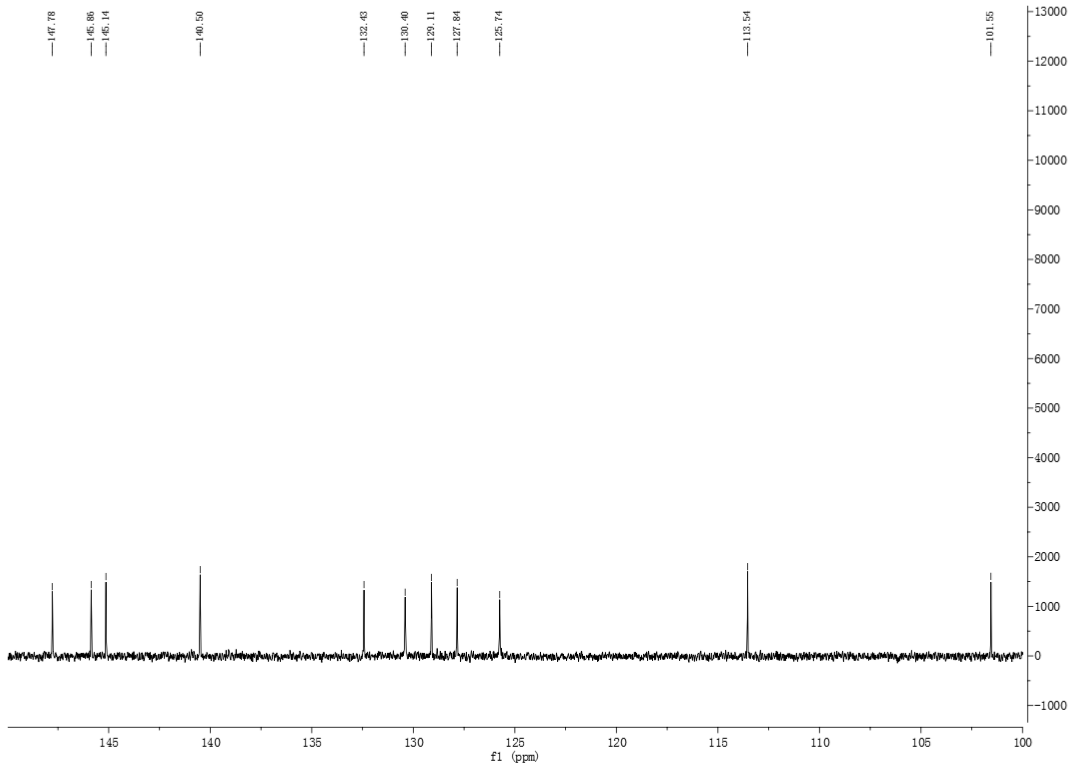

Figure S10. ^{13}C NMR spectrum of actinomycin D (**1**, in $CDCl_3$-*d*).

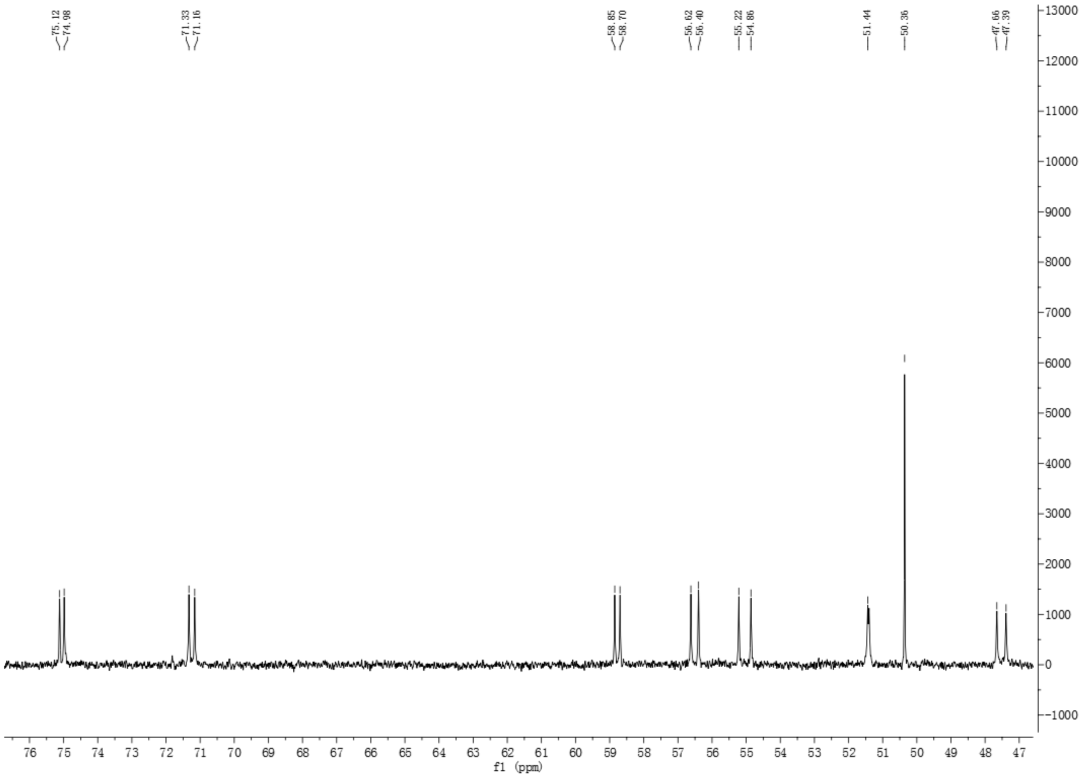

Figure S11. ^{13}C NMR spectrum of actinomycin D (**1**, in $CDCl_3$-*d*).

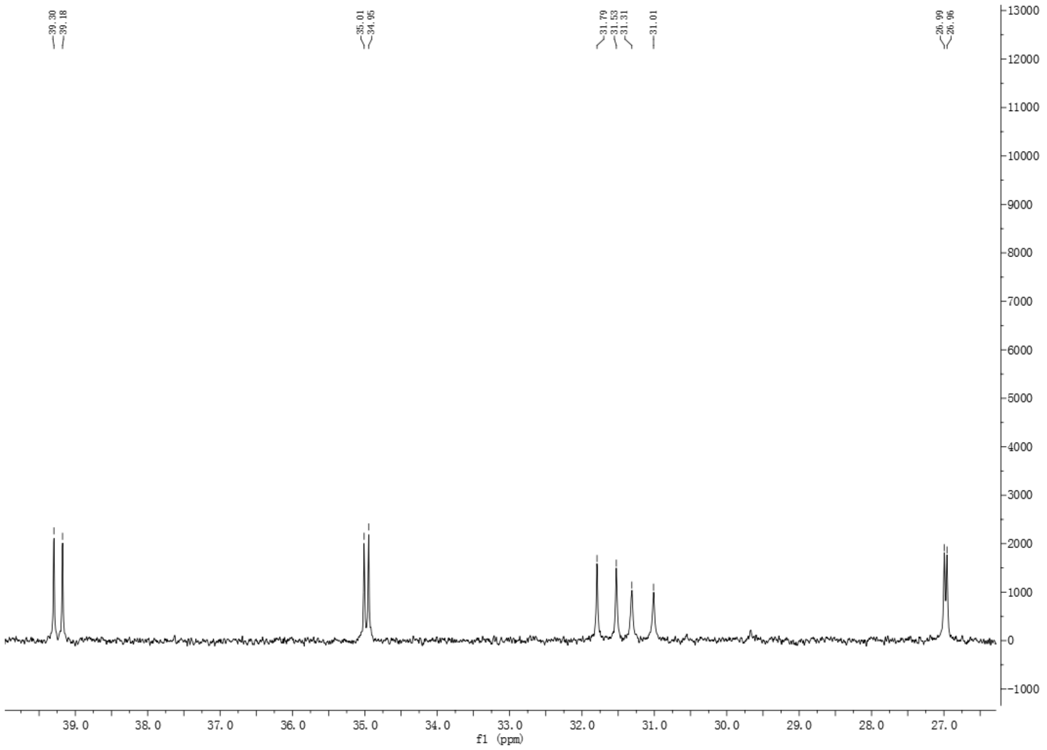

Figure S12. ^{13}C NMR spectrum of actinomycin D (**1**, in $CDCl_3$-*d*).

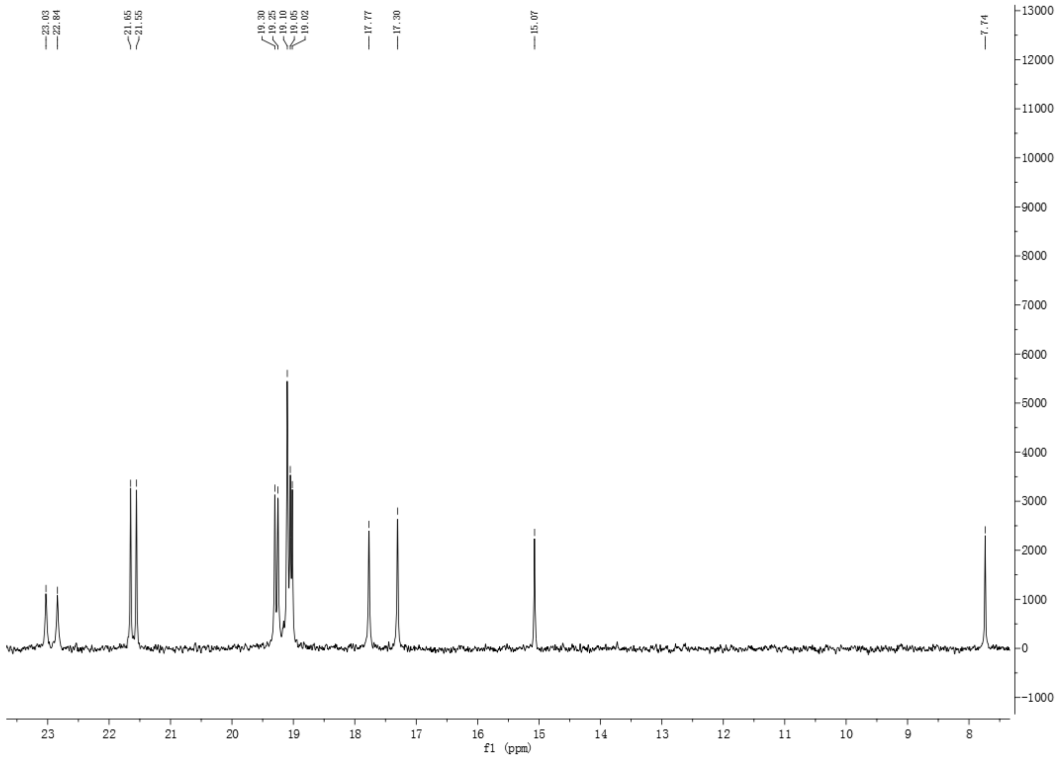

Figure S13. ^{13}C NMR spectrum of actinomycin D (**1**, in $CDCl_3$-*d*).

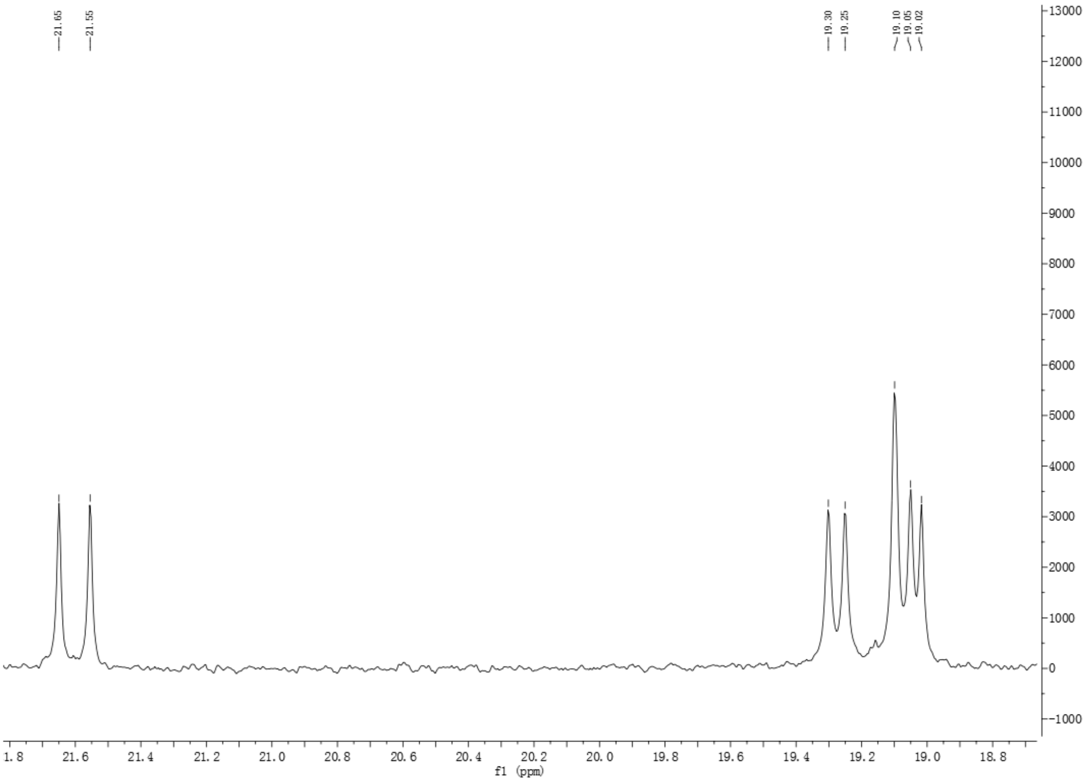

Figure S14. ^{13}C NMR spectrum of actinomycin D (**1**, in $CDCl_3$-*d*).

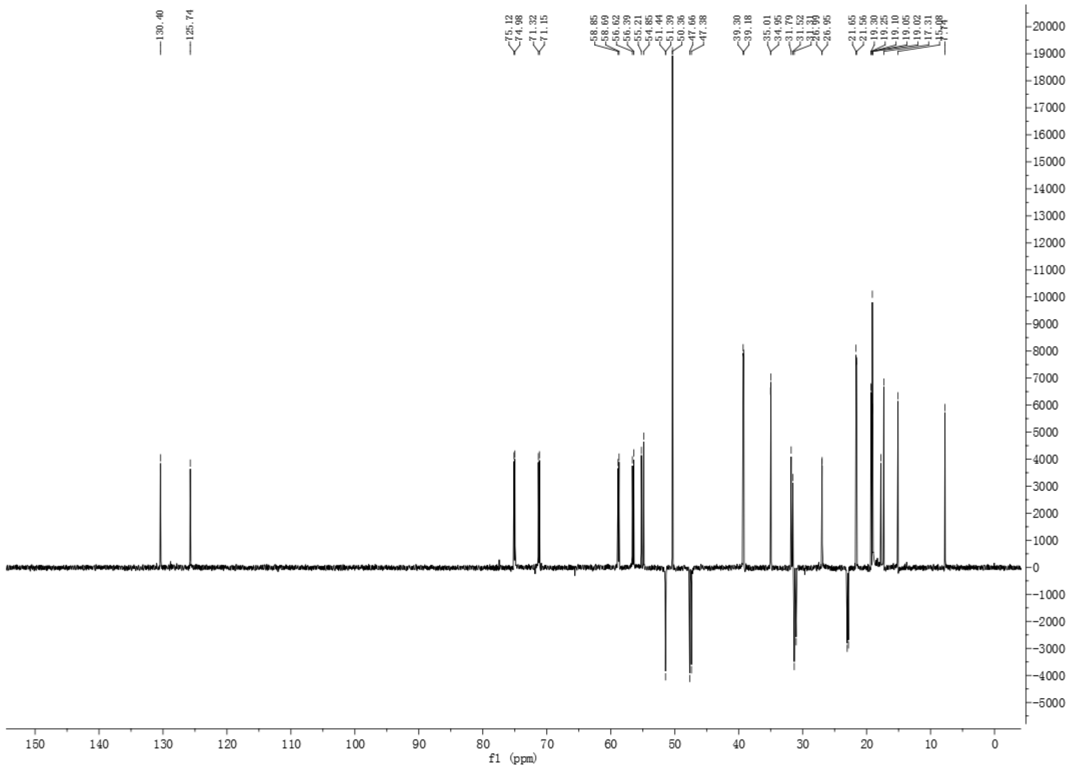

Figure S15. DEPT spectrum of actinomycin D (**1**, in $CDCl_3$-*d*).

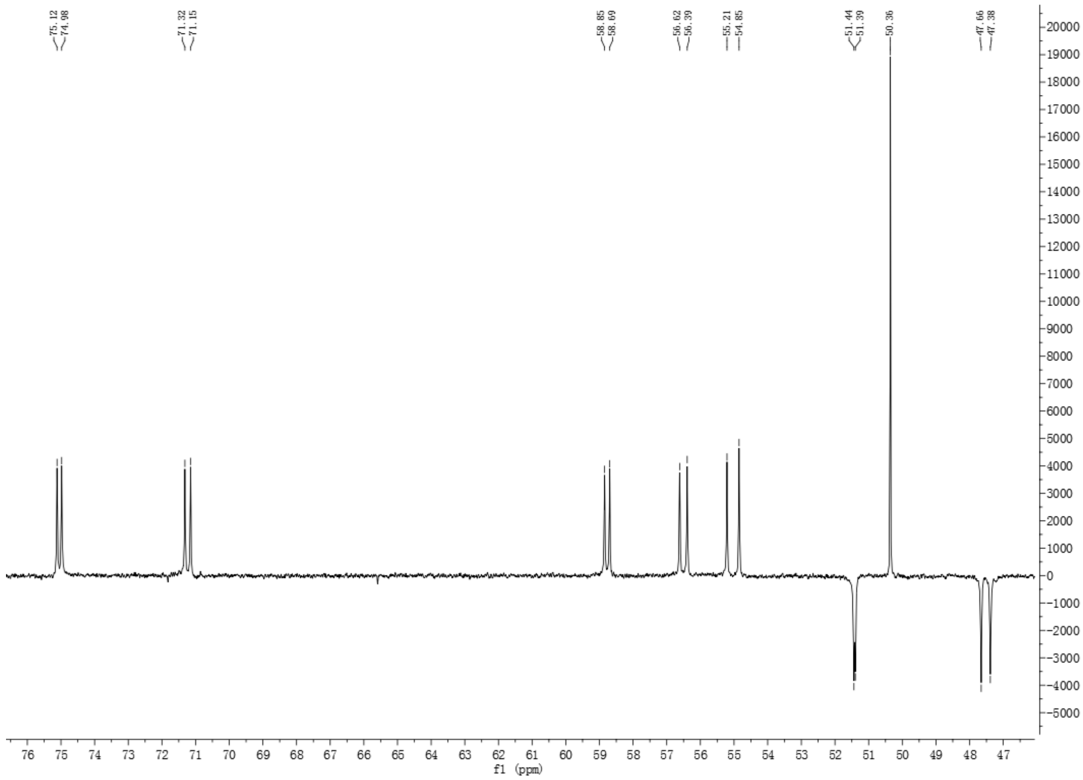

Figure S16. DEPT spectrum of actinomycin D (**1**, in $CDCl_3$-*d*).

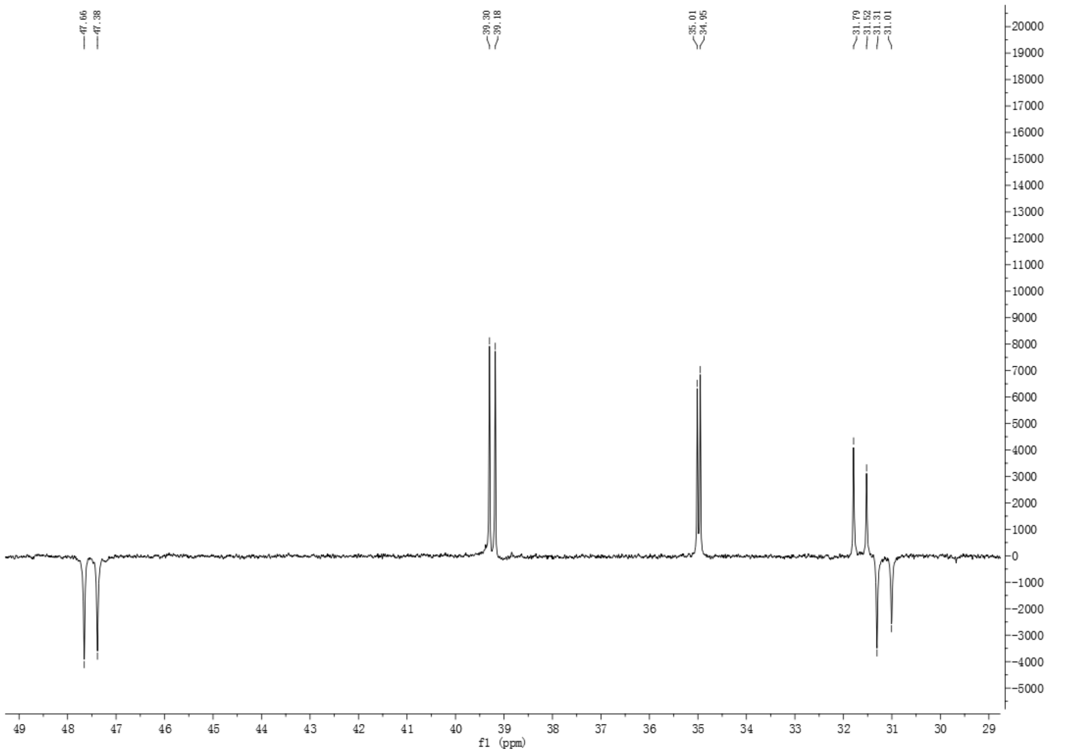

Figure S17. DEPT spectrum of actinomycin D (**1**, in CDCl3-*d*).

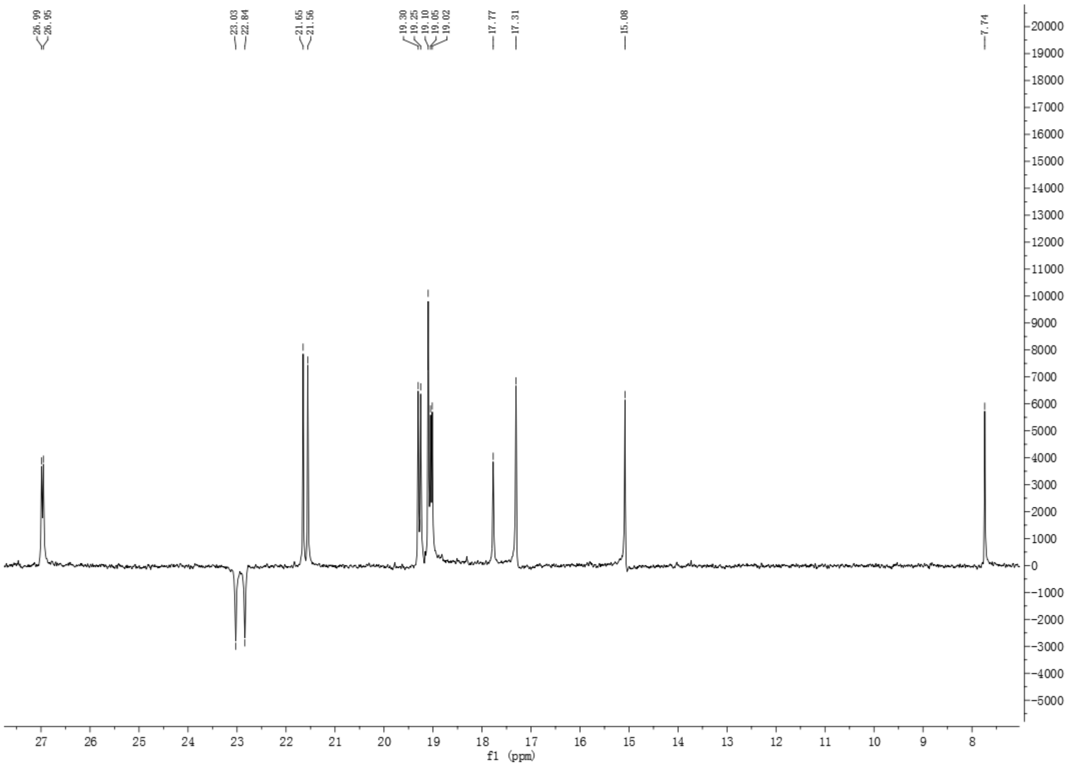

Figure S18. DEPT spectrum of actinomycin D (**1**, in CDCl3-*d*).

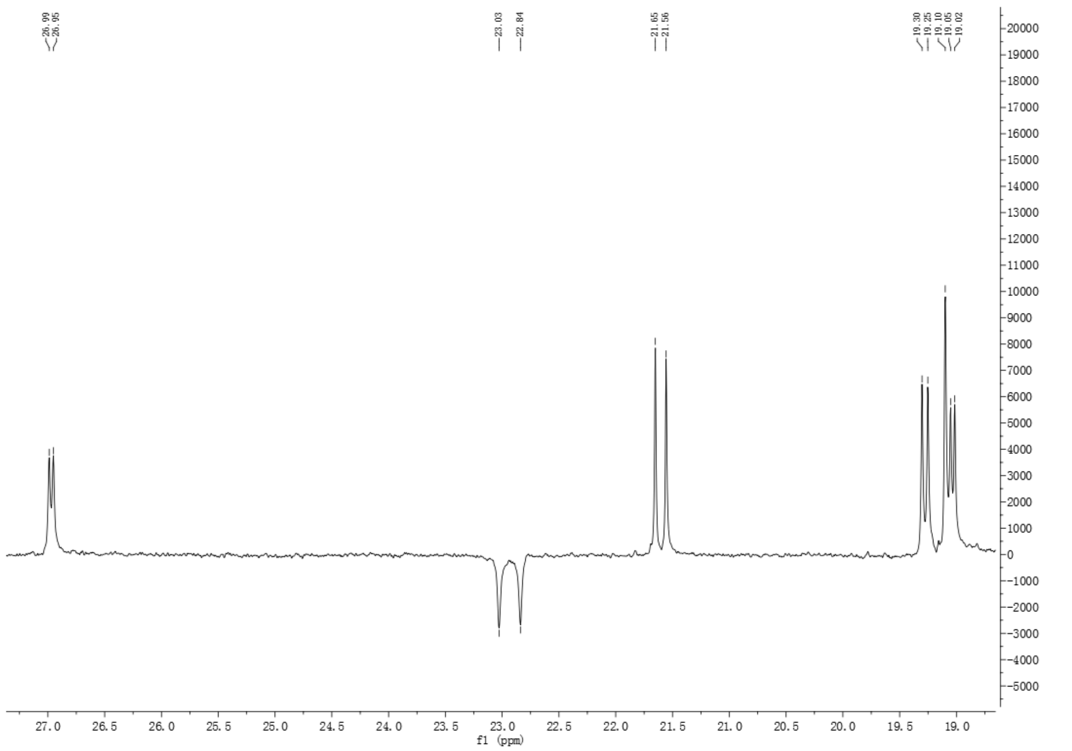

Figure S19. DEPT spectrum of actinomycin D (**1**, in $CDCl_3$-*d*).

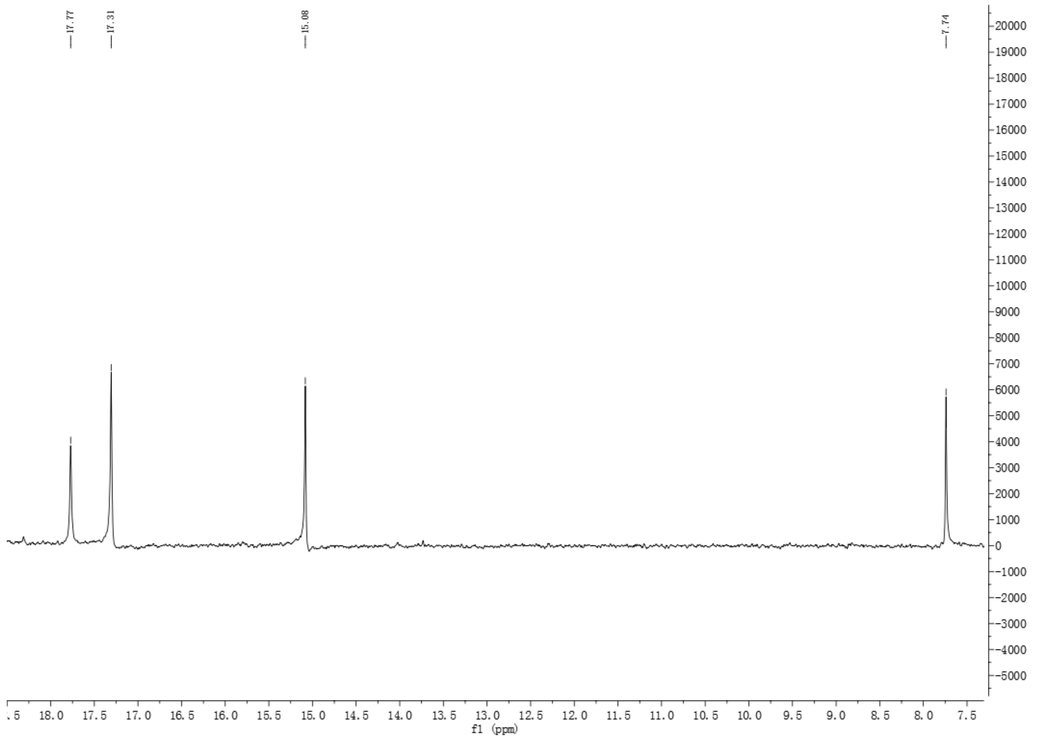

Figure S20. DEPT spectrum of actinomycin D (**1**, in $CDCl_3$-*d*).

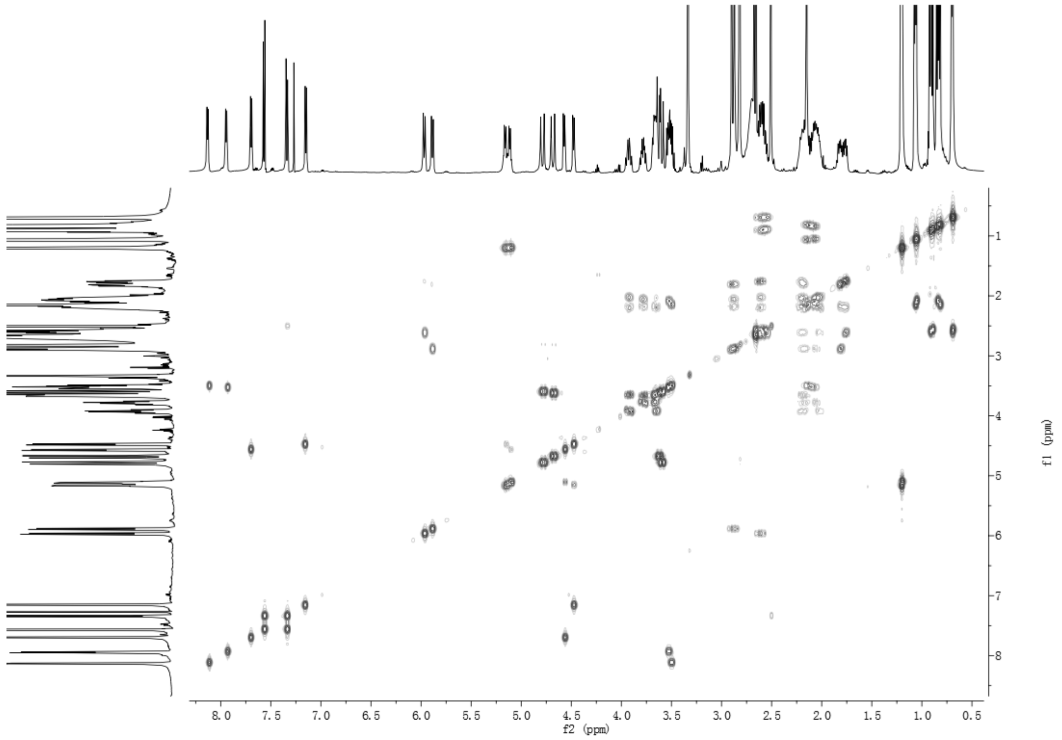

Figure S21. ^{1}H–^{1}H COSY spectrum of actinomycin D (**1**, in $CDCl_3$-*d*).

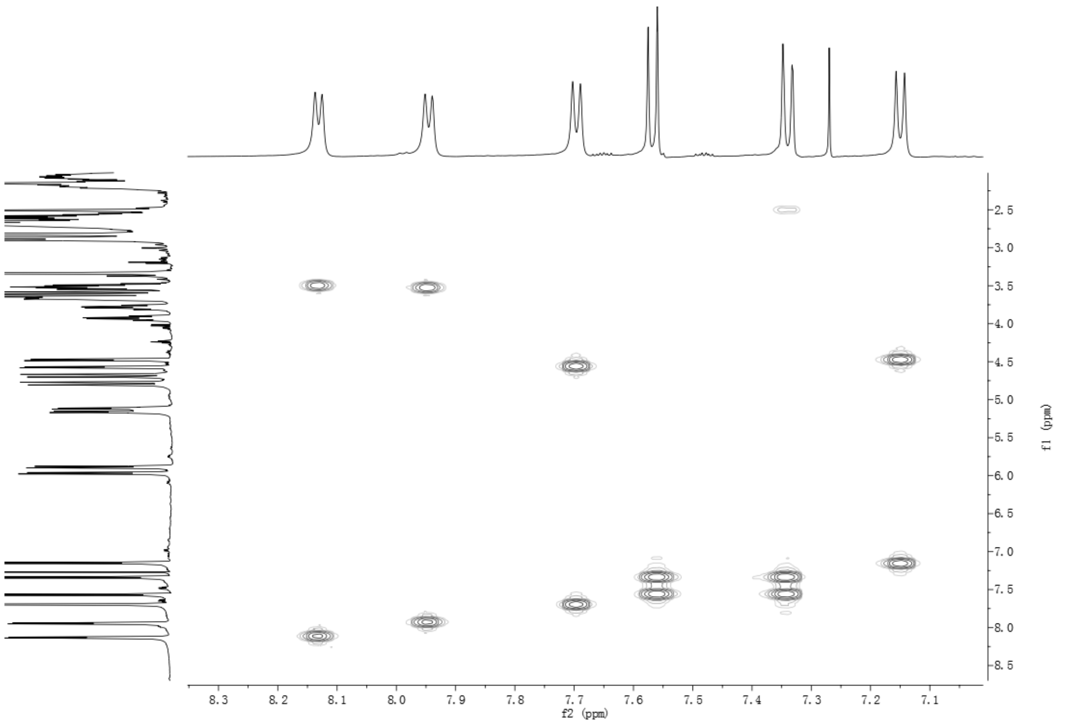

Figure S22. ^{1}H–^{1}H COSY spectrum of actinomycin D (**1**, in $CDCl_3$-*d*).

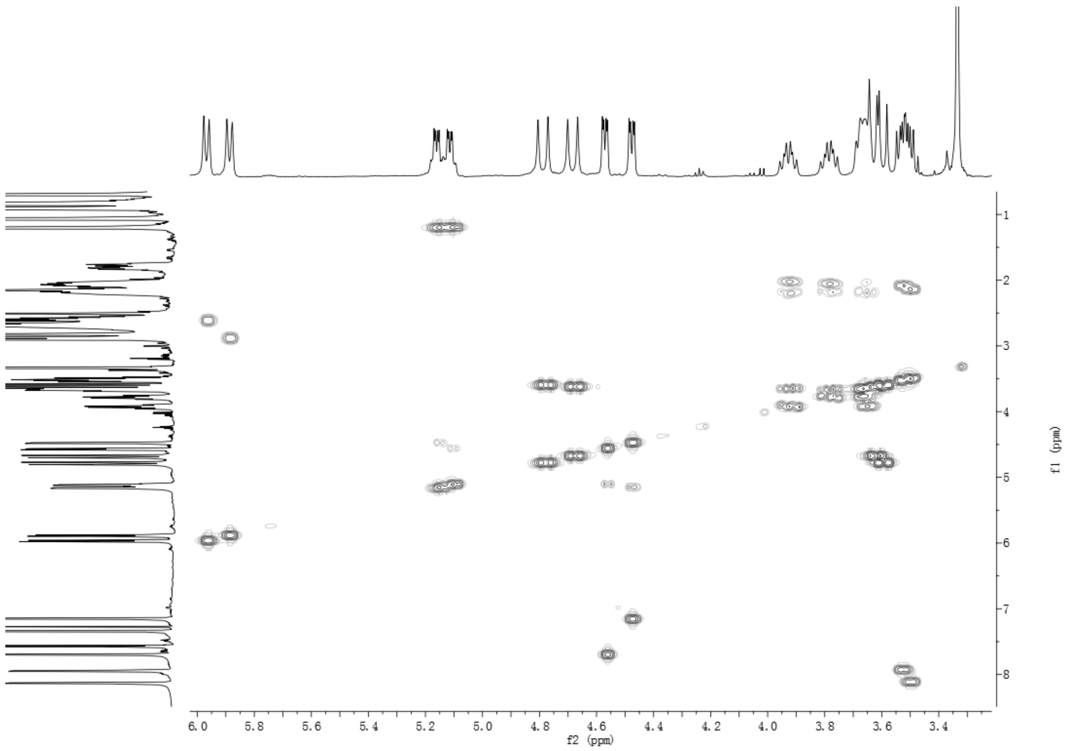

Figure S23. ^{1}H–^{1}H COSY spectrum of actinomycin D (**1**, in $CDCl_3$-*d*).

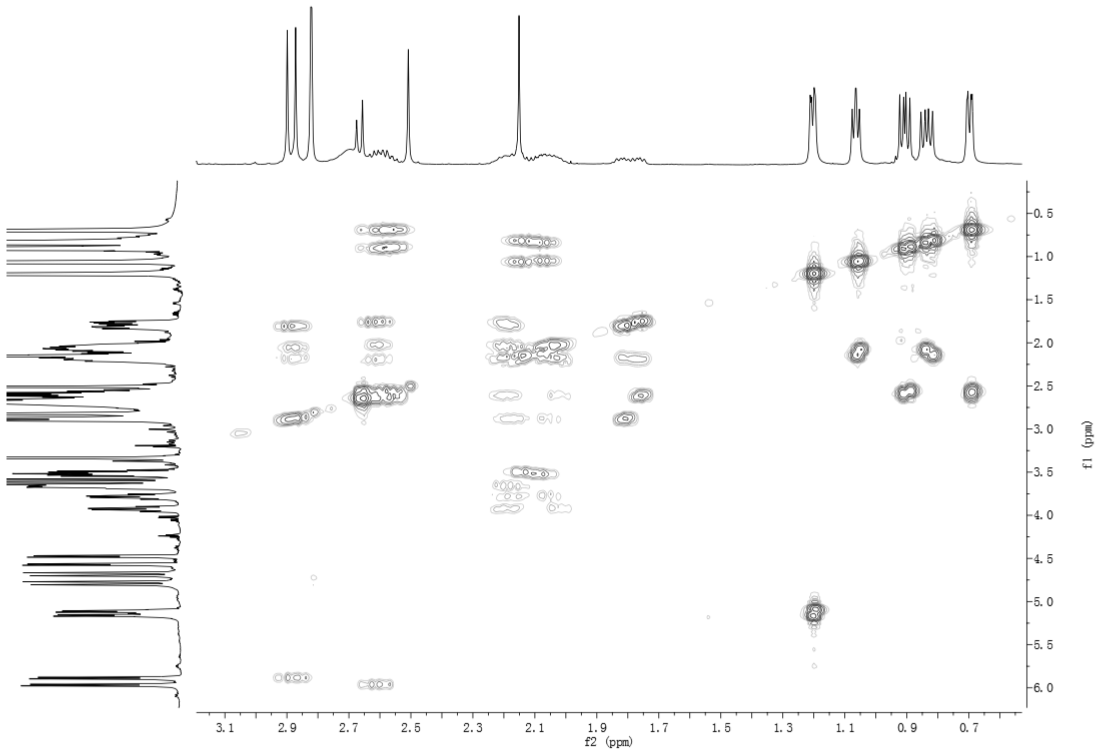

Figure S24. ^{1}H–^{1}H COSY spectrum of **1** actinomycin D (**1**, in $CDCl_3$-*d*).

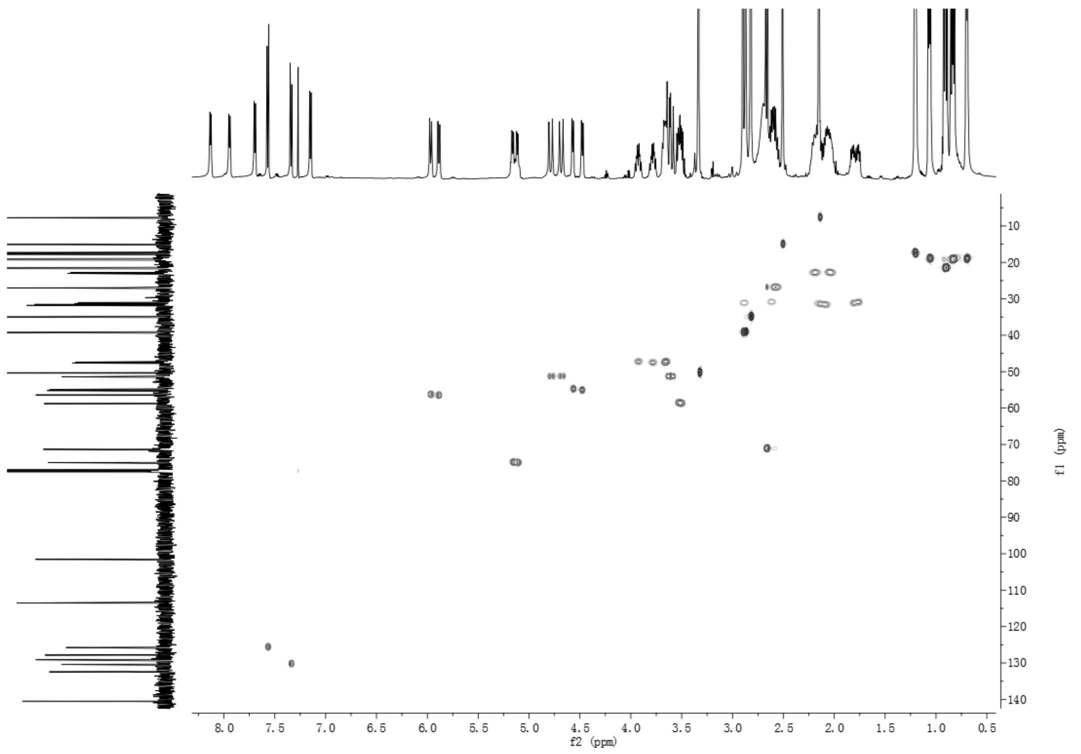

Figure S25. HSQC spectrum of actinomycin D (**1**, in $CDCl_3$-*d*).

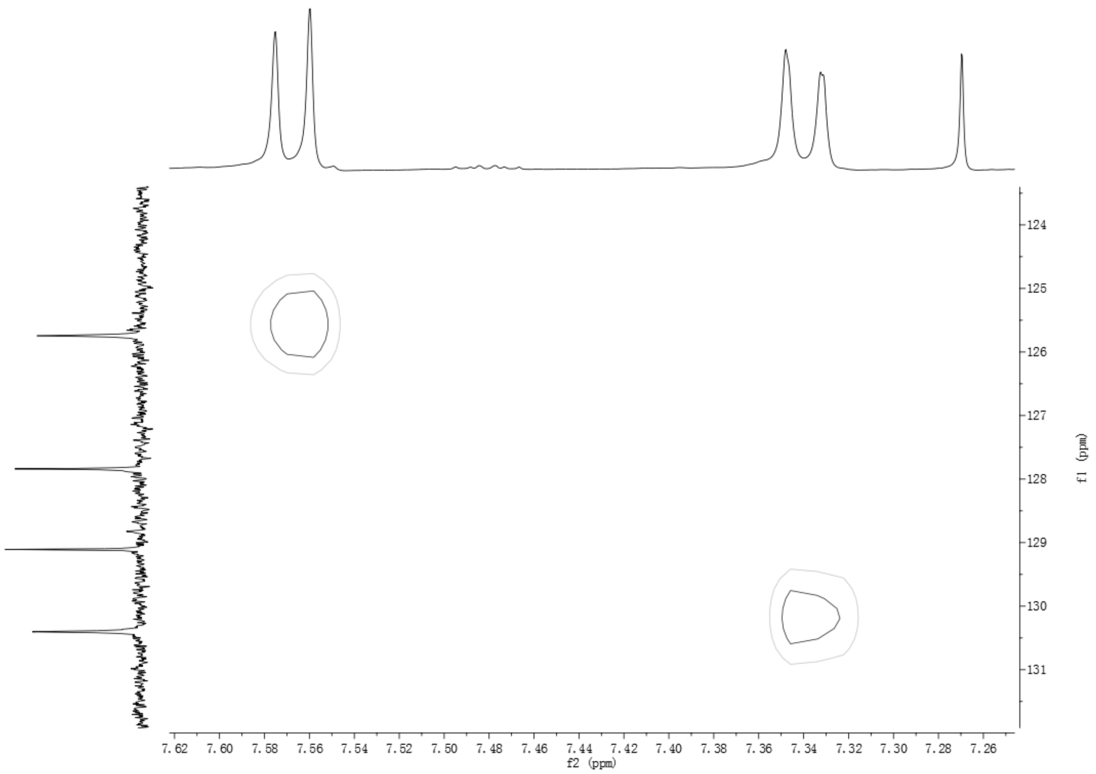

Figure S26. HSQC spectrum of actinomycin D (**1**, in $CDCl_3$-*d*).

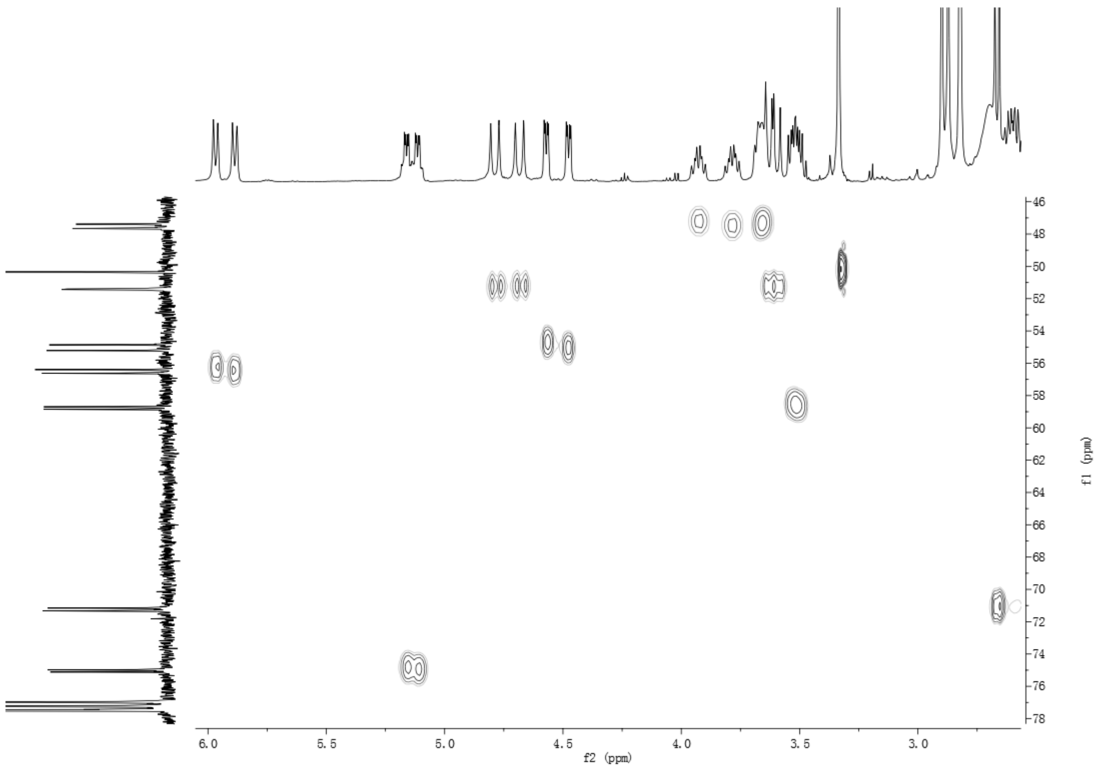

Figure S27. HSQC spectrum of actinomycin D (**1**, in $CDCl_3$-*d*).

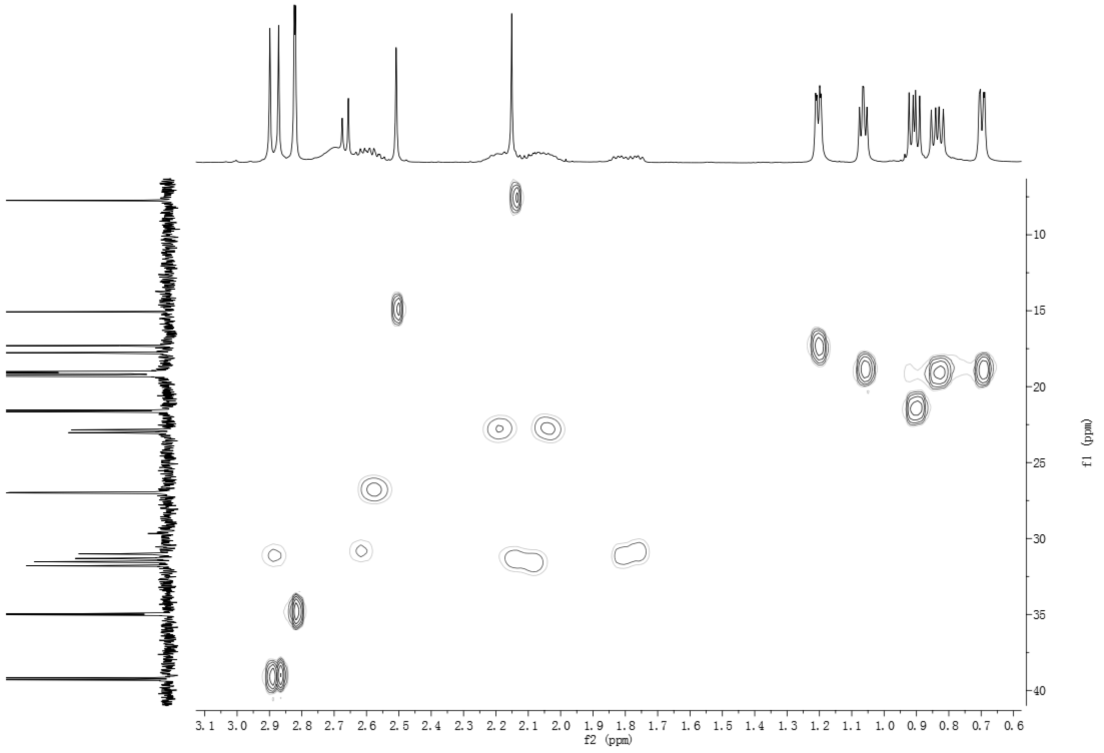

Figure S28. HSQC spectrum of actinomycin D (**1**, in $CDCl_3$-*d*).

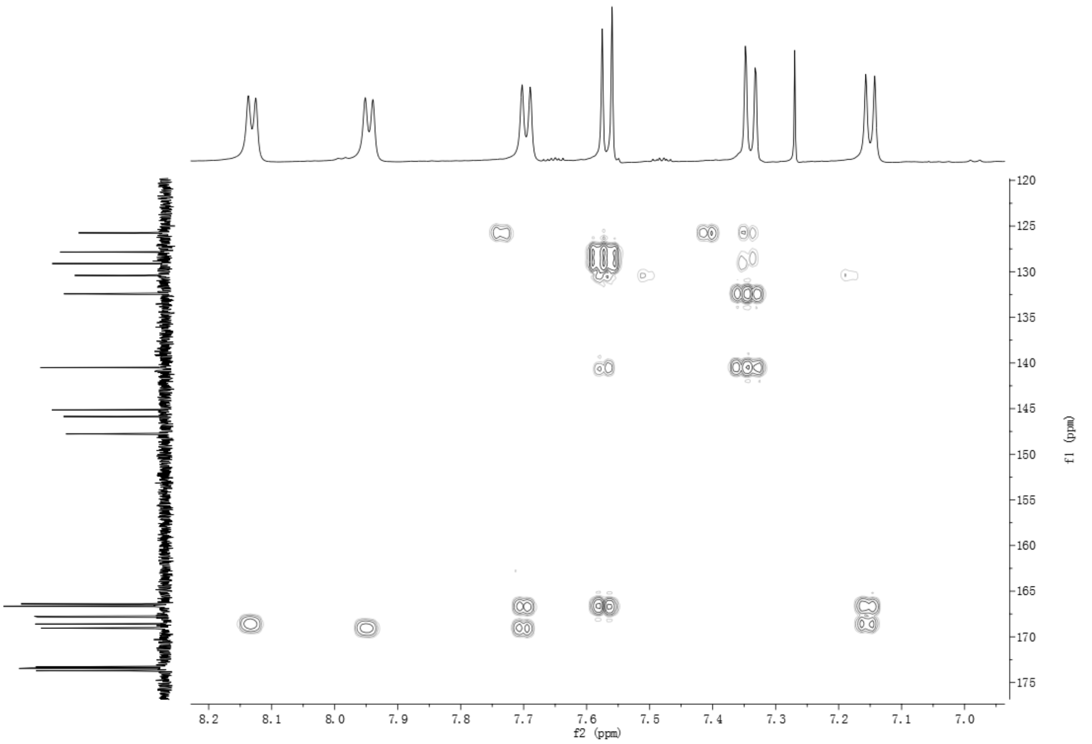

Figure S29. HMBC spectrum of actinomycin D (**1**, in $CDCl_3$-*d*).

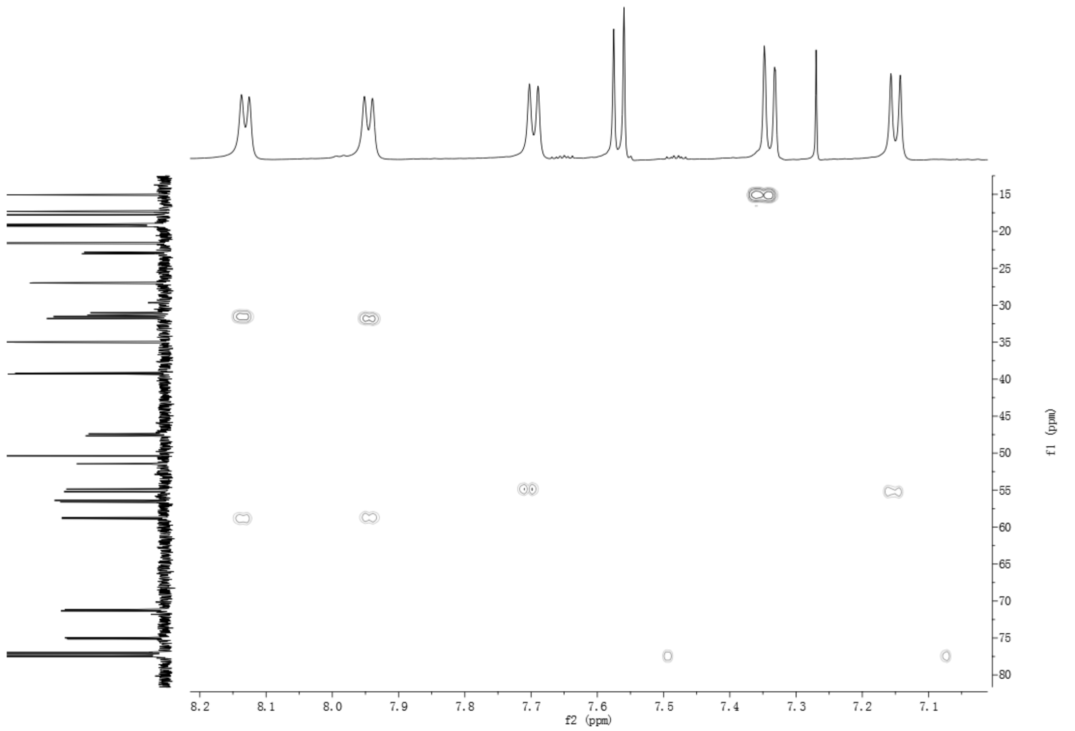

Figure S30. HMBC spectrum of actinomycin D (**1**, in $CDCl_3$-*d*).

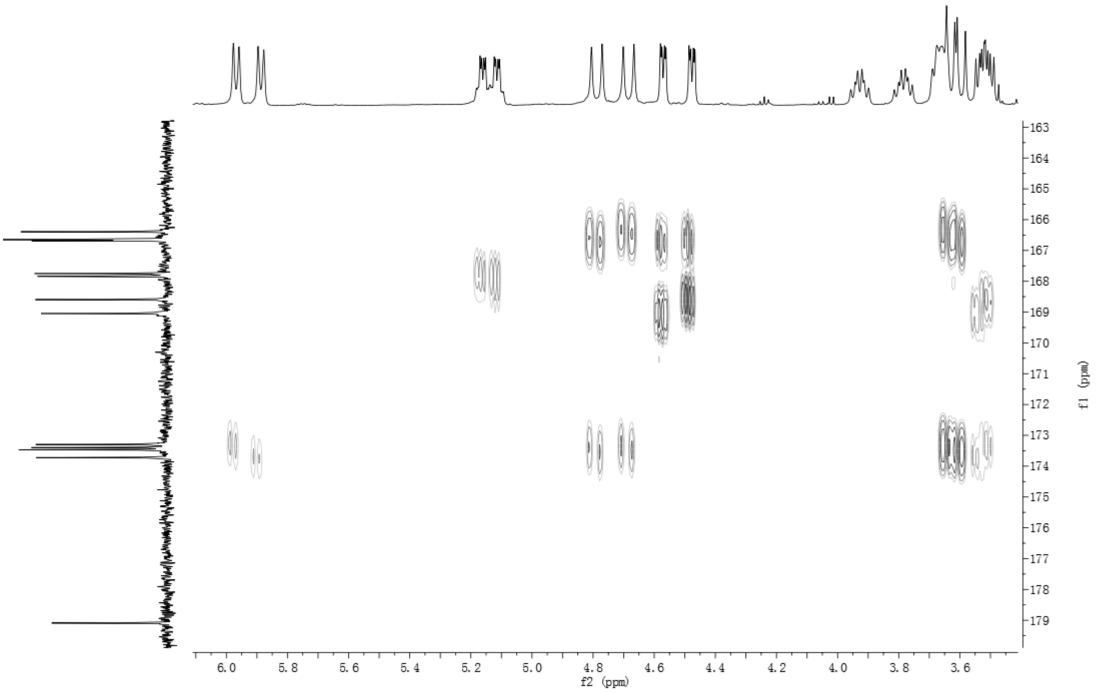

Figure S31. HMBC spectrum of actinomycin D (**1**, in $CDCl_3$-*d*).

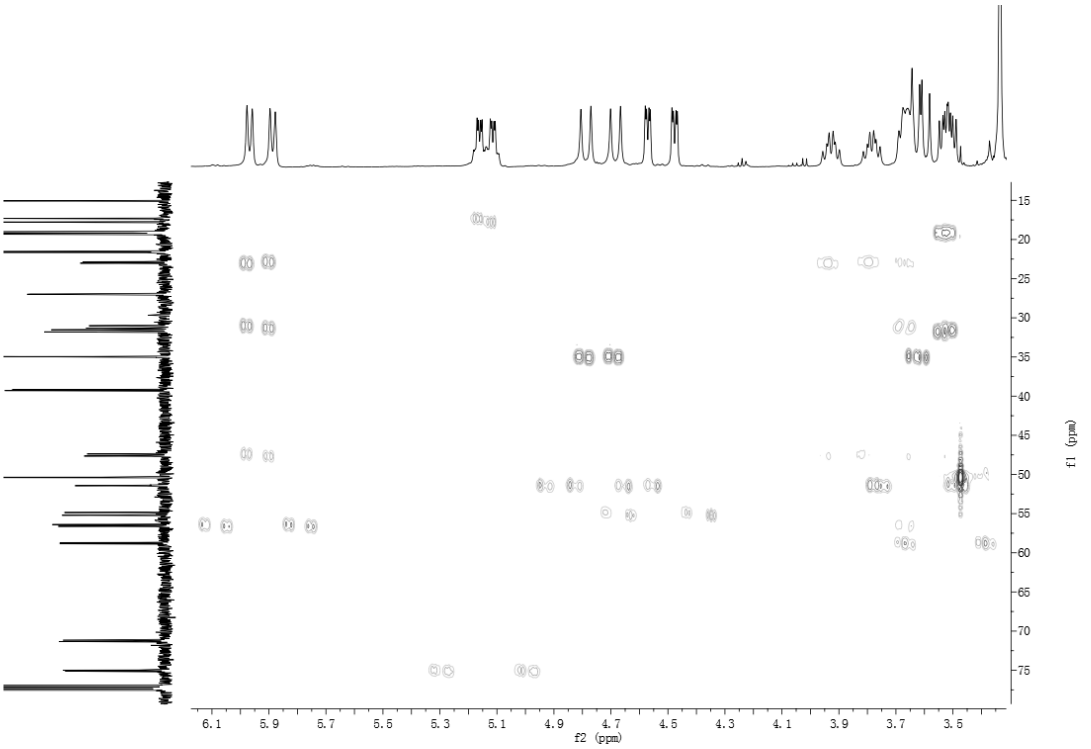

Figure S32. HMBC spectrum of actinomycin D (**1**, in $CDCl_3$-*d*).

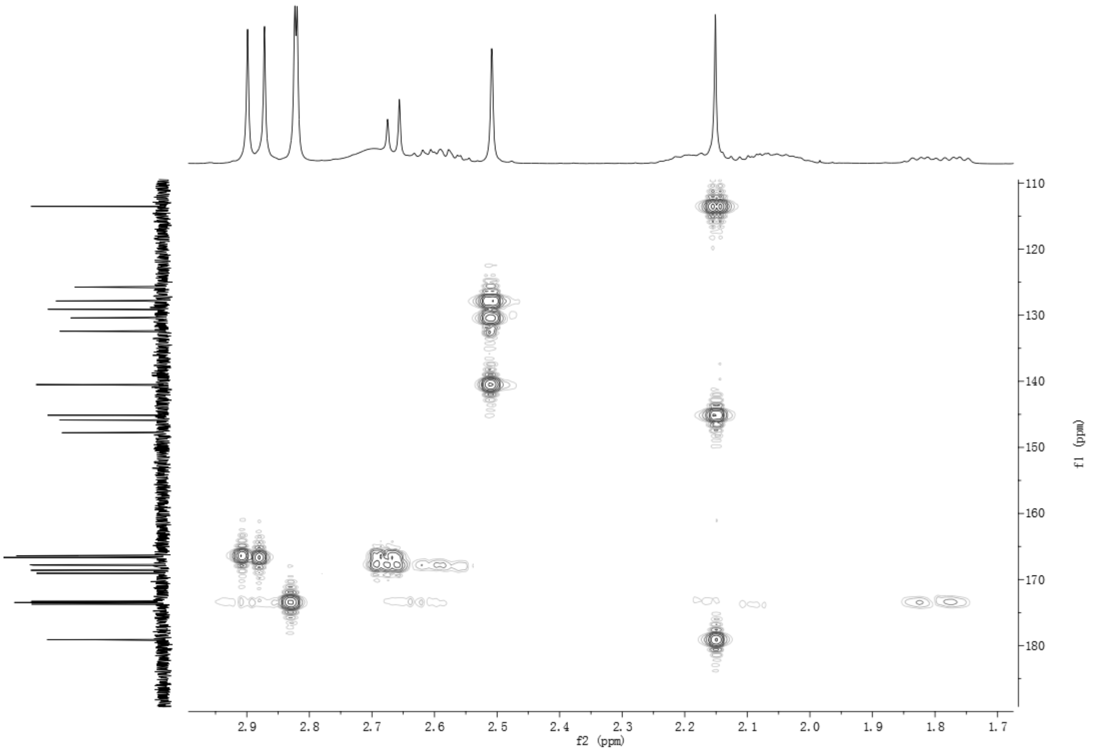

Figure S33. HMBC spectrum of actinomycin D (**1**, in CDCl3-*d*).

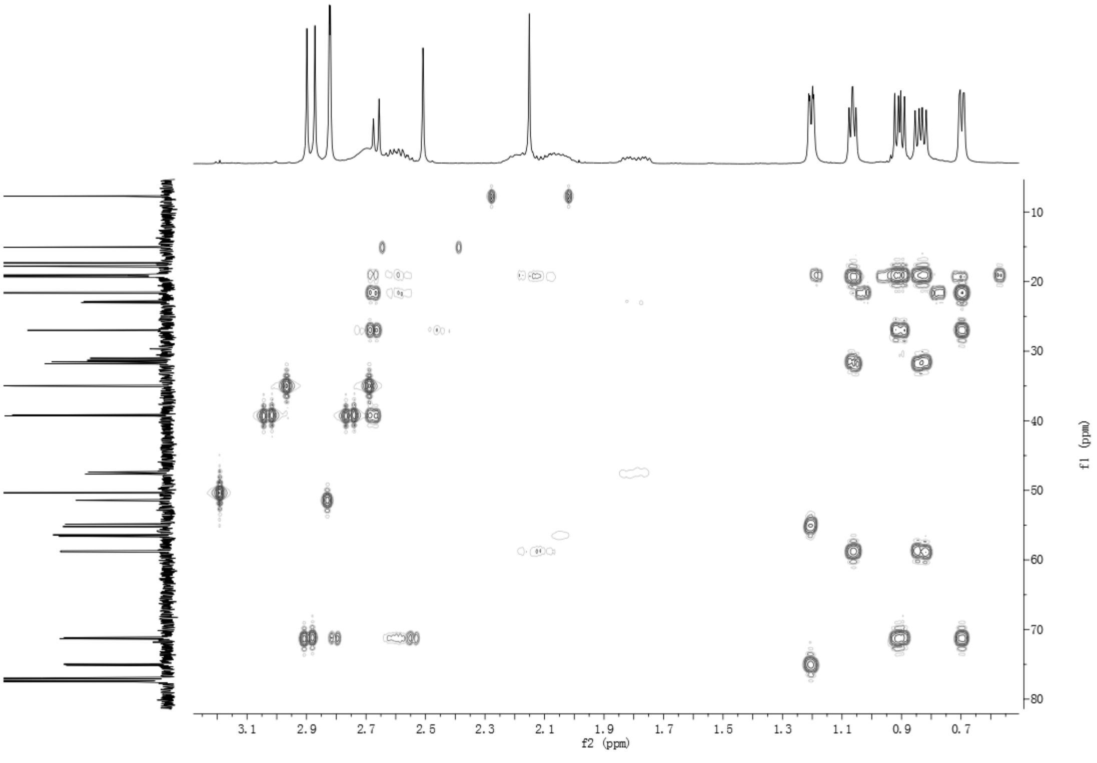

Figure S34. HMBC spectrum of actinomycin D (**1**, in CDCl3-*d*).

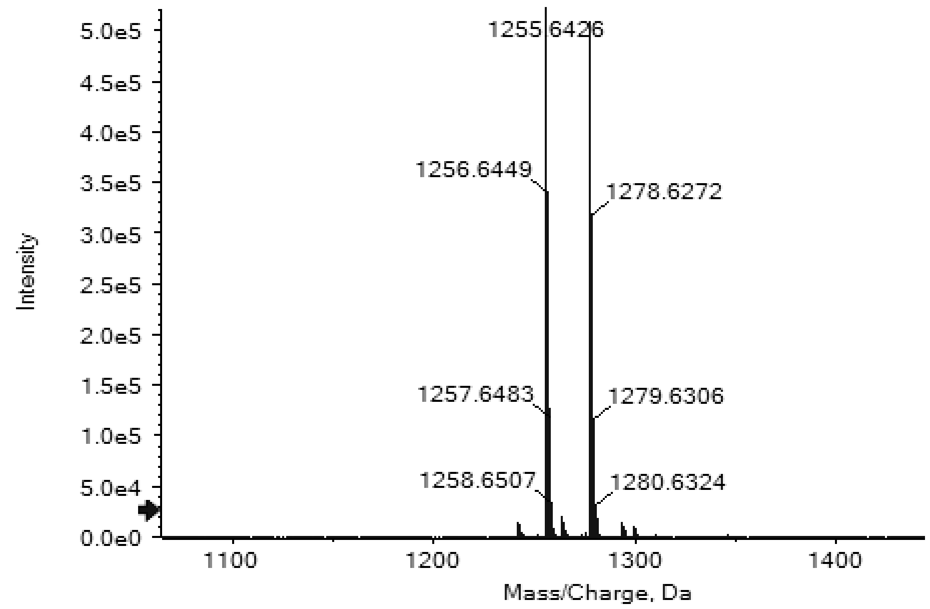

Figure S35. HRESIMS of actinomycin D (**1**, in $CDCl_3$-*d*).

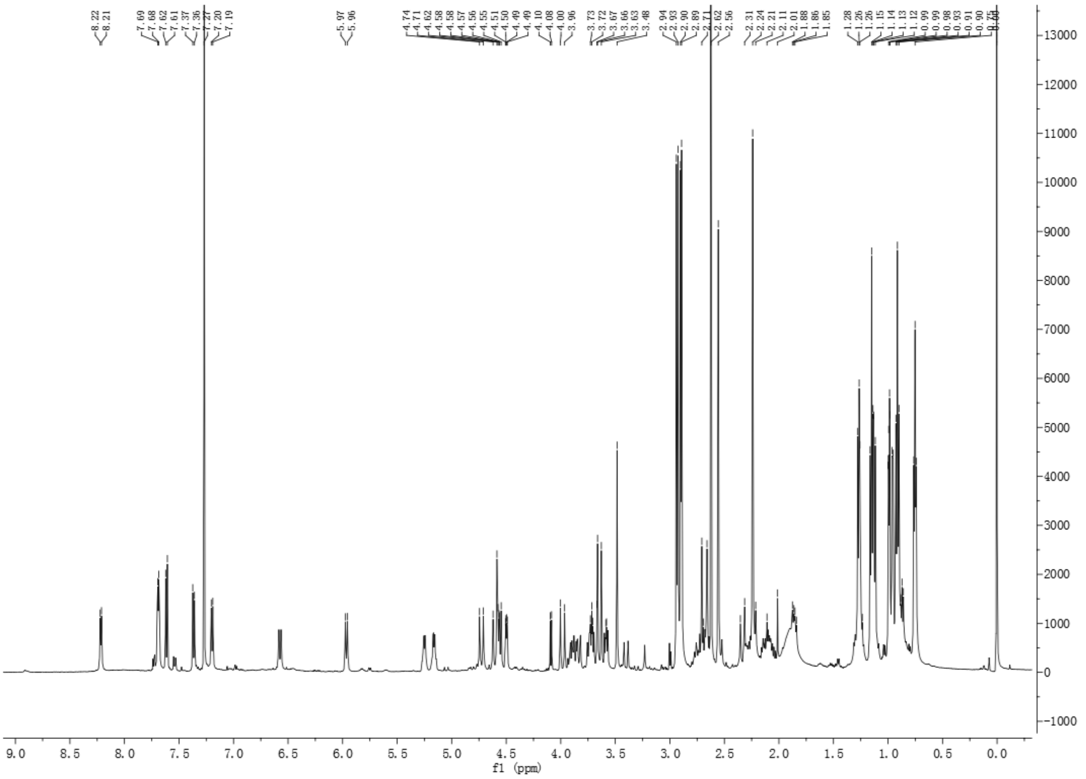

Figure S36. ^{1}H NMR spectrum of actinomycin V (**2**, in $CDCl_3$-*d*).

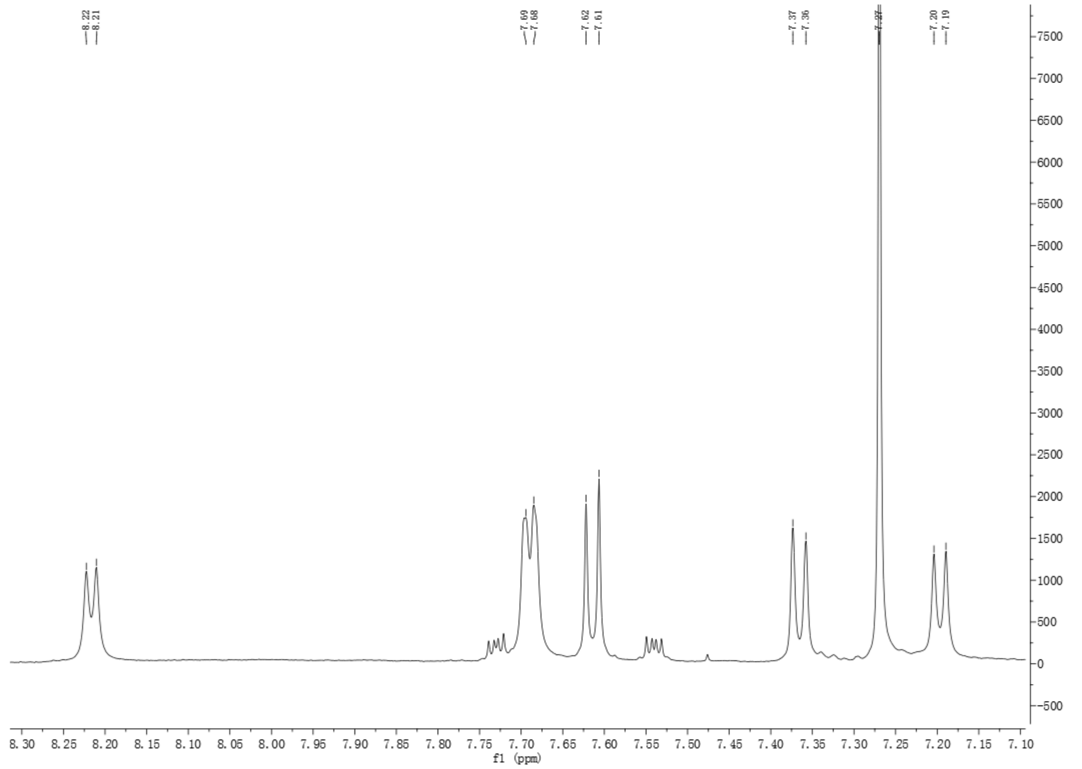

Figure S37. ^{1}H NMR spectrum of actinomycin V (**2**, in $CDCl_3$-*d*).

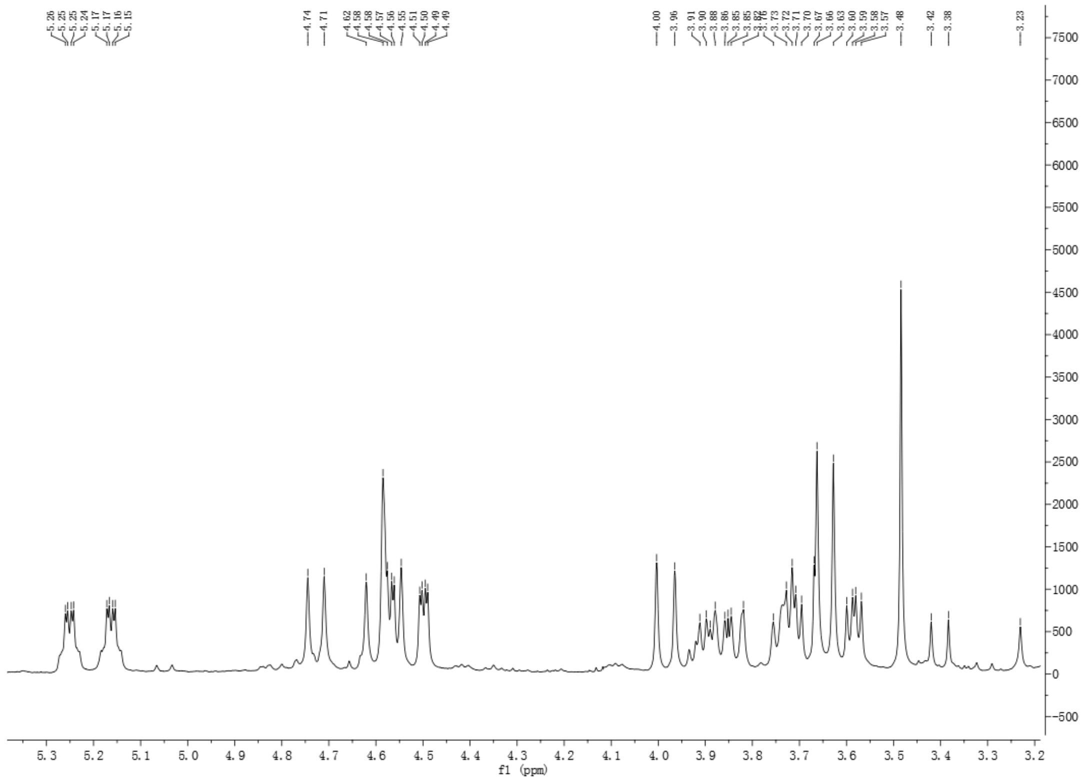

Figure S38. ^{1}H NMR spectrum of actinomycin V (**2**, in $CDCl_3$-*d*).

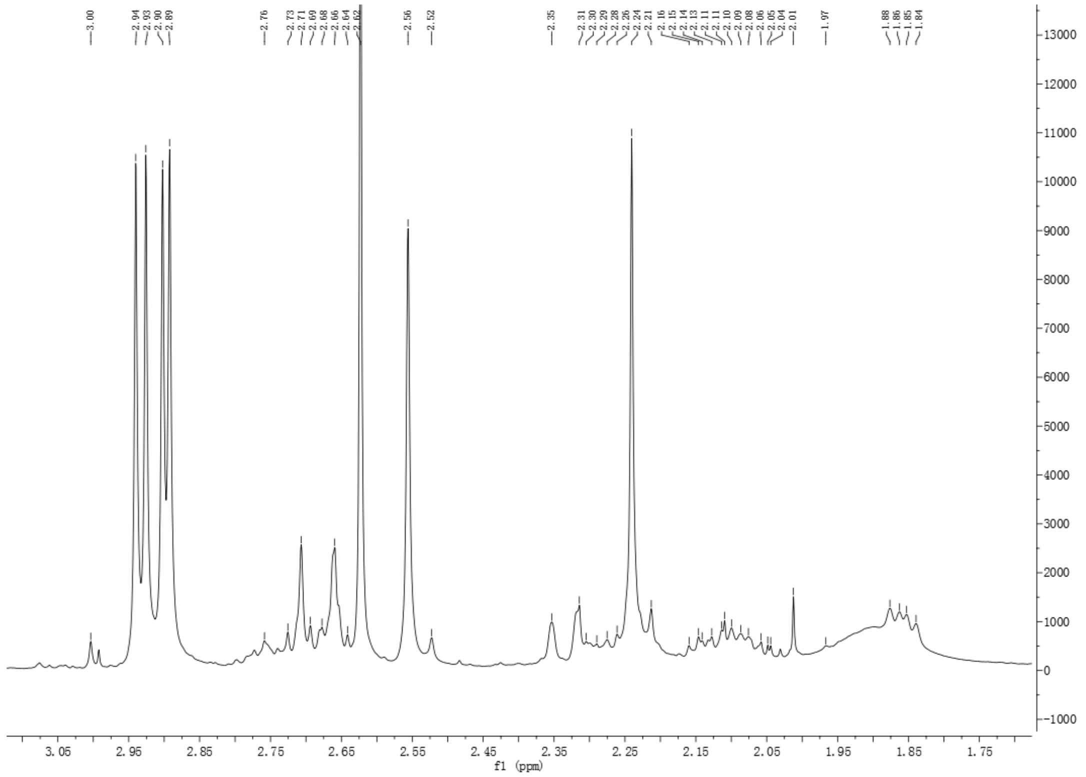

Figure S39. ^{1}H NMR spectrum of actinomycin V (**2**, in $CDCl_3$-*d*).

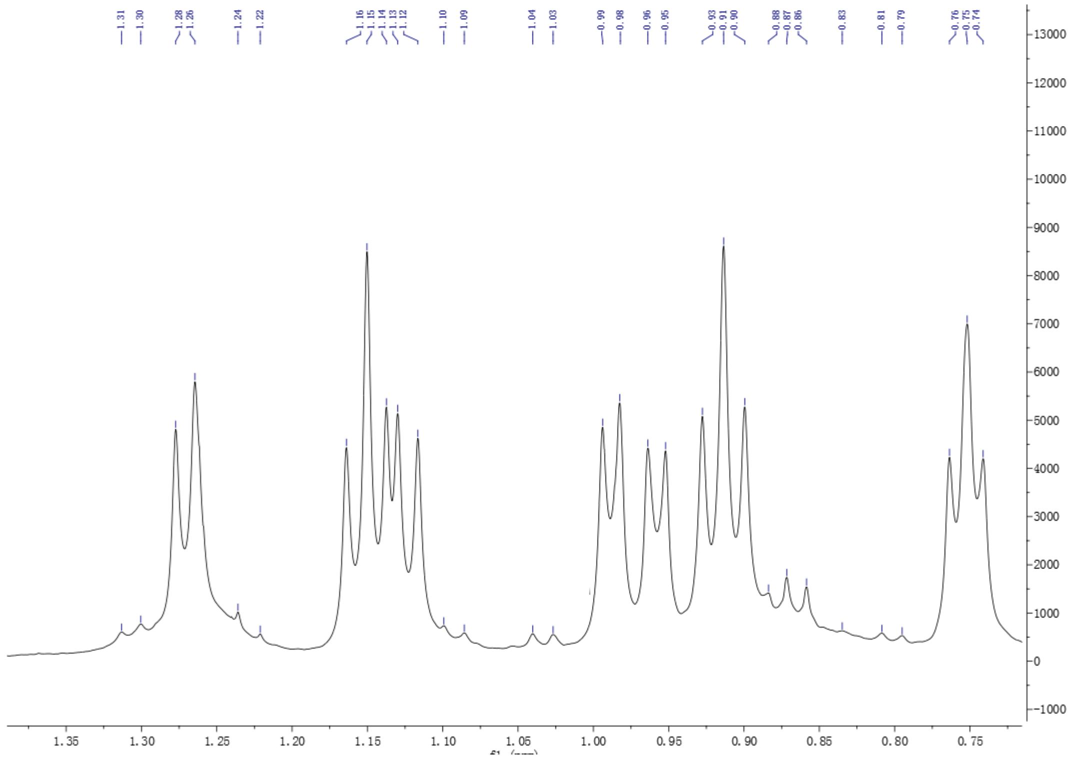

Figure S40. ^{1}H NMR spectrum of actinomycin V (**2**, in $CDCl_3$-*d*).

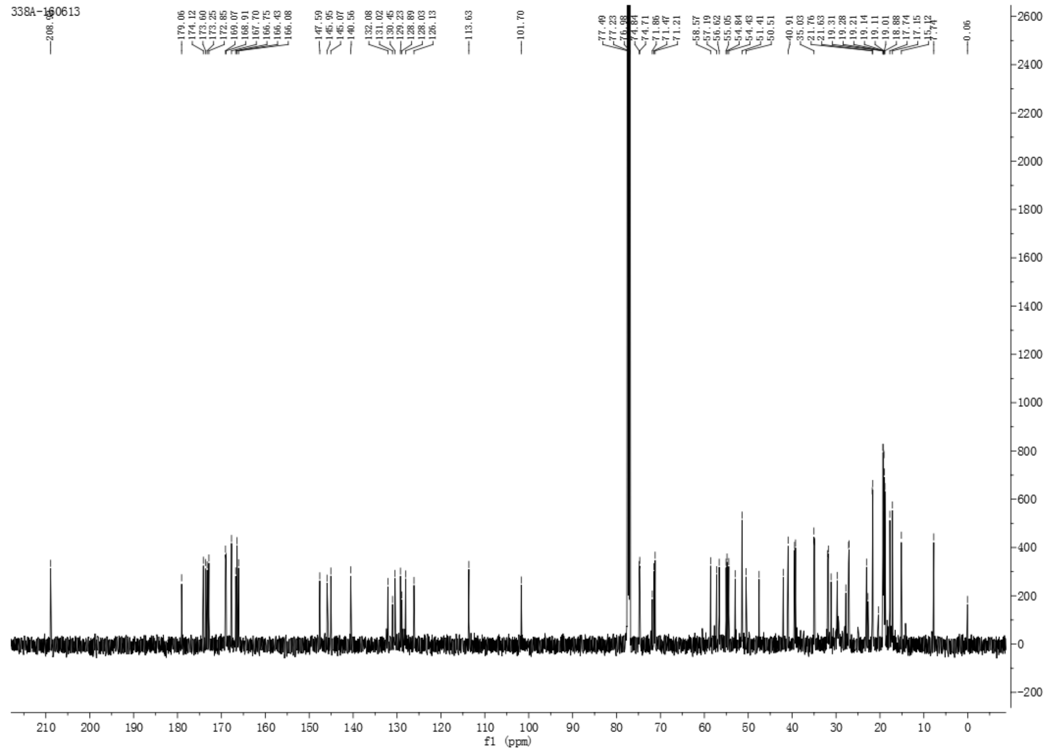

Figure S41. ^{13}C NMR spectrum of actinomycin V (**2**, in $CDCl_3$-*d*).

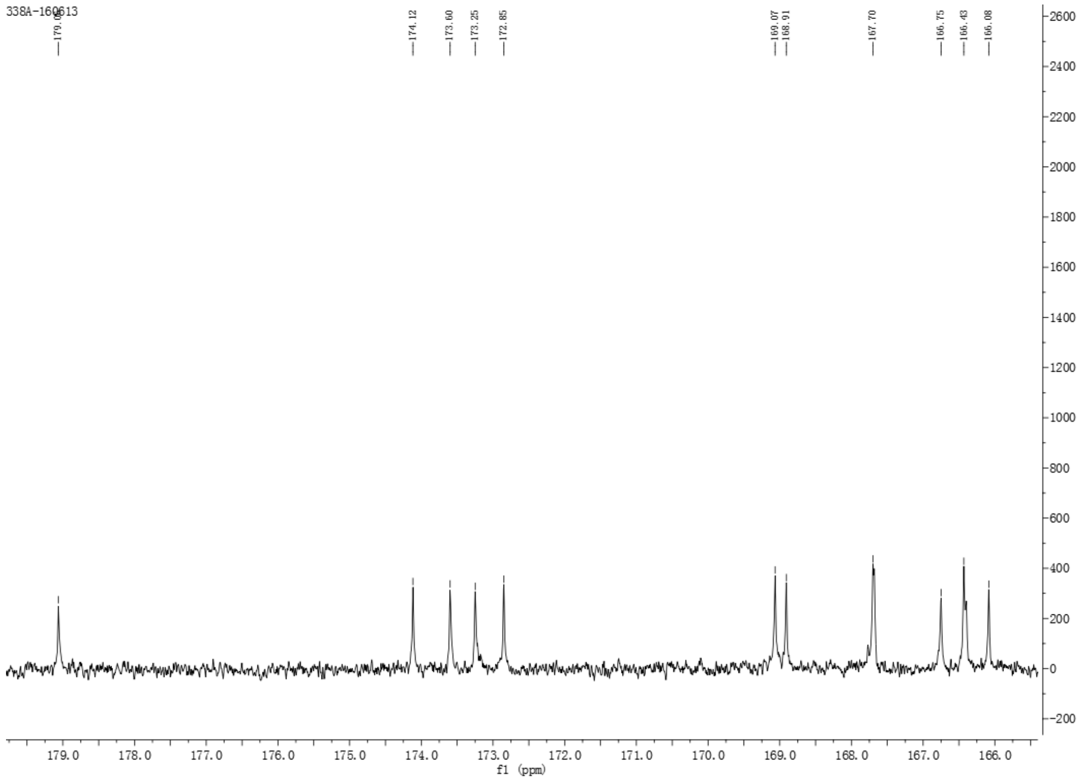

Figure S42. ^{13}C NMR spectrum of actinomycin V (**2**, in $CDCl_3$-*d*).

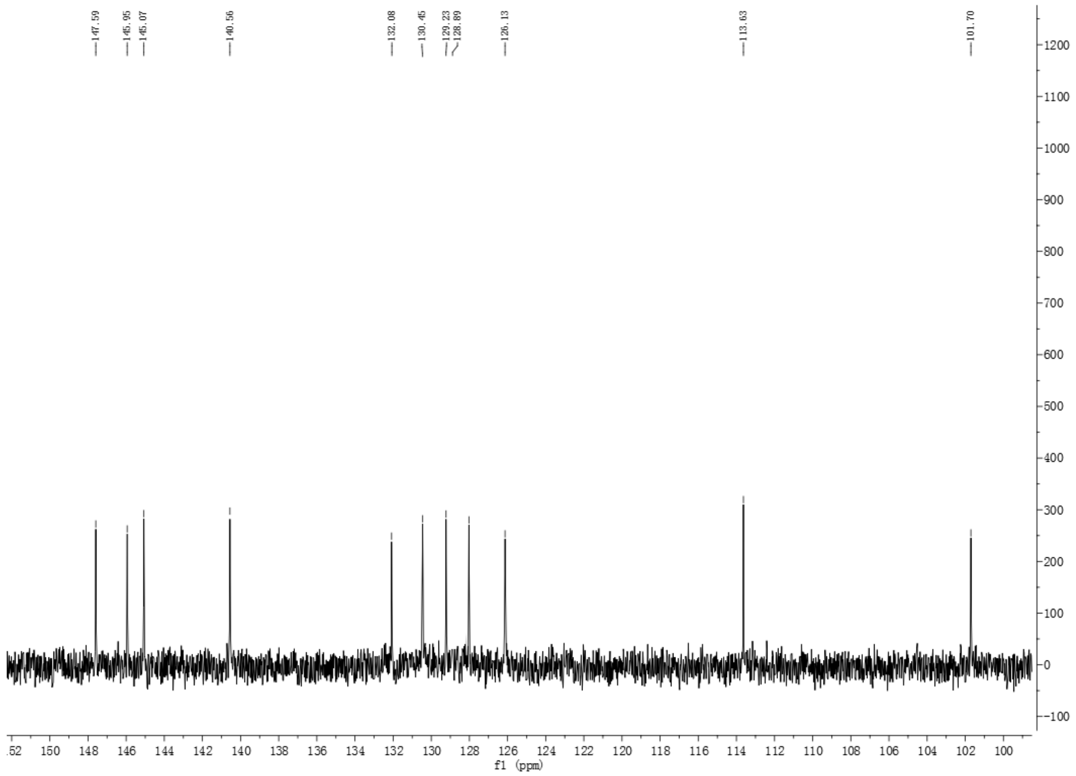

Figure S43. ^{13}C NMR spectrum of actinomycin V (**2**, in CDCl3-*d*).

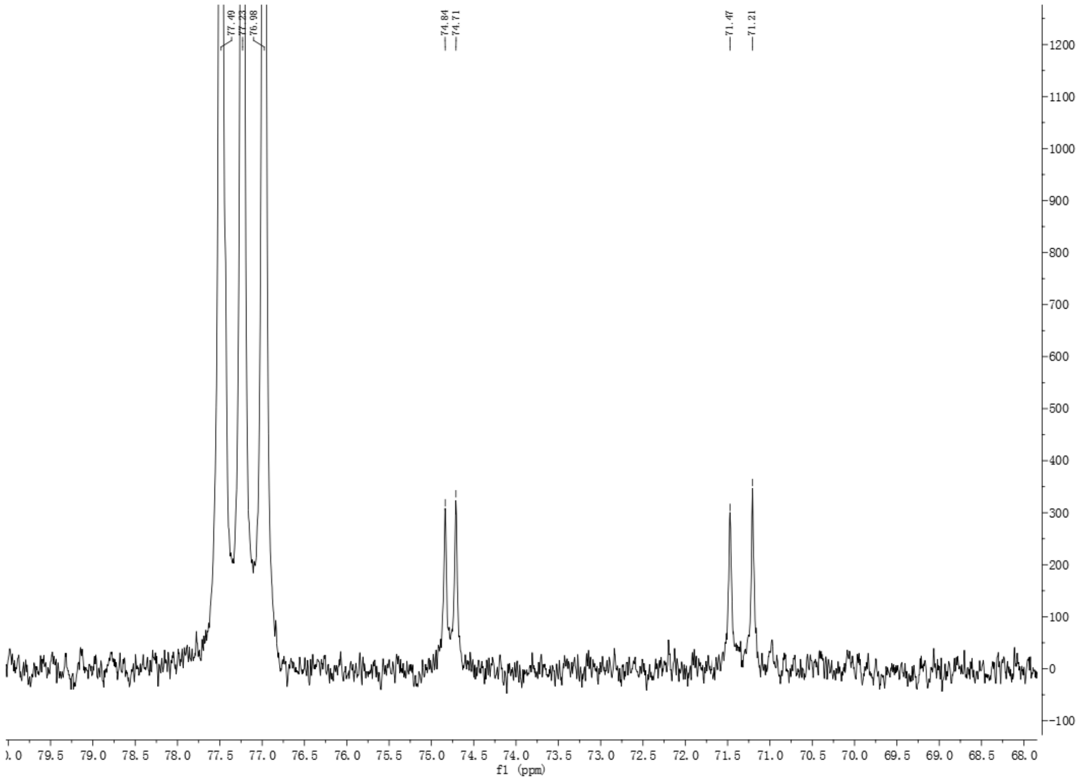

Figure S44. ^{13}C NMR spectrum of actinomycin V (**2**, in CDCl3-*d*).

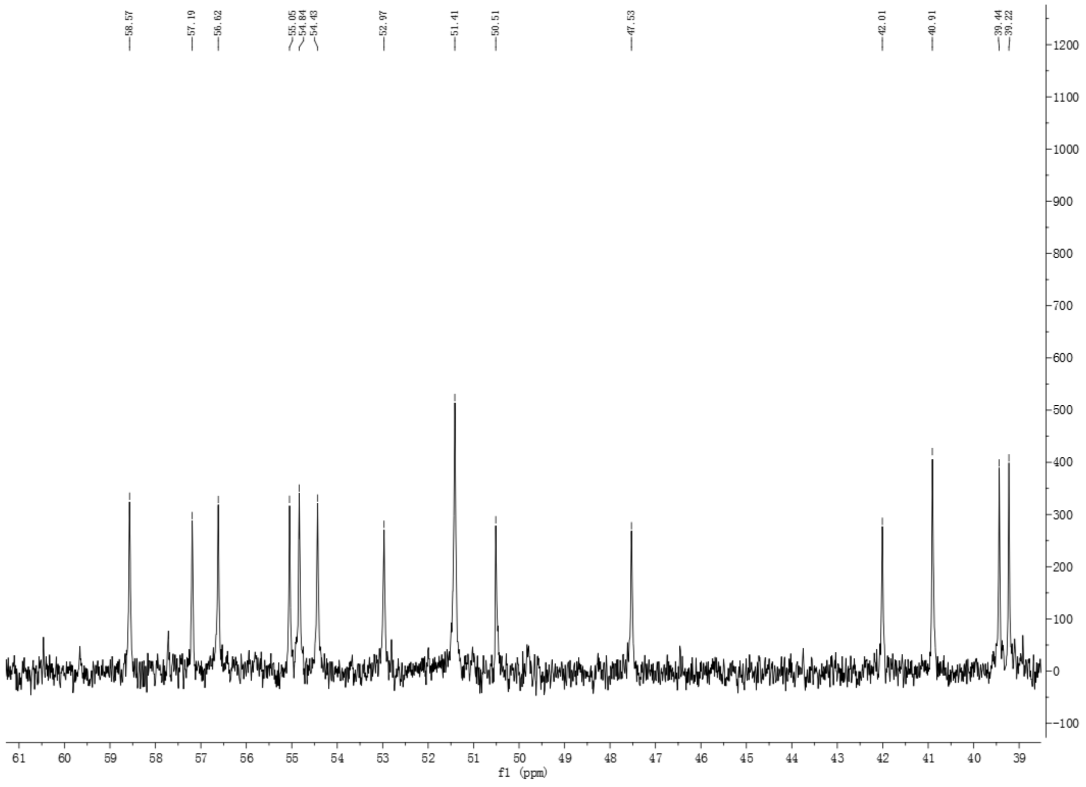

Figure S45. ^{13}C NMR spectrum of actinomycin V (**2**, in CDCl3-*d*).

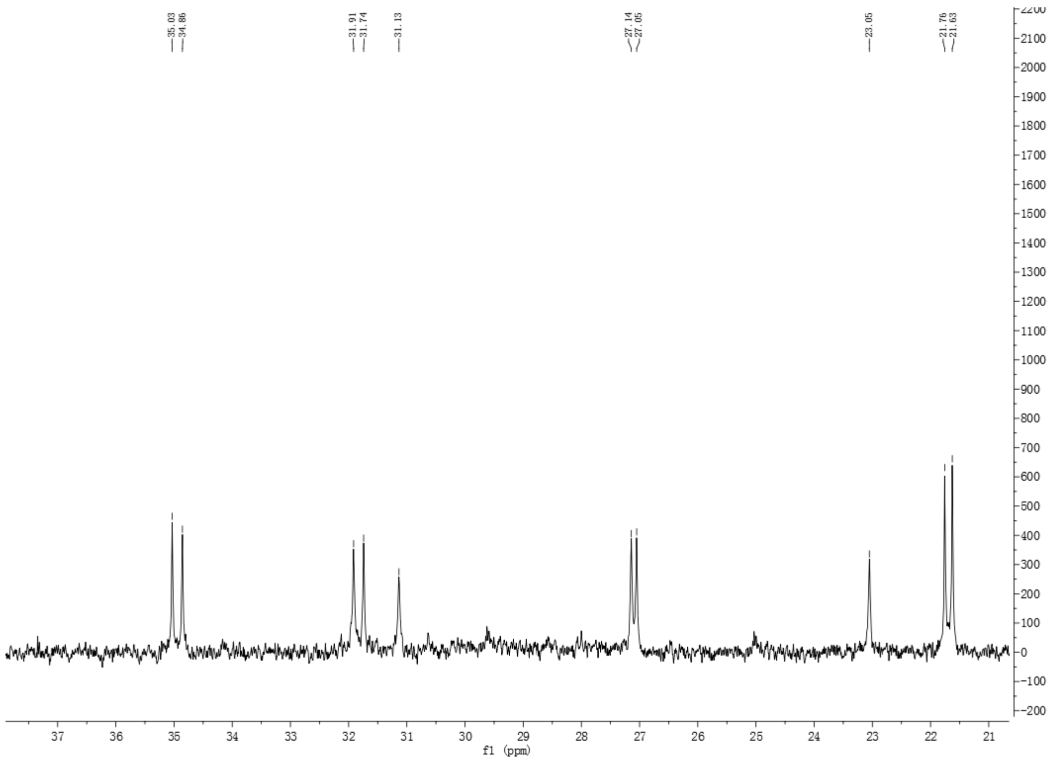

Figure S46. ^{13}C NMR spectrum of actinomycin V (**2**, in CDCl3-*d*).

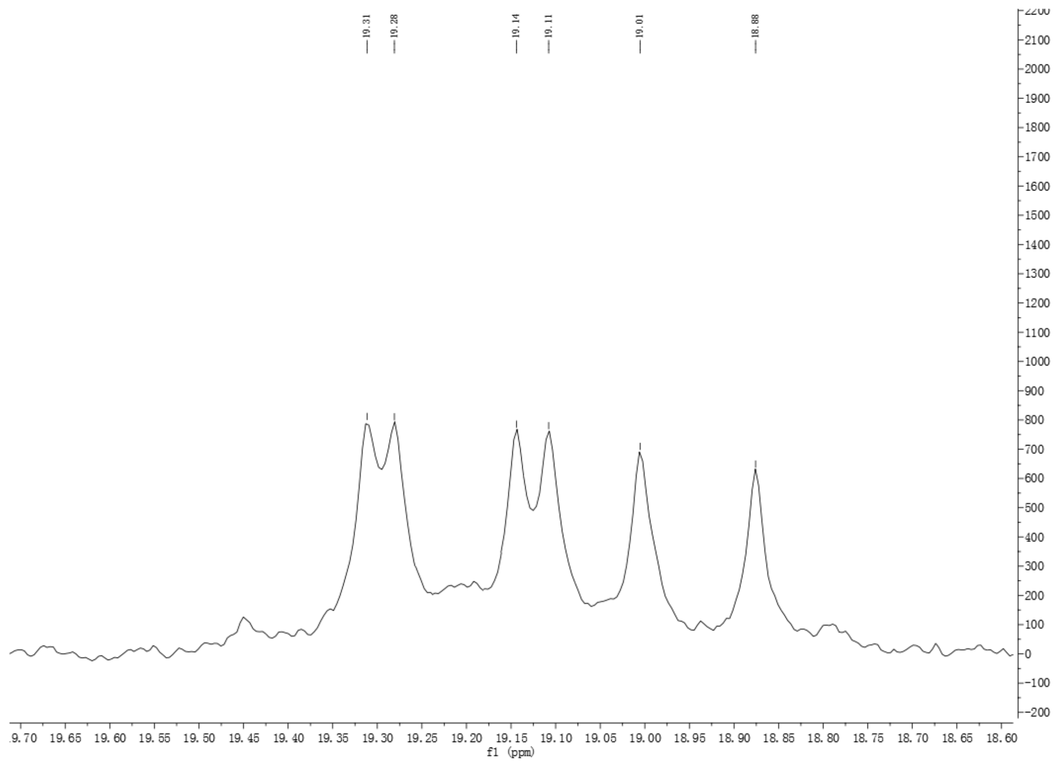

Figure S47. ^{13}C NMR spectrum of actinomycin V (**2**, in $CDCl_3$-*d*).

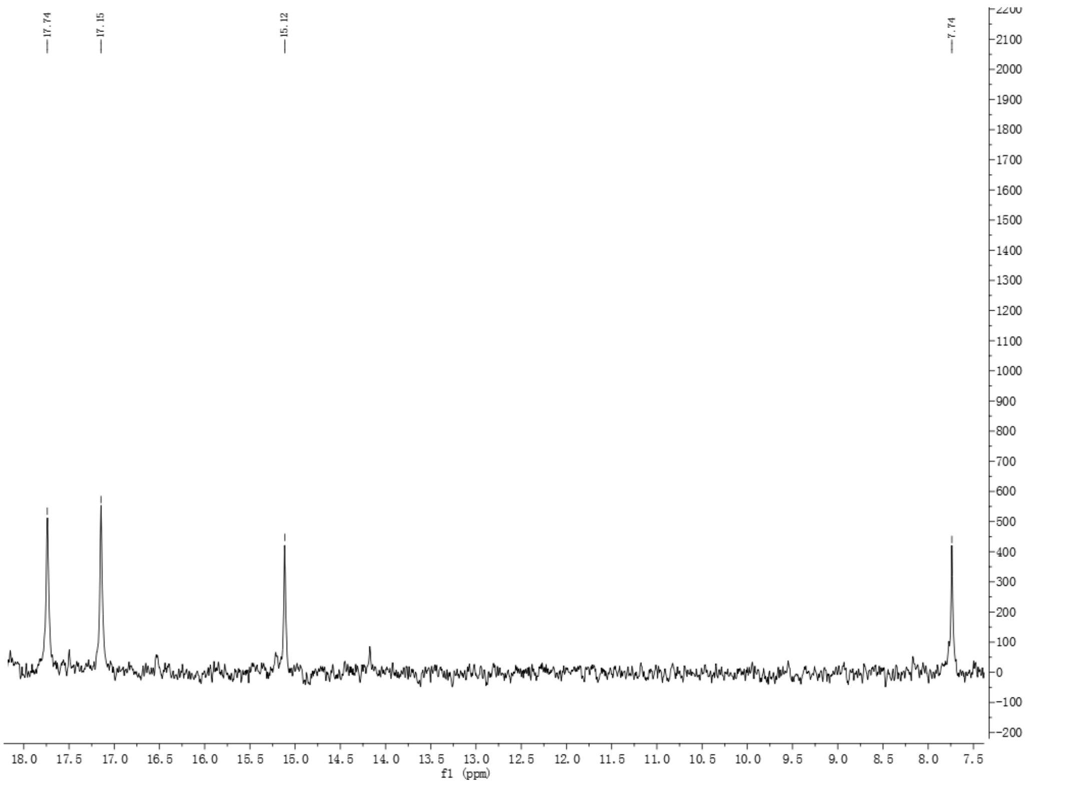

Figure S48. ^{13}C NMR spectrum of actinomycin V (**2**, in $CDCl_3$-*d*).

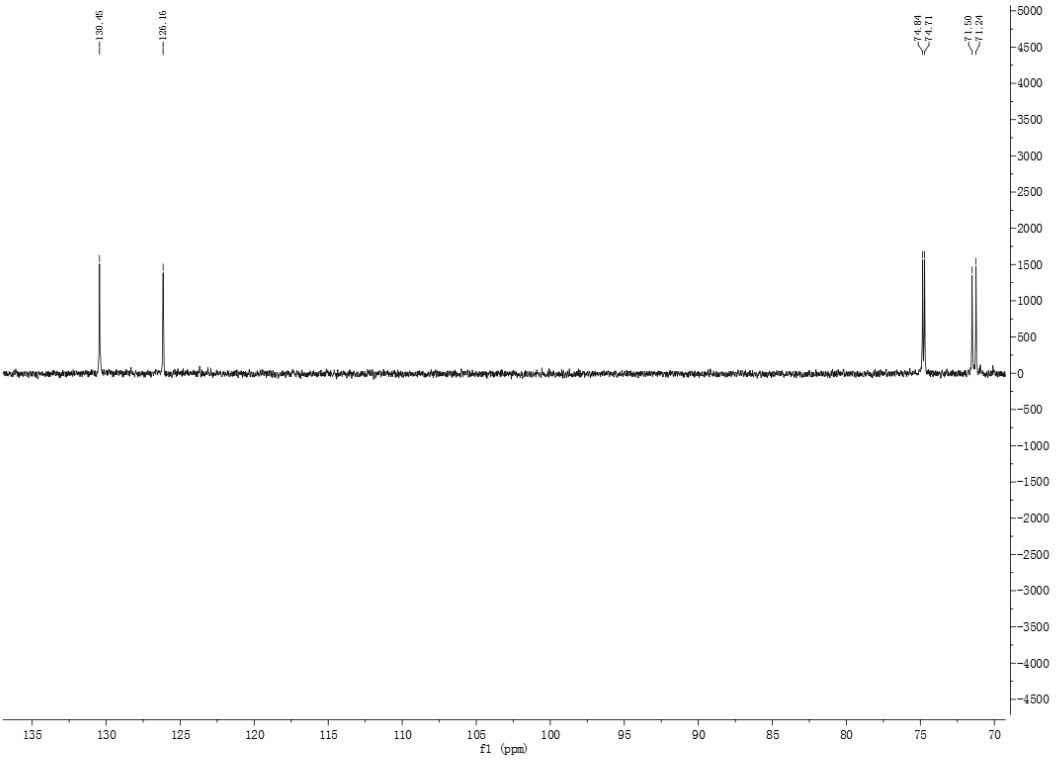

Figure S49. DEPT spectrum of actinomycin V (**2**, in CDCl3-*d*).

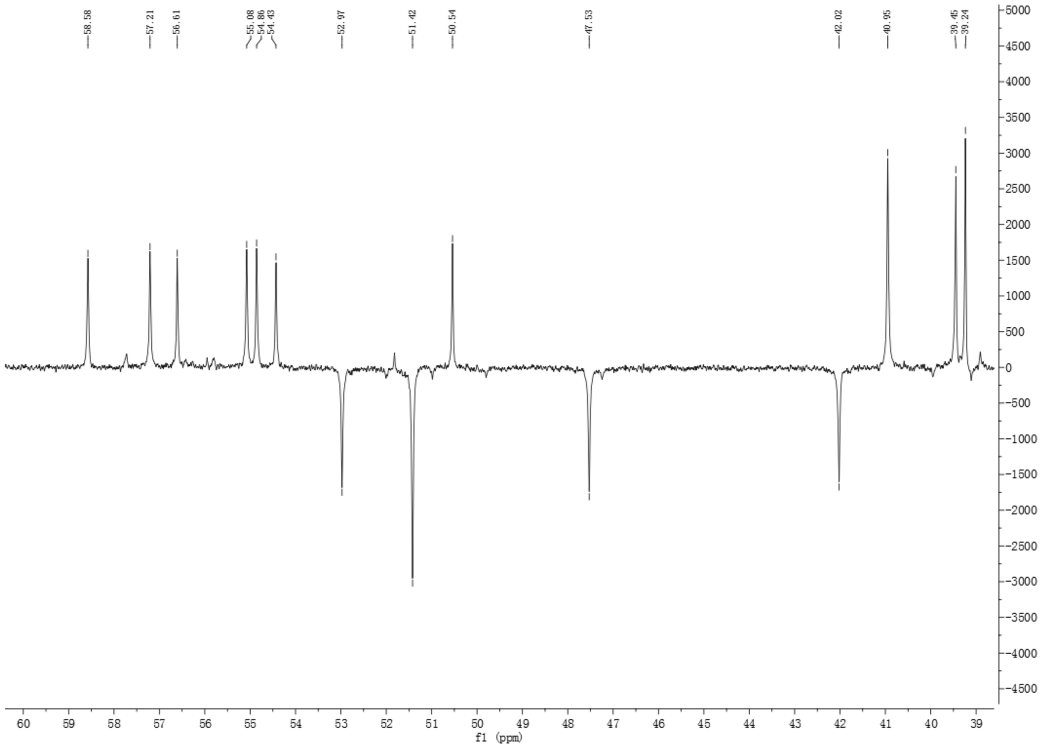

Figure S50. DEPT spectrum of actinomycin V (**2**, in CDCl3-*d*).

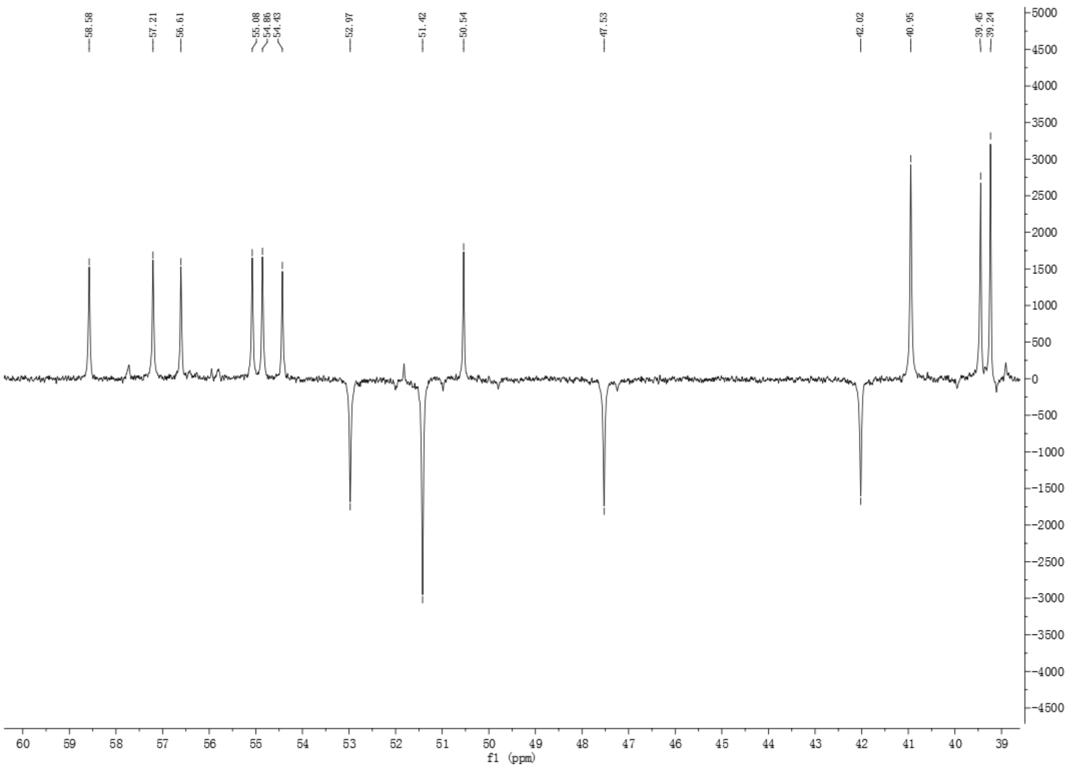

Figure S51. DEPT spectrum of actinomycin V (**2**, in $CDCl_3$-*d*).

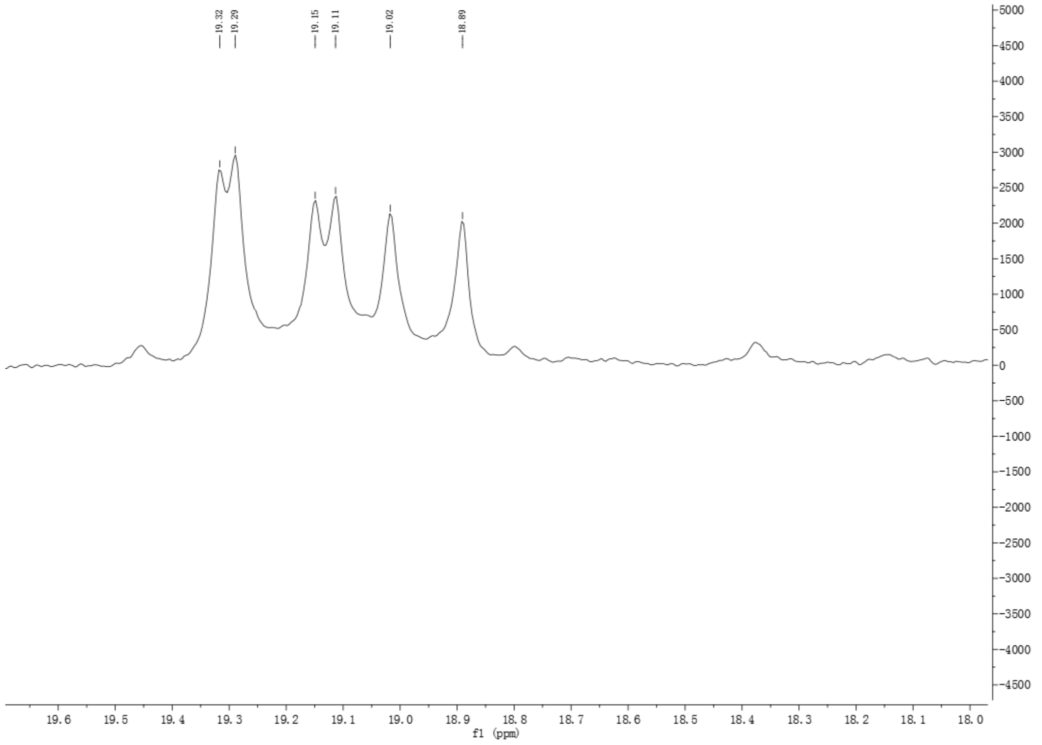

Figure S52. DEPT spectrum of actinomycin V (**2**, in $CDCl_3$-*d*).

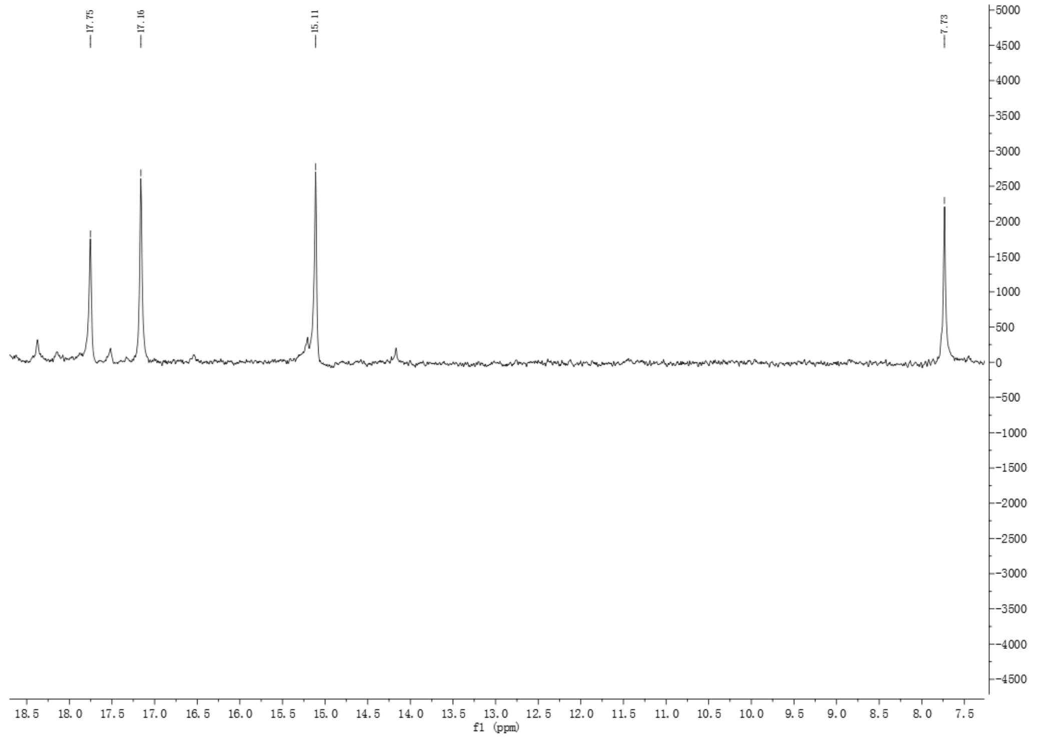

Figure S53. DEPT spectrum of actinomycin V (**2**, in $CDCl_3$-*d*).

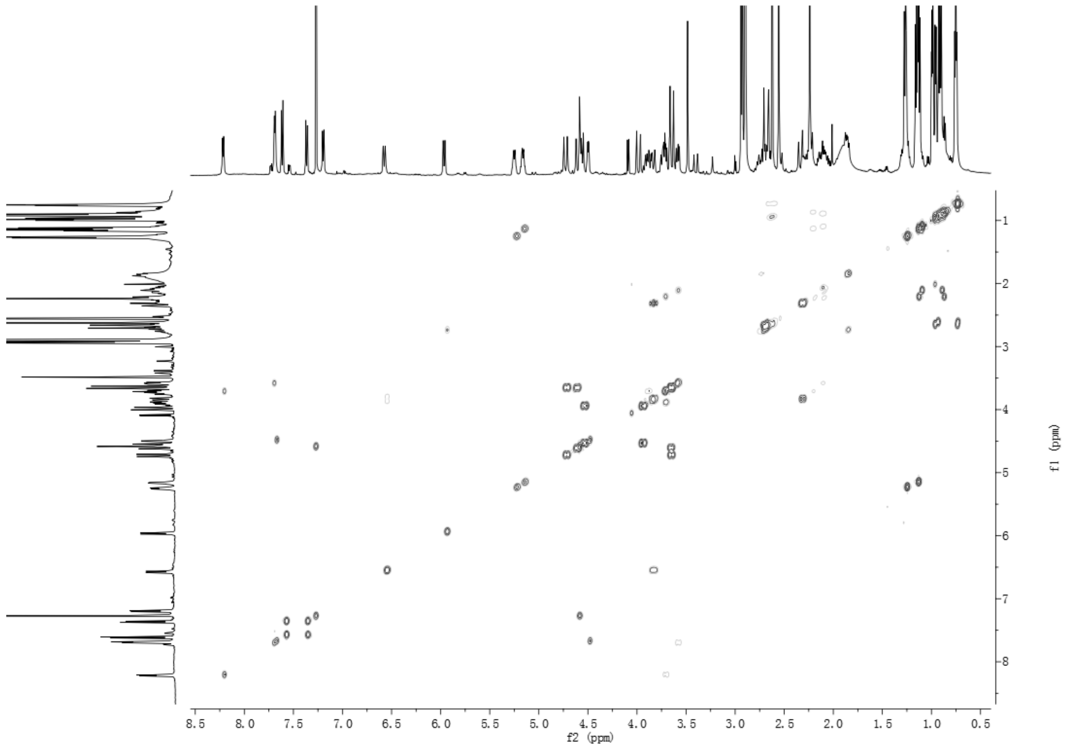

Figure S54. ^1H-^1H COSY spectrum of actinomycin V (**2**, in $CDCl_3$-*d*).

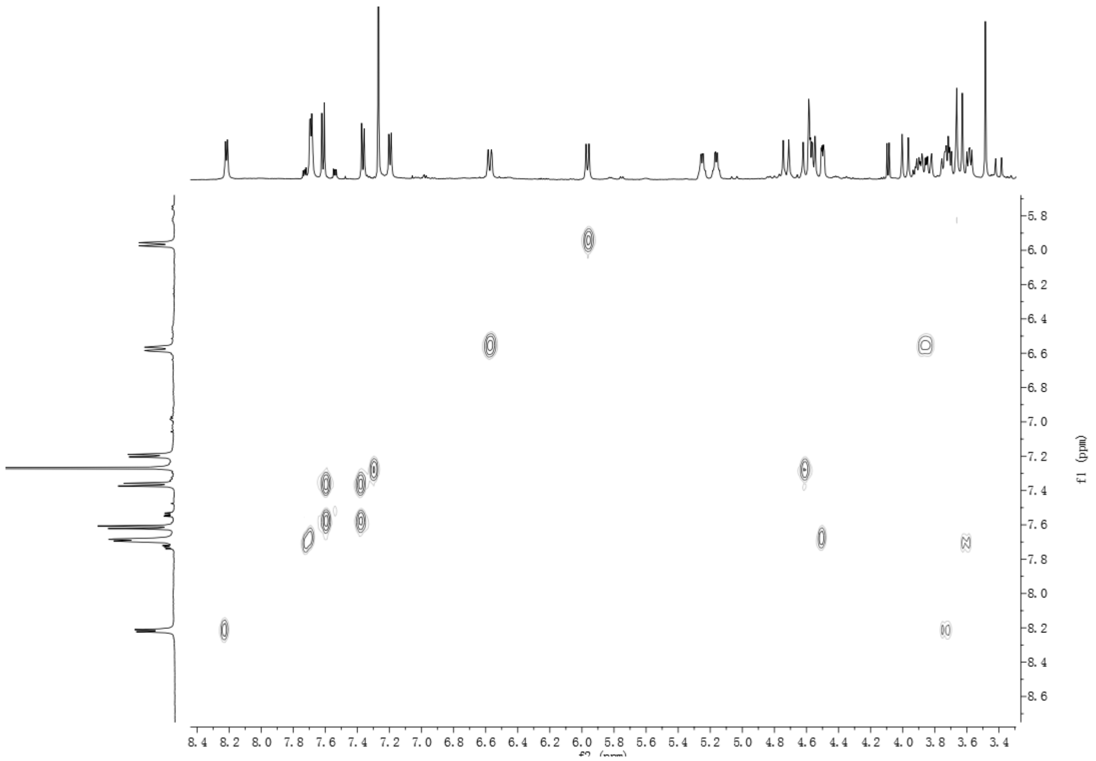

Figure S55. ^{1}H-^{1}H COSY spectrum of actinomycin V (**2**, in $CDCl_3$-*d*).

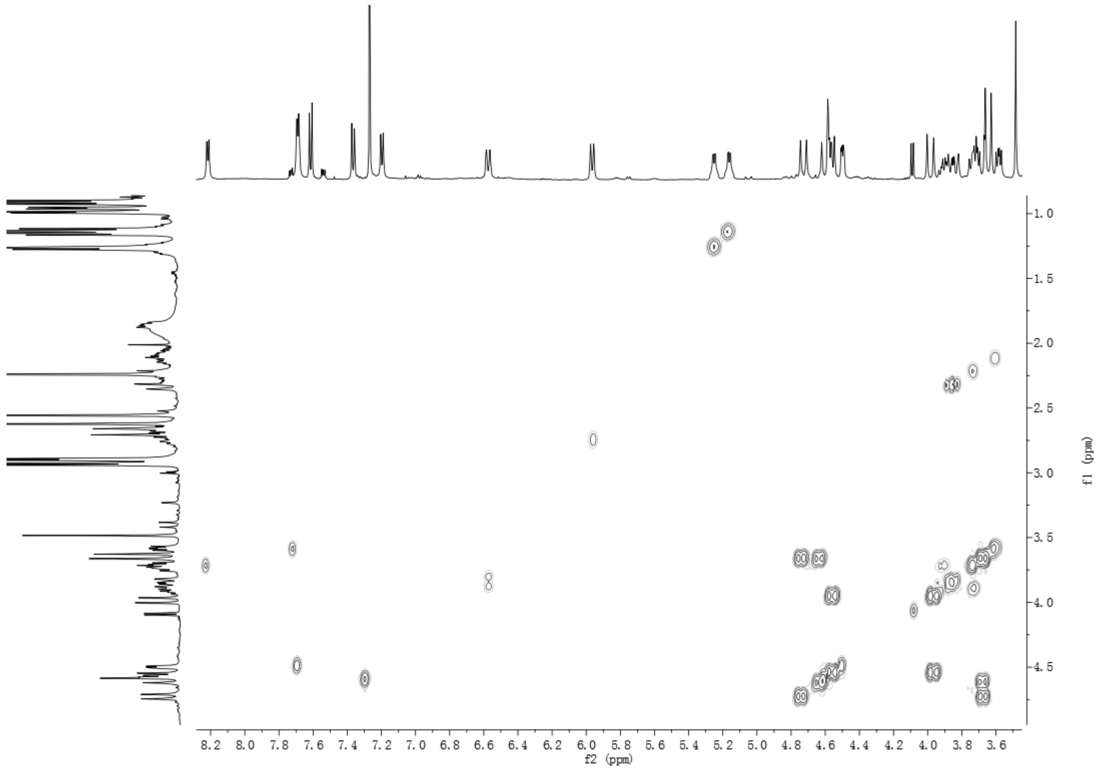

Figure S56. ^{1}H–^{1}H COSY spectrum of actinomycin V (**2**, in $CDCl_3$-*d*).

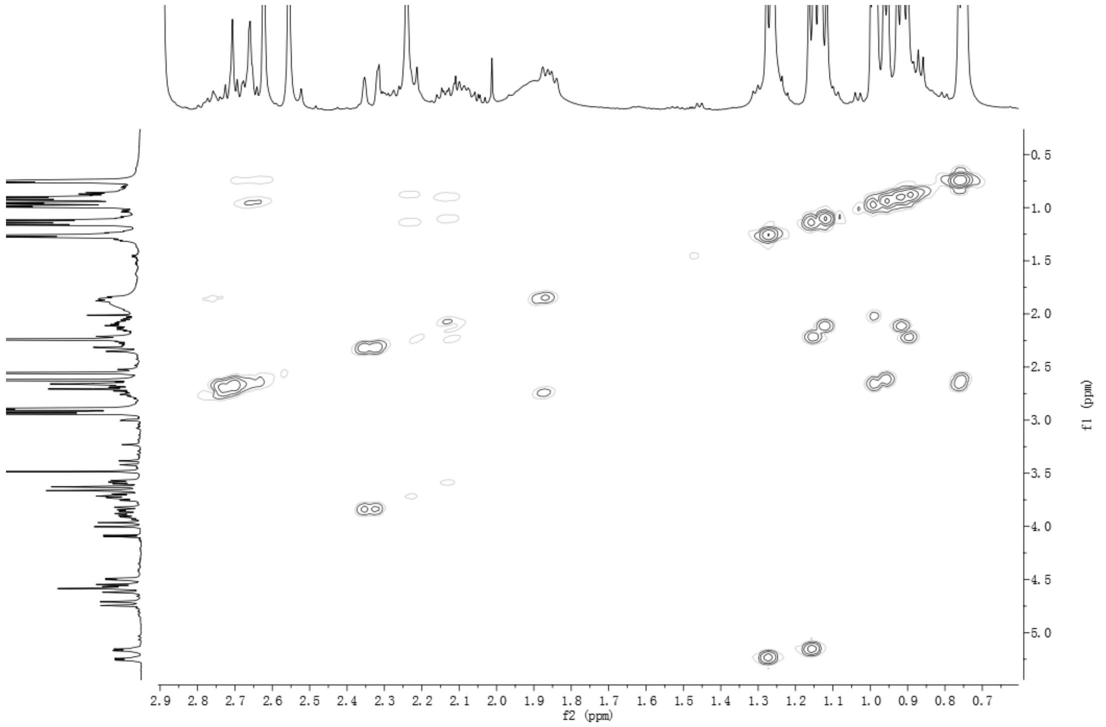

Figure S57. ^{1}H–^{1}H COSY spectrum of actinomycin V (**2**, in $CDCl_3$-*d*).

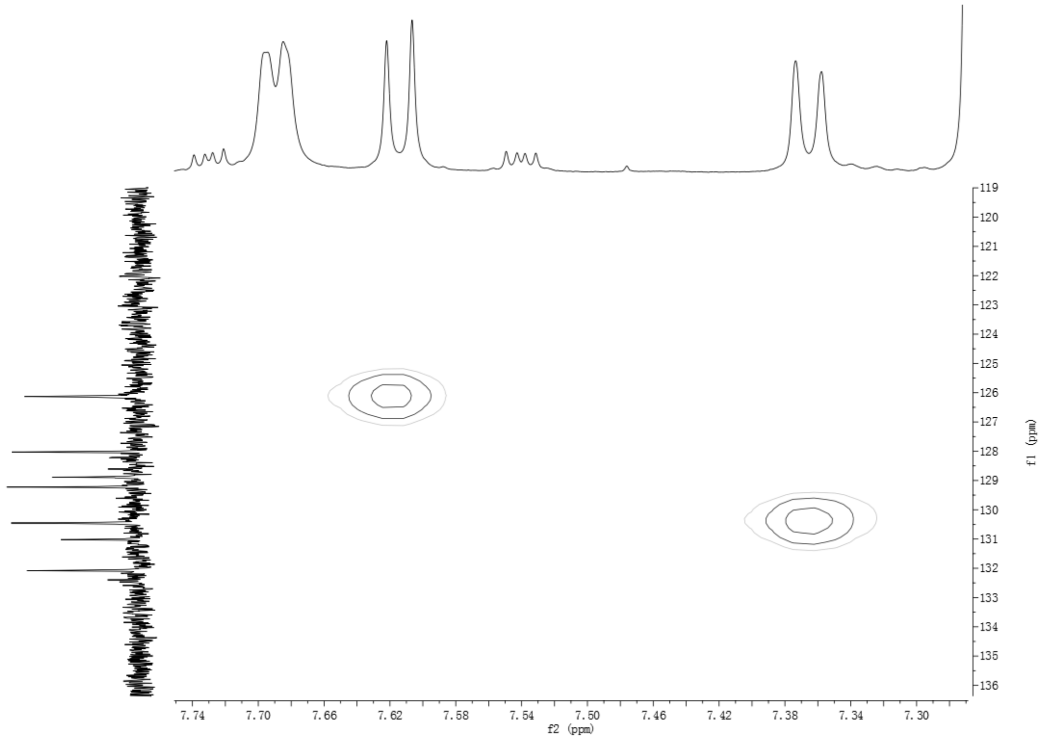

Figure S58. HSQC spectrum of actinomycin V (**2**, in $CDCl_3$-*d*).

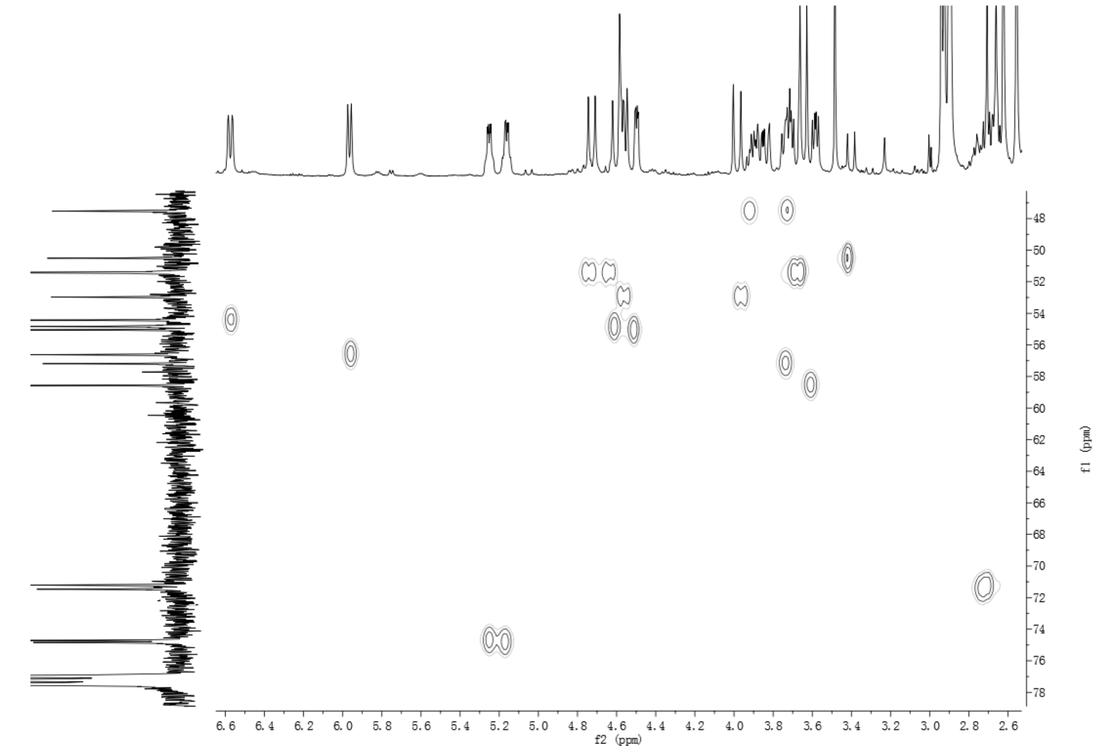

Figure S59. HSQC spectrum of actinomycin V (**2**, in CDCl3-*d*).

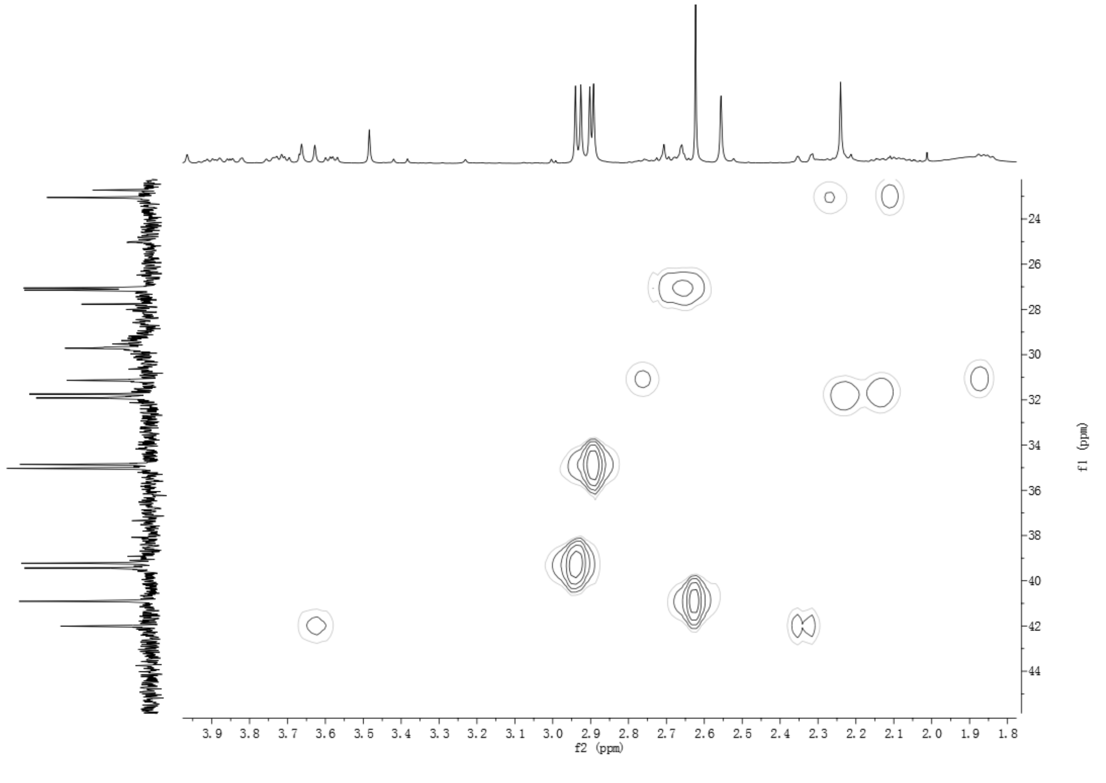

Figure S60. HSQC spectrum of actinomycin V (**2**, in CDCl3-*d*).

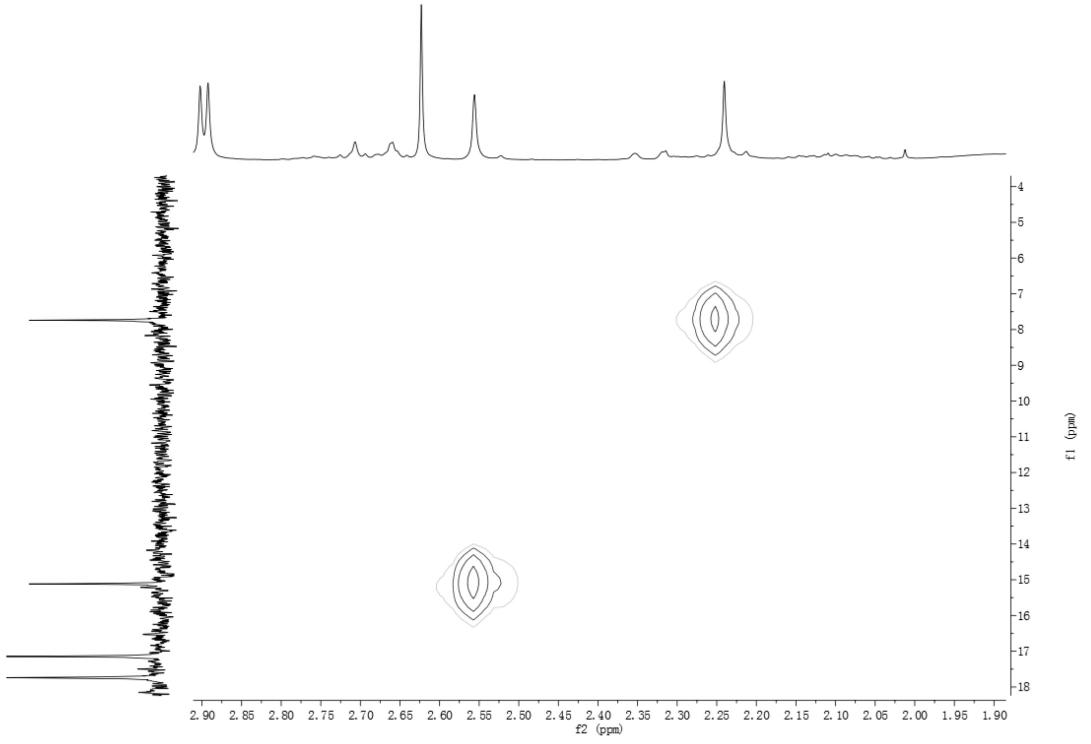

Figure S61. HSQC spectrum of actinomycin V (**2**, in $CDCl_3$-*d*).

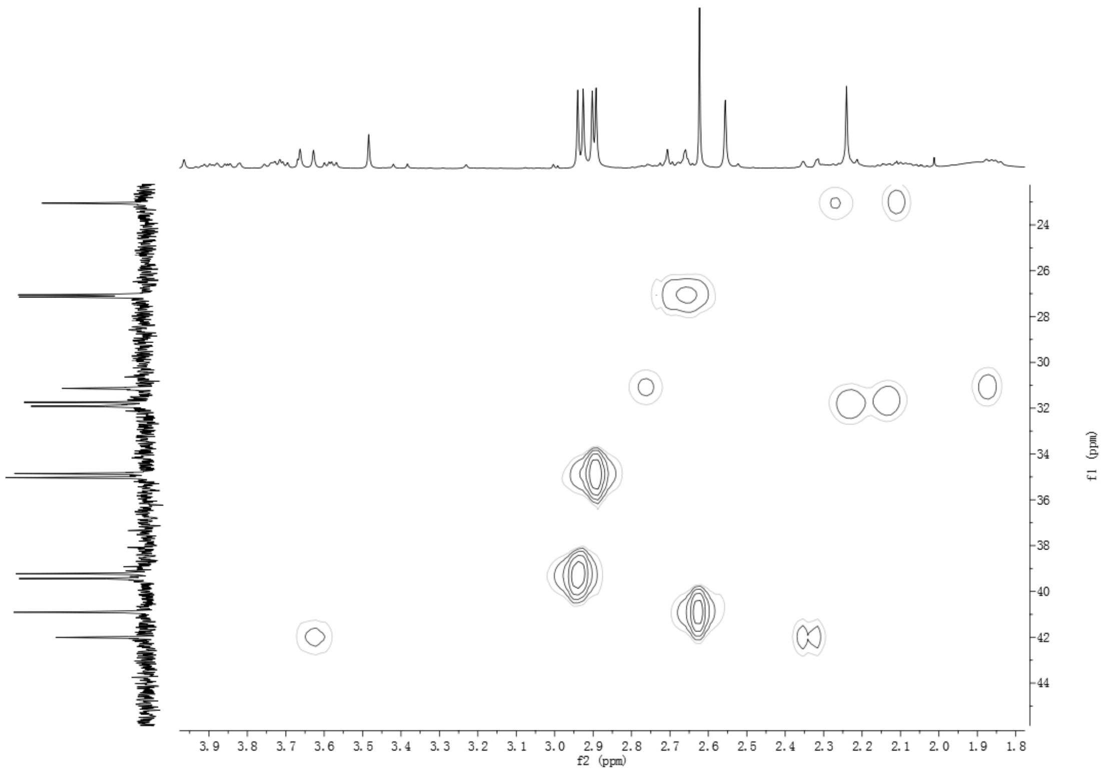

Figure S62. HSQC spectrum of actinomycin V (**2**, in $CDCl_3$-*d*).

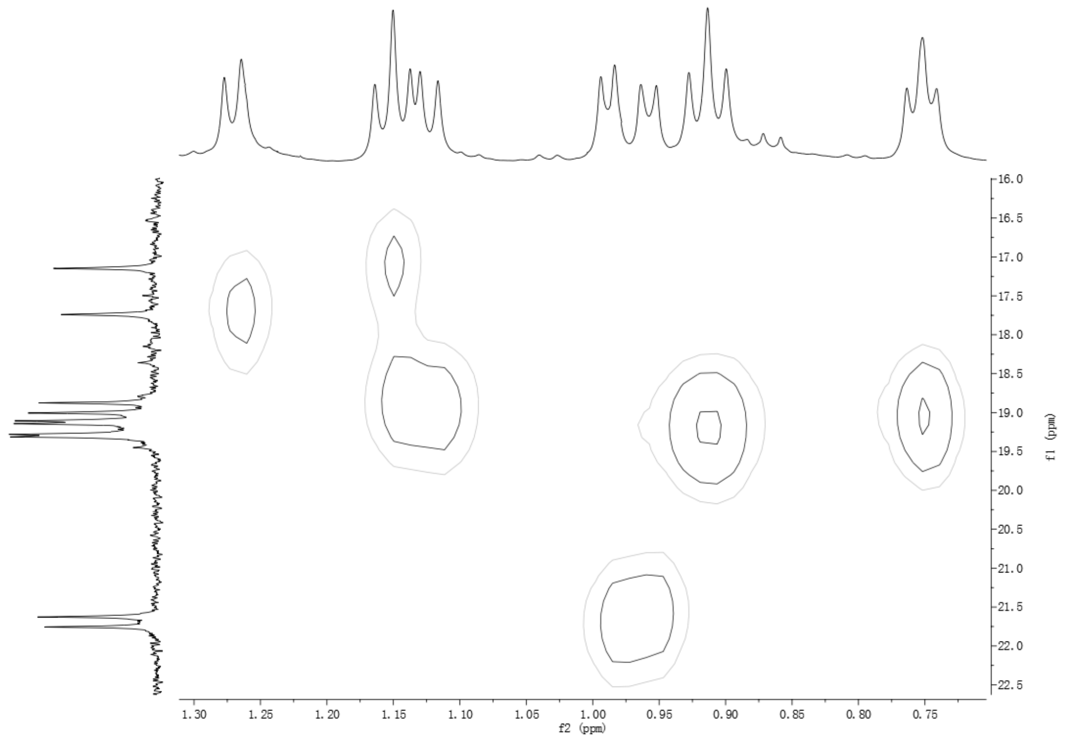

Figure S63. HSQC spectrum of actinomycin V (**2**, in CDCl3-*d*).

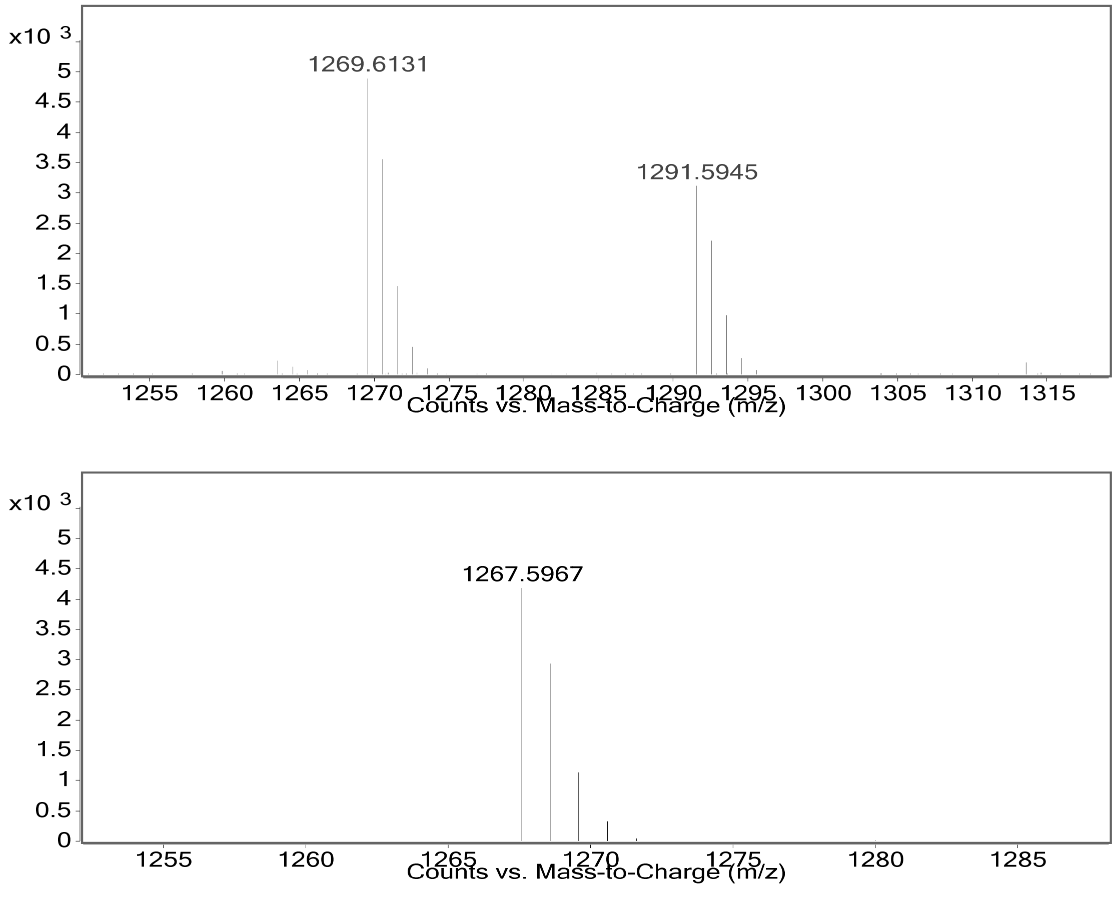

Figure S64. HRESIMS of actinomycin V (**2**, in CDCl3-*d*).

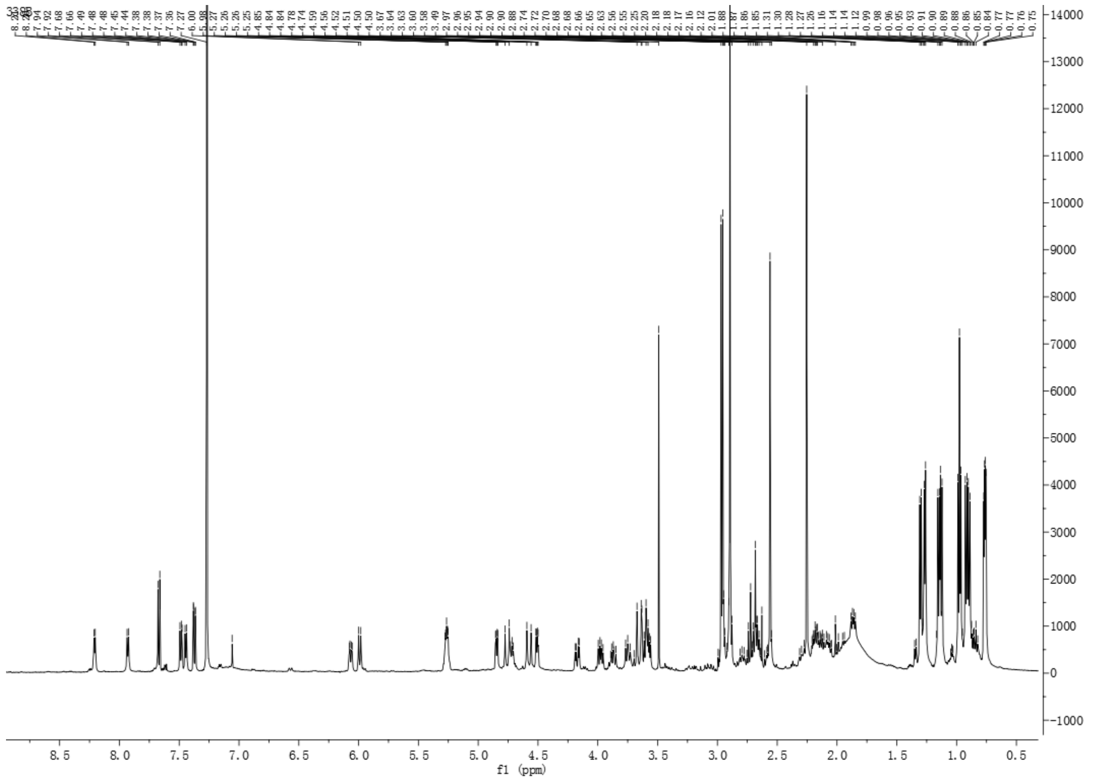

Figure S65. ^{1}H NMR spectrum of actinomycin A1 (**3**, in $CDCl_3$-*d*).

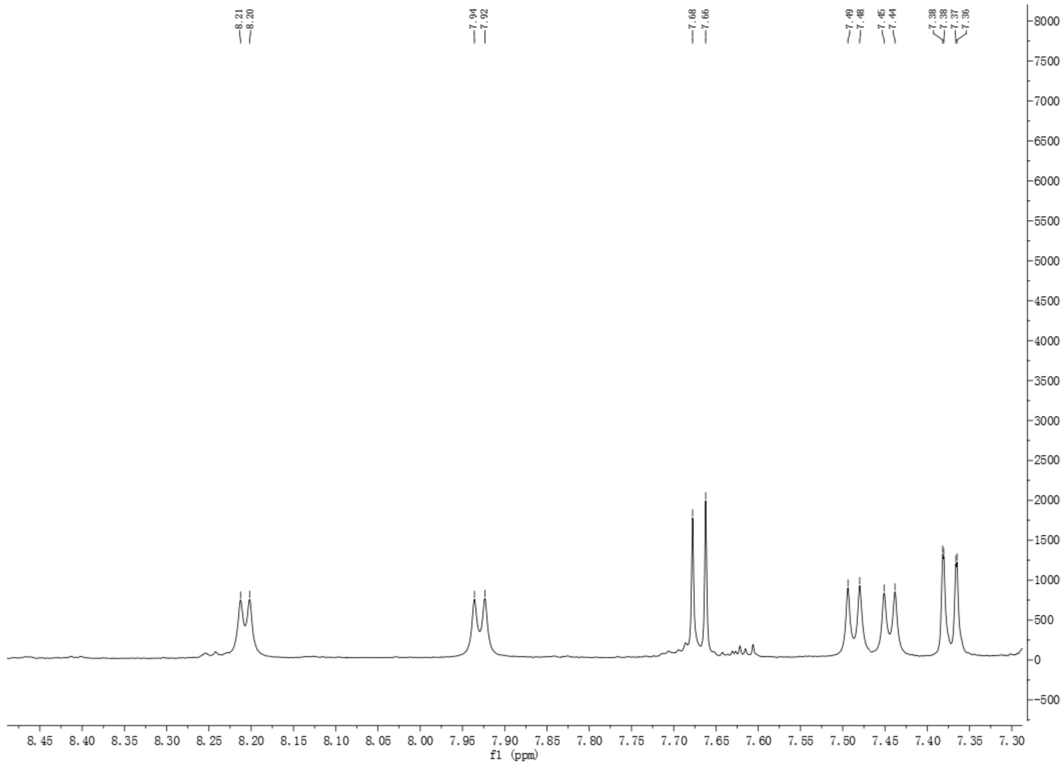

Figure S66. ^{1}H NMR spectrum of actinomycin A1 (**3**, in $CDCl_3$-*d*).

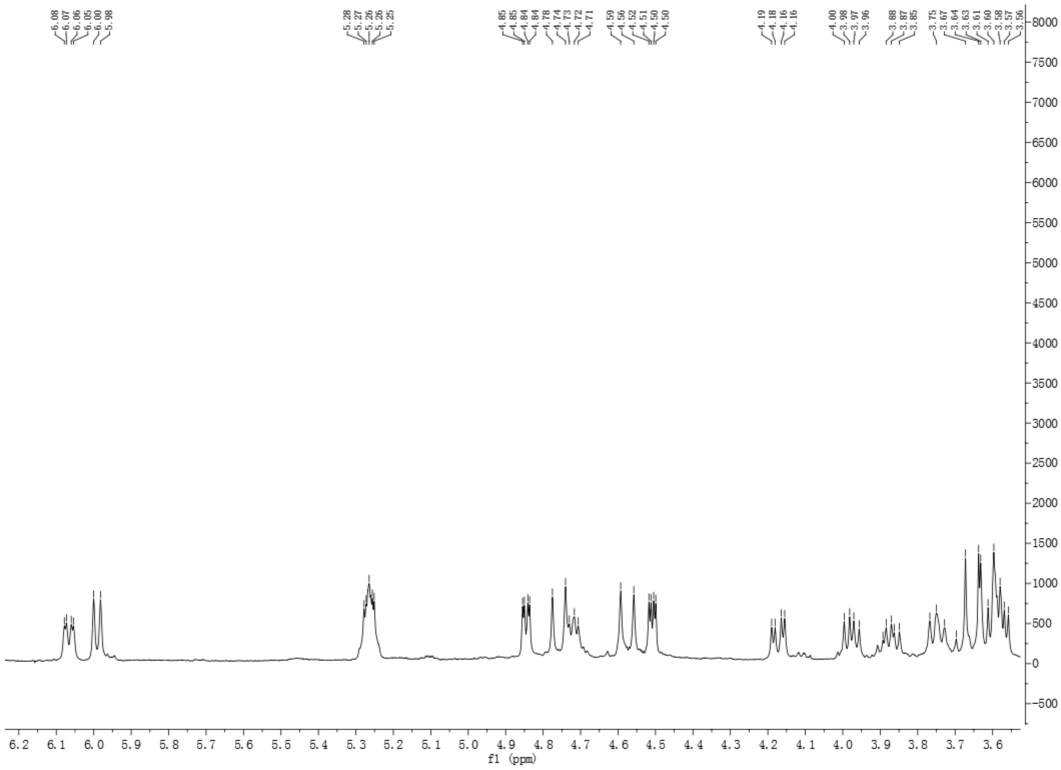

Figure S67. ^{1}H NMR spectrum of actinomycin A1 (**3**, in $CDCl_3$-*d*).

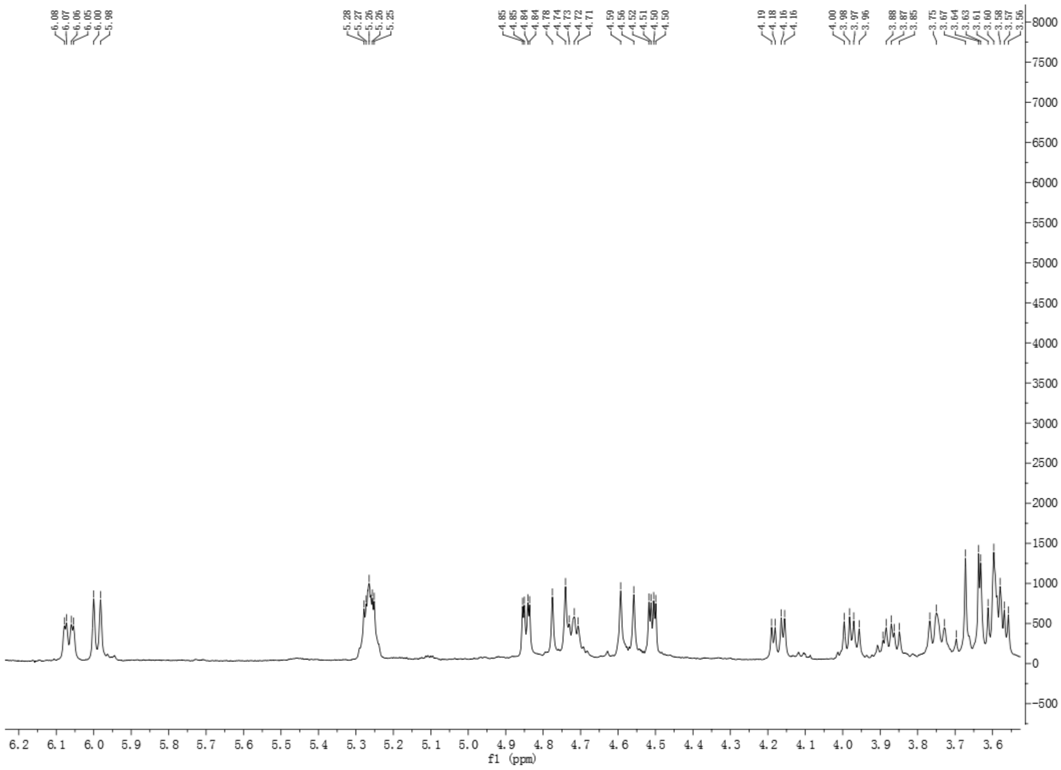

Figure S68. ^{1}H NMR spectrum of actinomycin A1 (**3**, in $CDCl_3$-*d*).

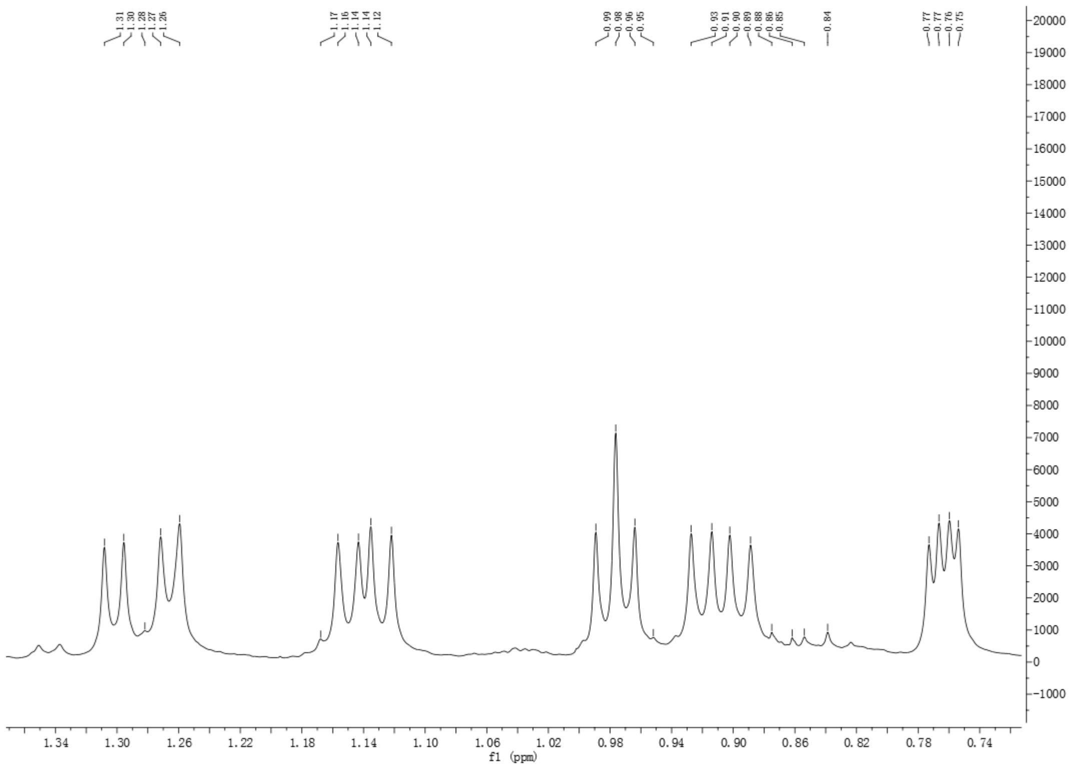

Figure S69. ^{1}H NMR spectrum of actinomycin A1 (**3**, in $CDCl_3$-*d*).

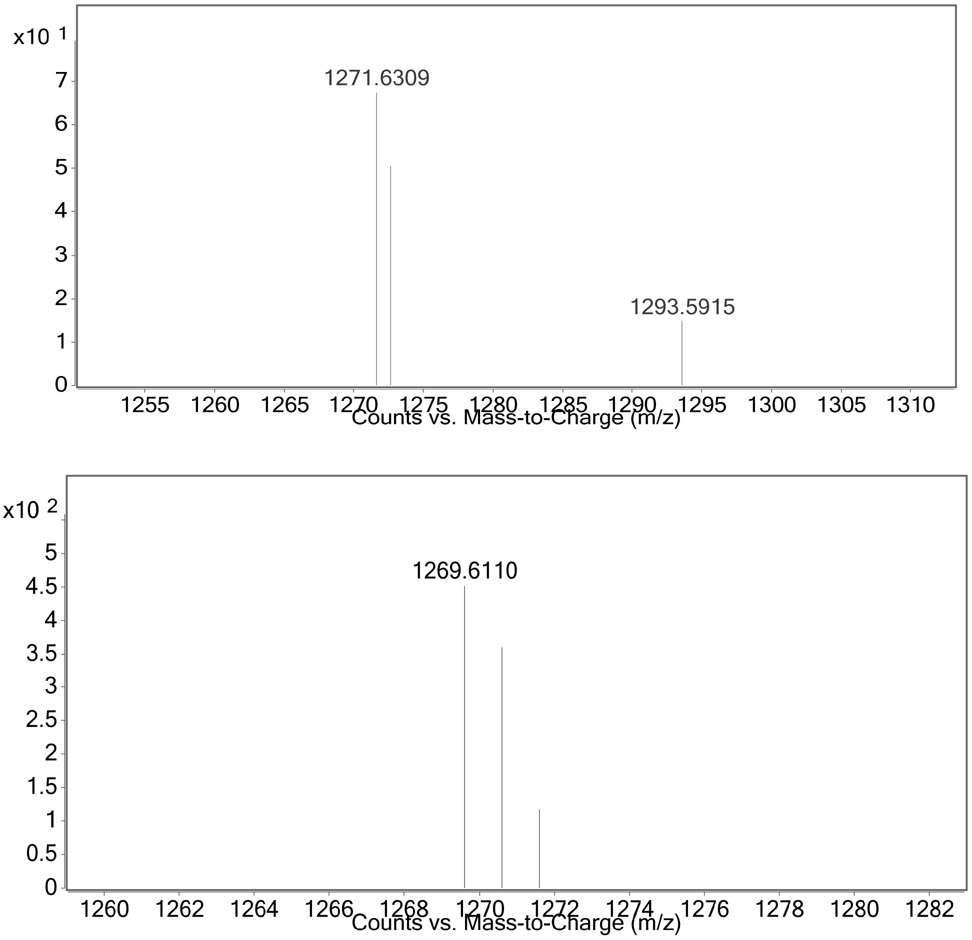

Figure S70. HRESIMS of actinomycin A1 (**3**, in $CDCl_3$-*d*).

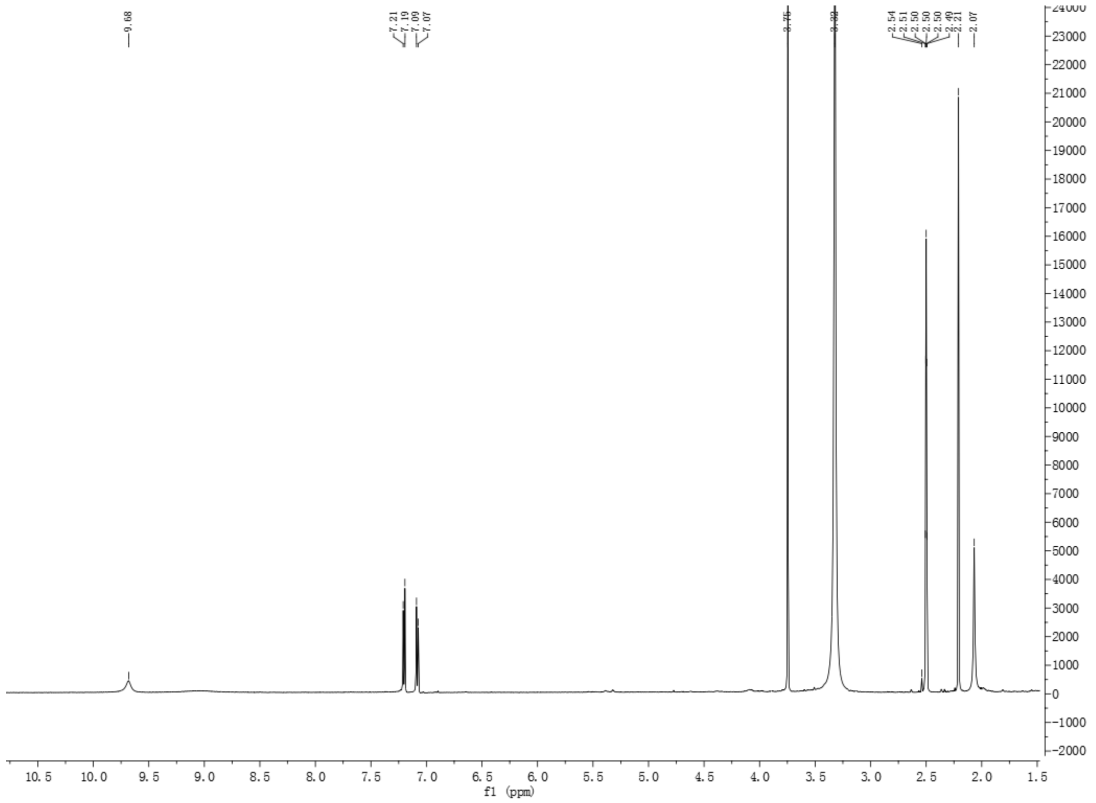

Figure S71. ^{1}H NMR spectrum of compound **4** (in DMSO-*d*6).

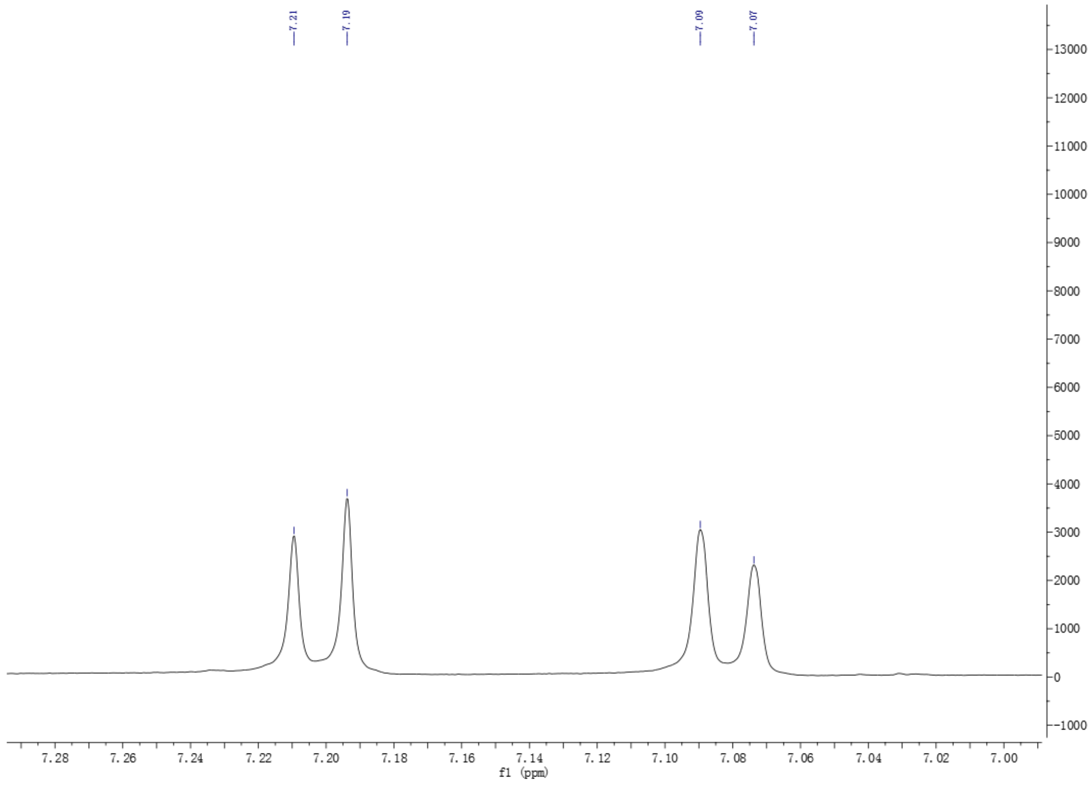

Figure S72. ^{1}H NMR spectrum of compound **4** (in DMSO-*d*6).

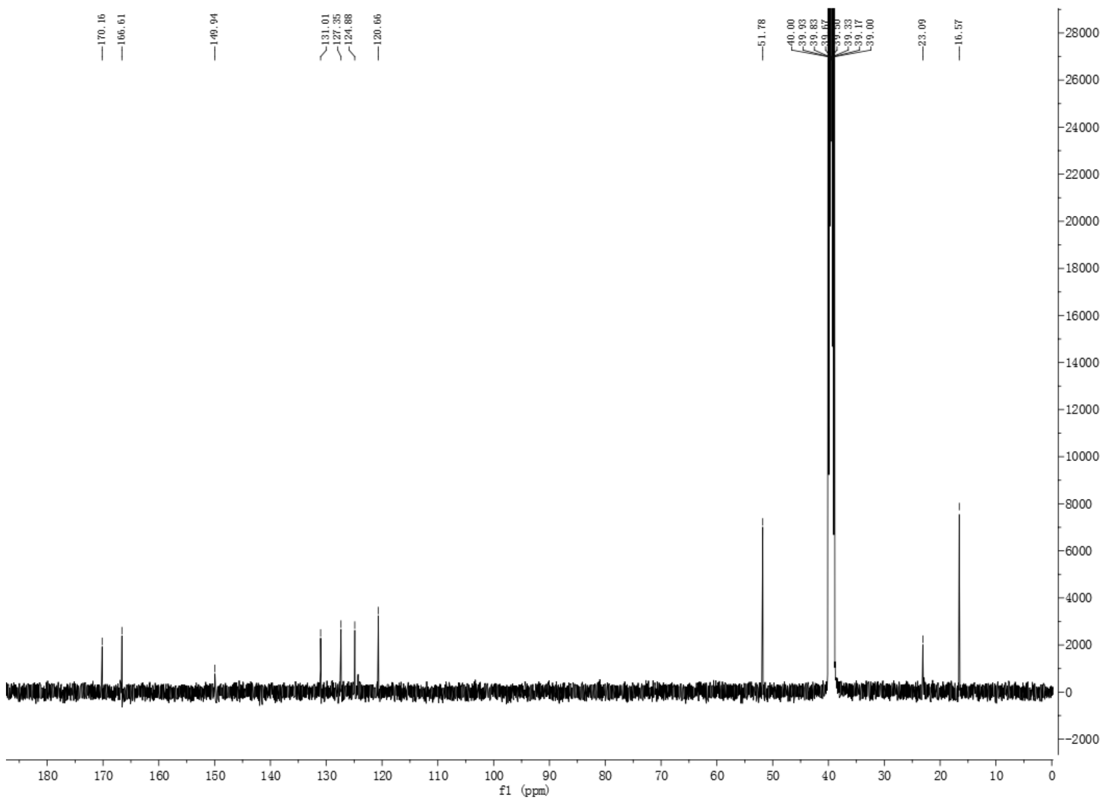

Figure S73. ^{13}C NMR spectrum of compound **4** (in DMSO-*d*6).

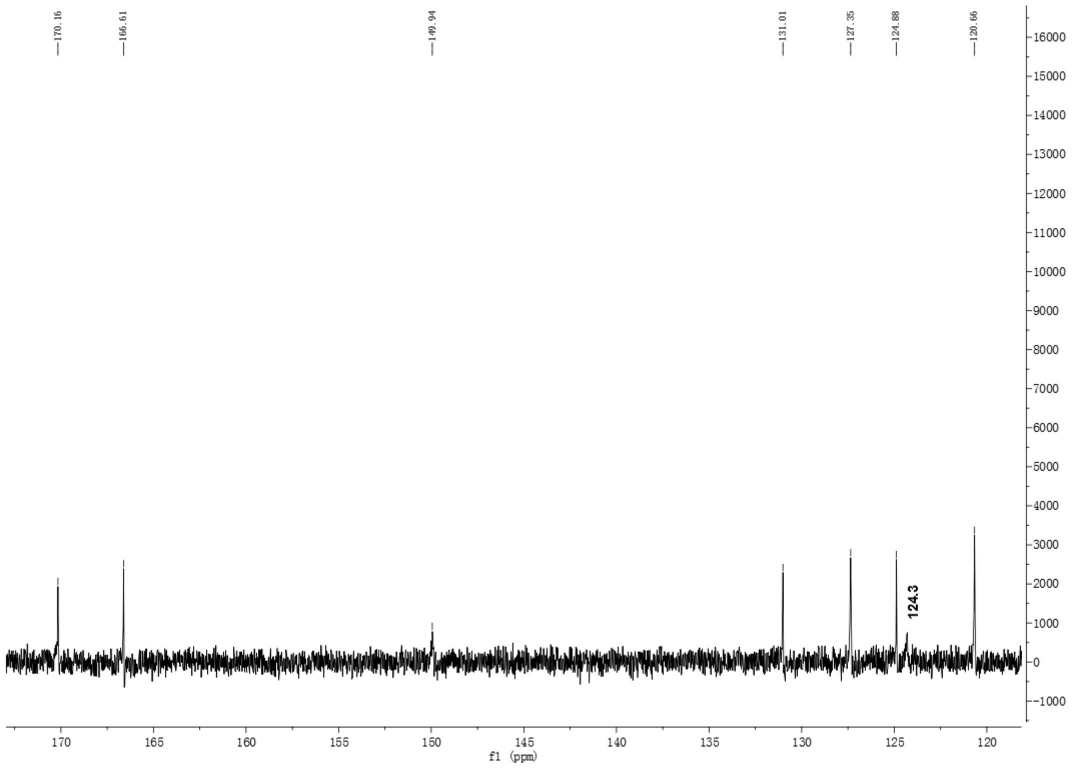

Figure S74. ^{13}C NMR spectrum of compound **4** (in DMSO-*d*6).

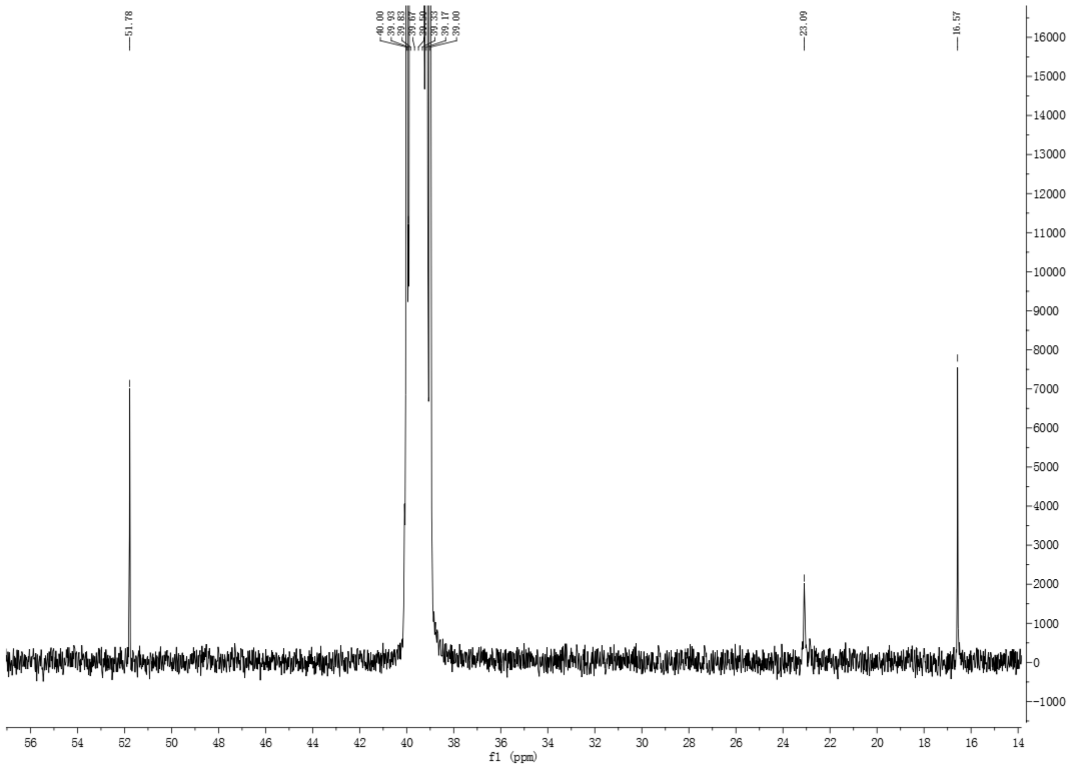

Figure S75. ^{13}C NMR spectrum of compound **4** (in DMSO-*d*6).

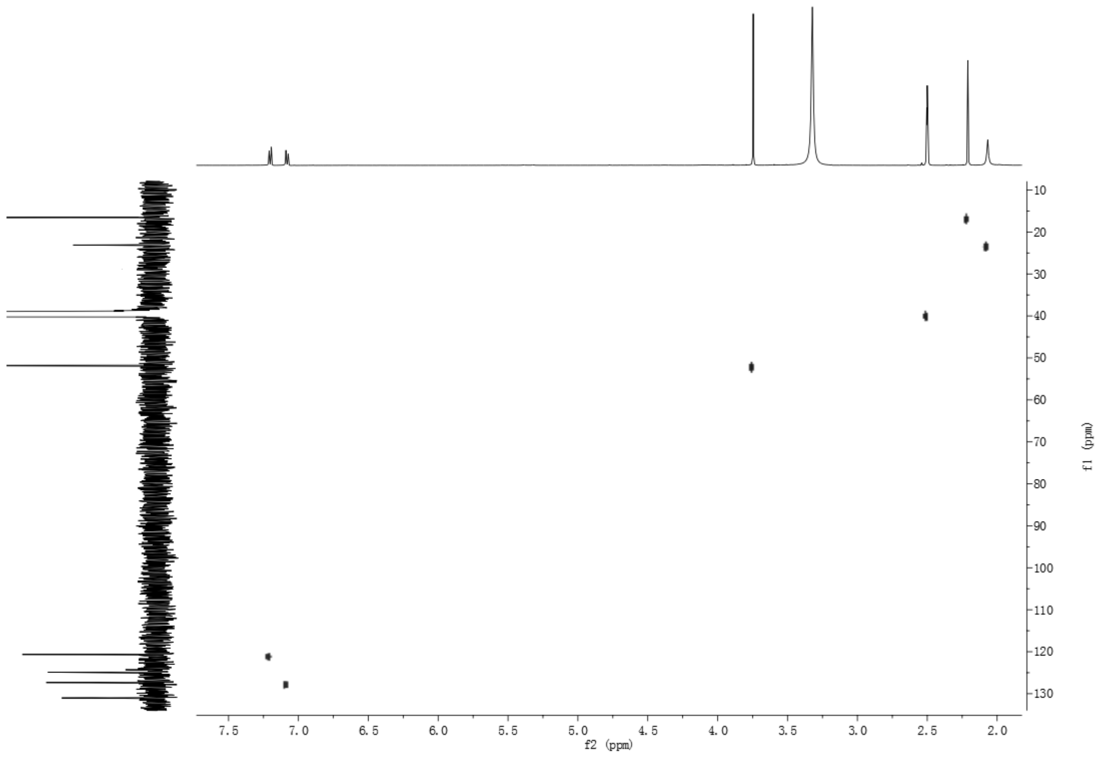

Figure S76. HSQC spectrum of compound **4** (in DMSO-*d*6).

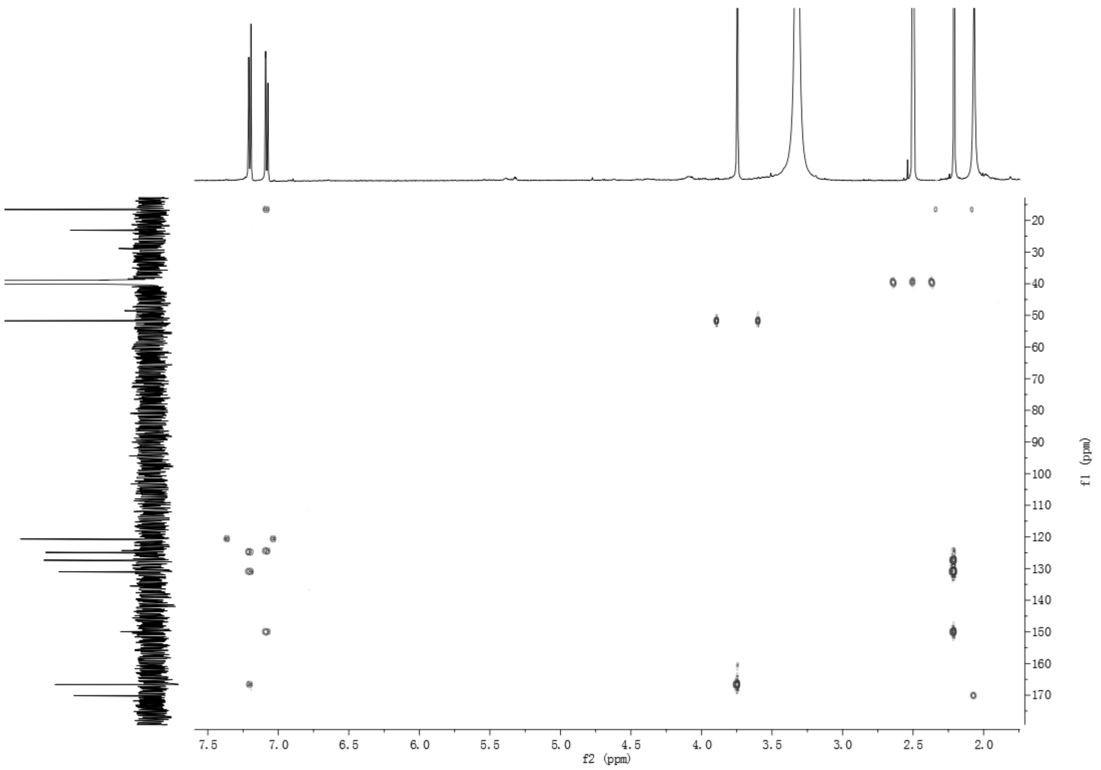

Figure S77. HMBC spectrum of compound **4** (in DMSO-*d*6).

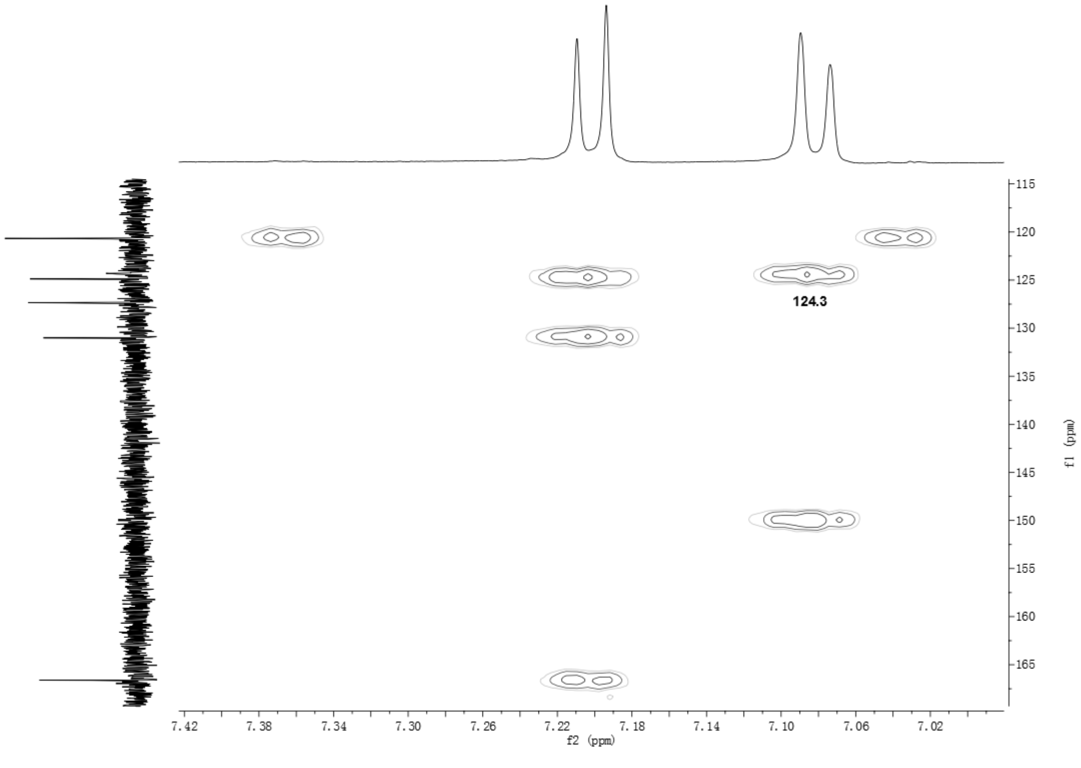

Figure S78. HMBC spectrum of compound **4** (in DMSO-*d*6).

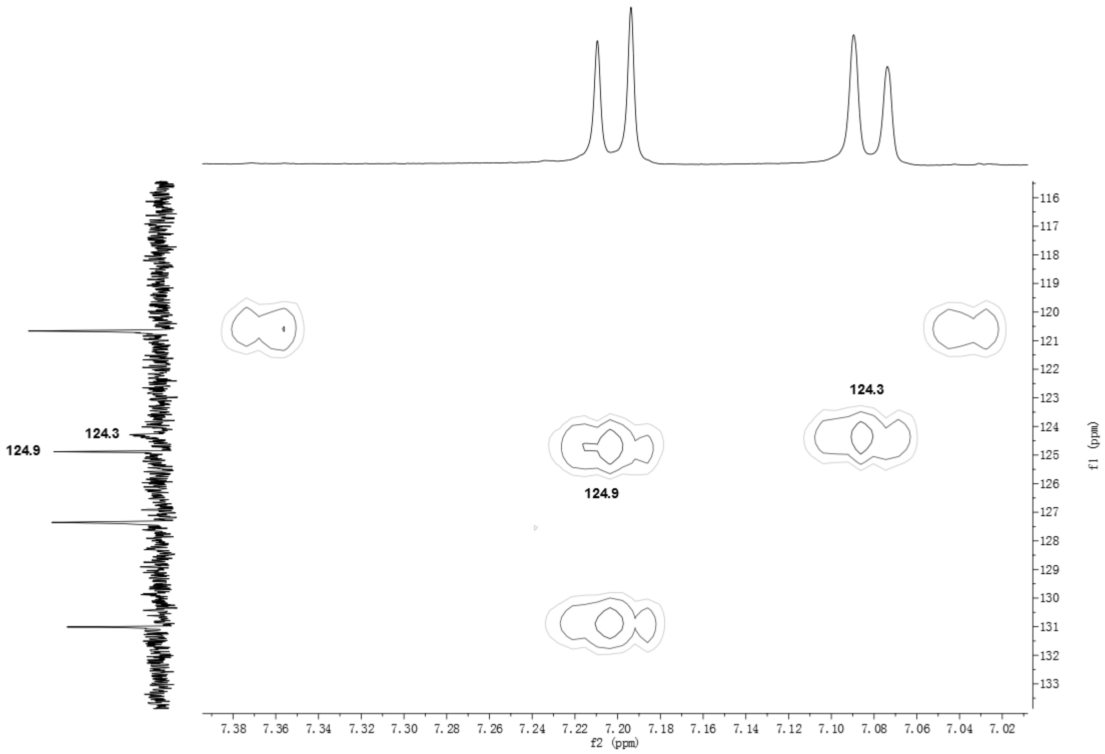

Figure S79. HMBC spectrum of compound **4** (in DMSO-*d*6).

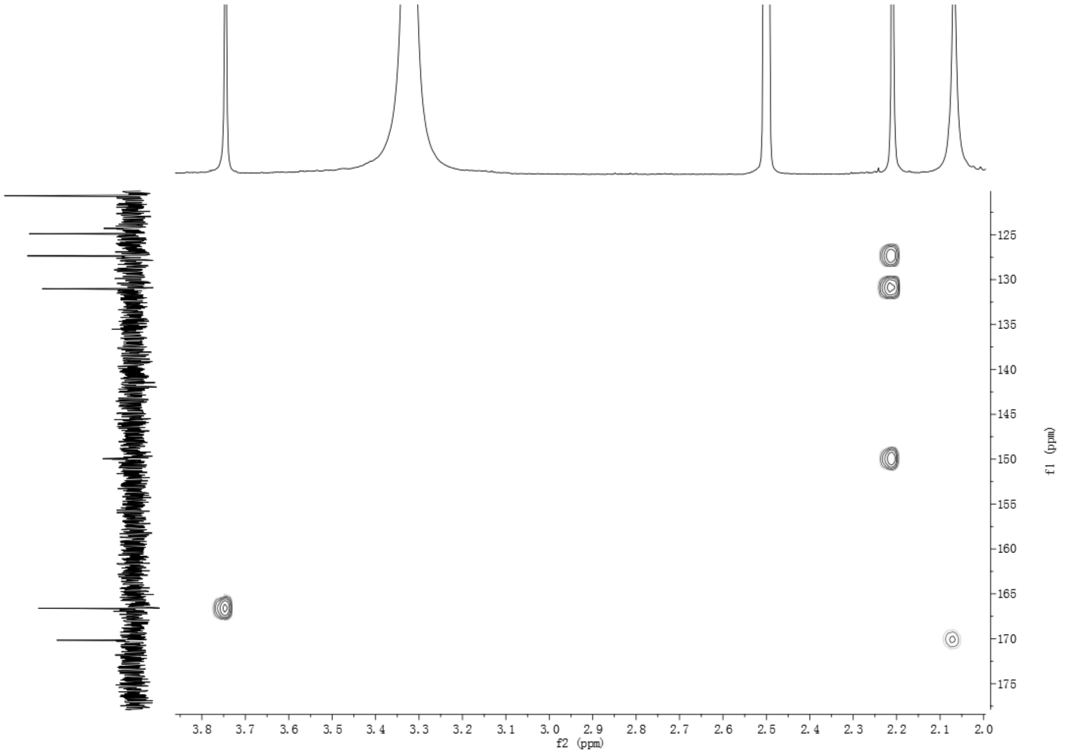

Figure S80. HMBC spectrum of compound **4** (in DMSO-*d*6).

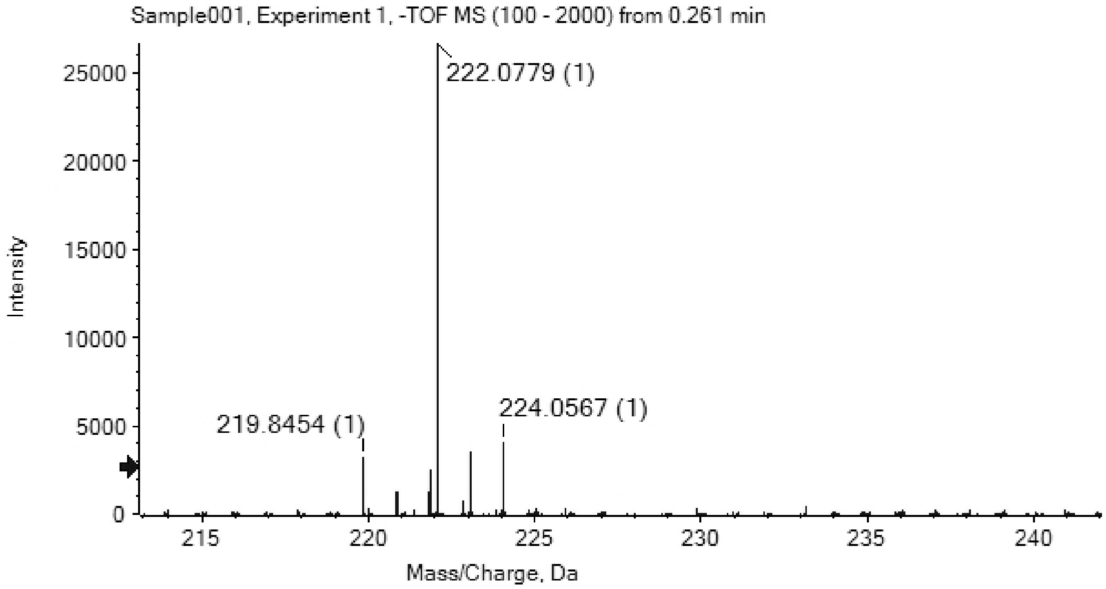

Figure S81. HRESIMS of compound **4**.

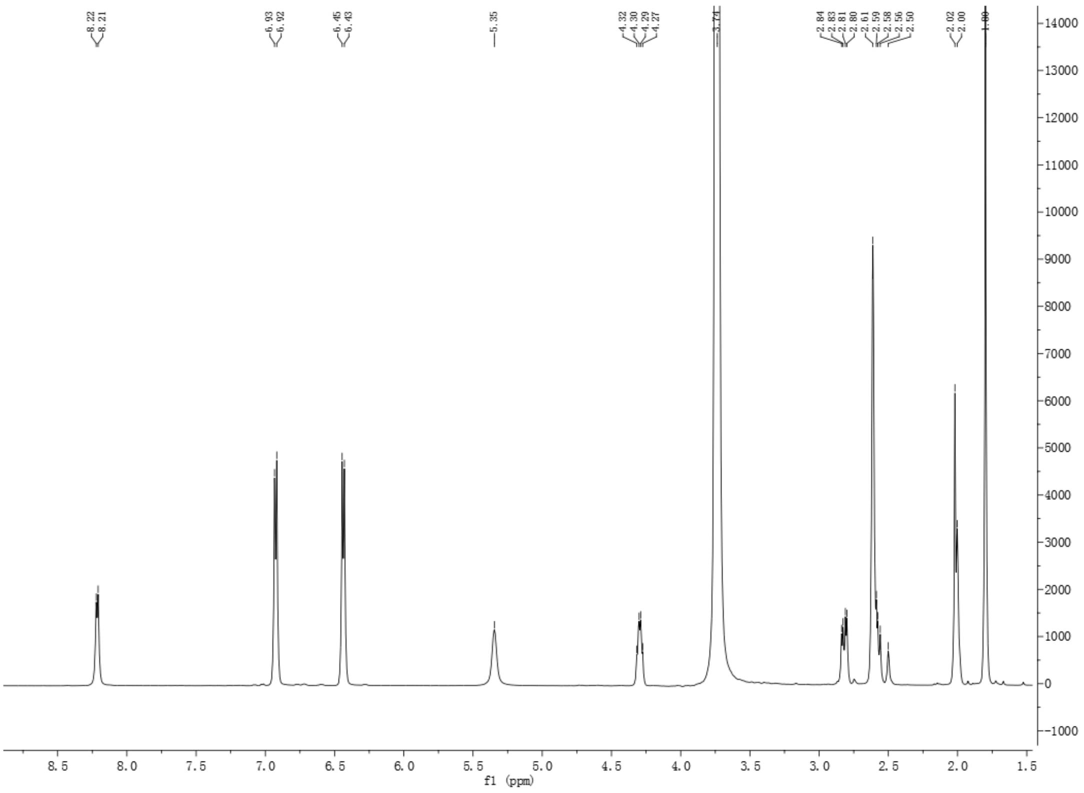

Figure S82. ^{1}H NMR spectrum of compound **5** (in DMSO-*d*6).

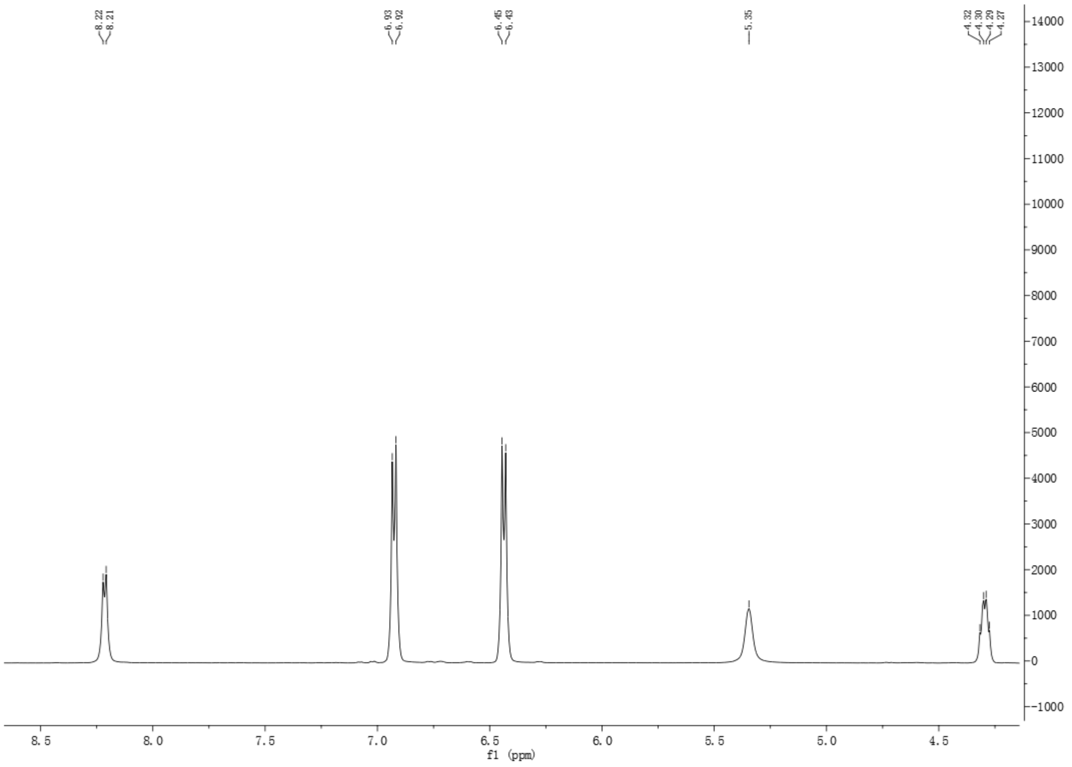

Figure S83. ^{1}H NMR spectrum of compound **5** (in DMSO-*d*6).

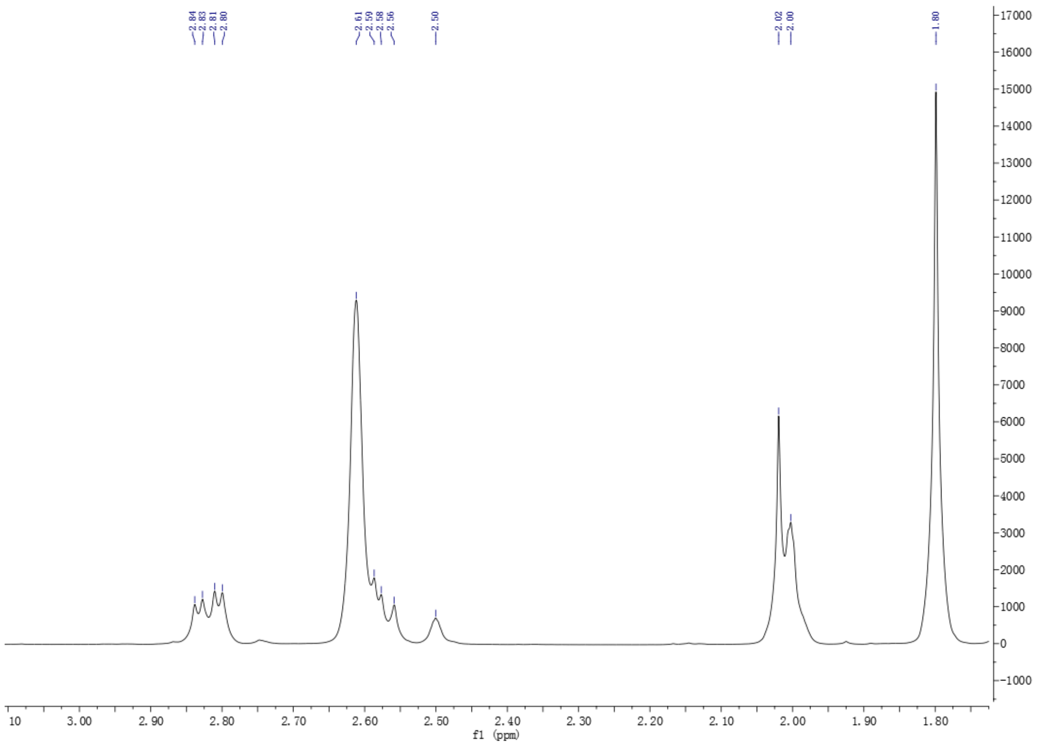

Figure S84. ^{1}H NMR spectrum of compound **5** (in DMSO-*d*6).

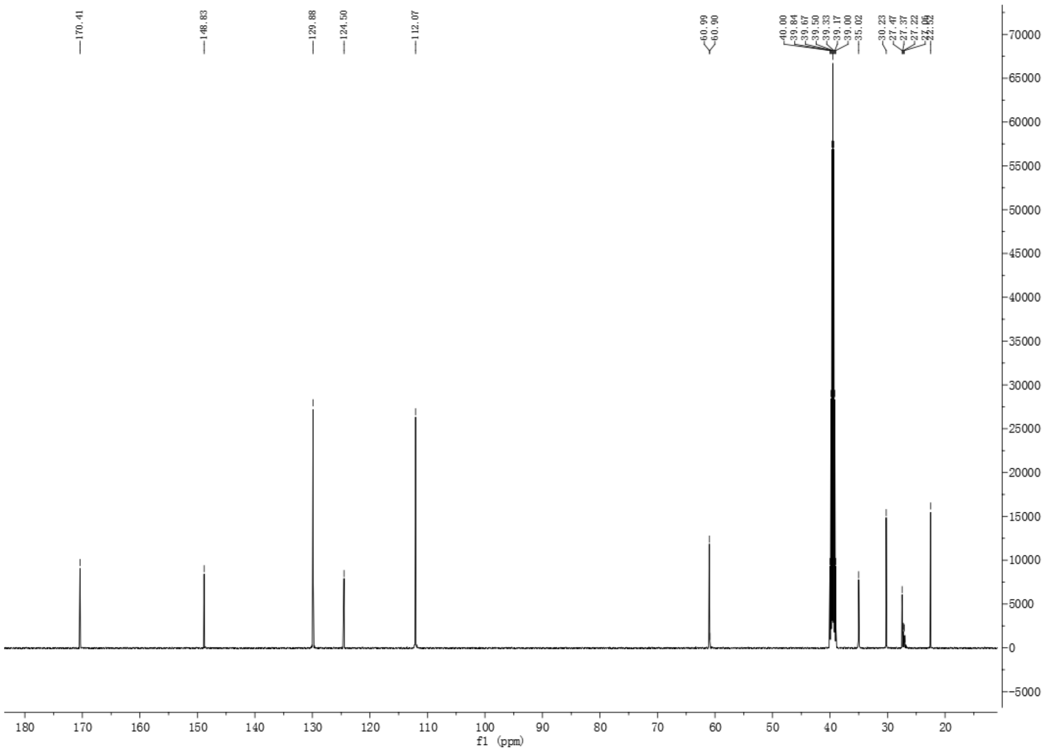

Figure S85. ^{13}C NMR spectrum of compound **5** (in DMSO-*d*6).

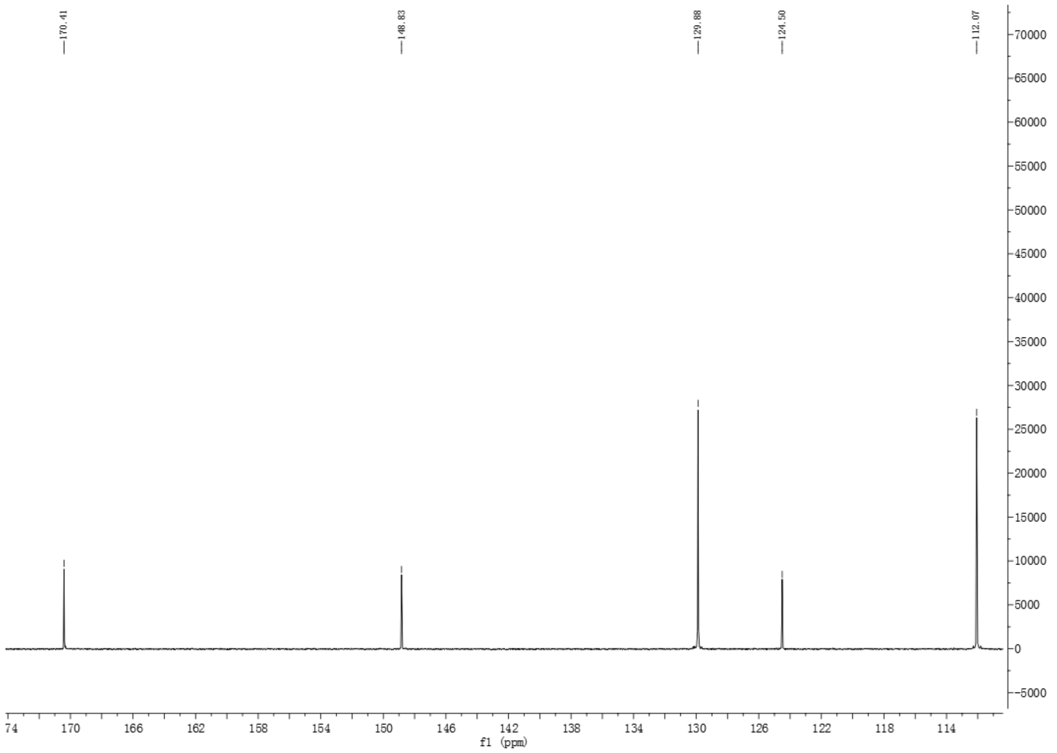

Figure S86. ^{13}C NMR spectrum of compound **5** (in DMSO-*d*6).

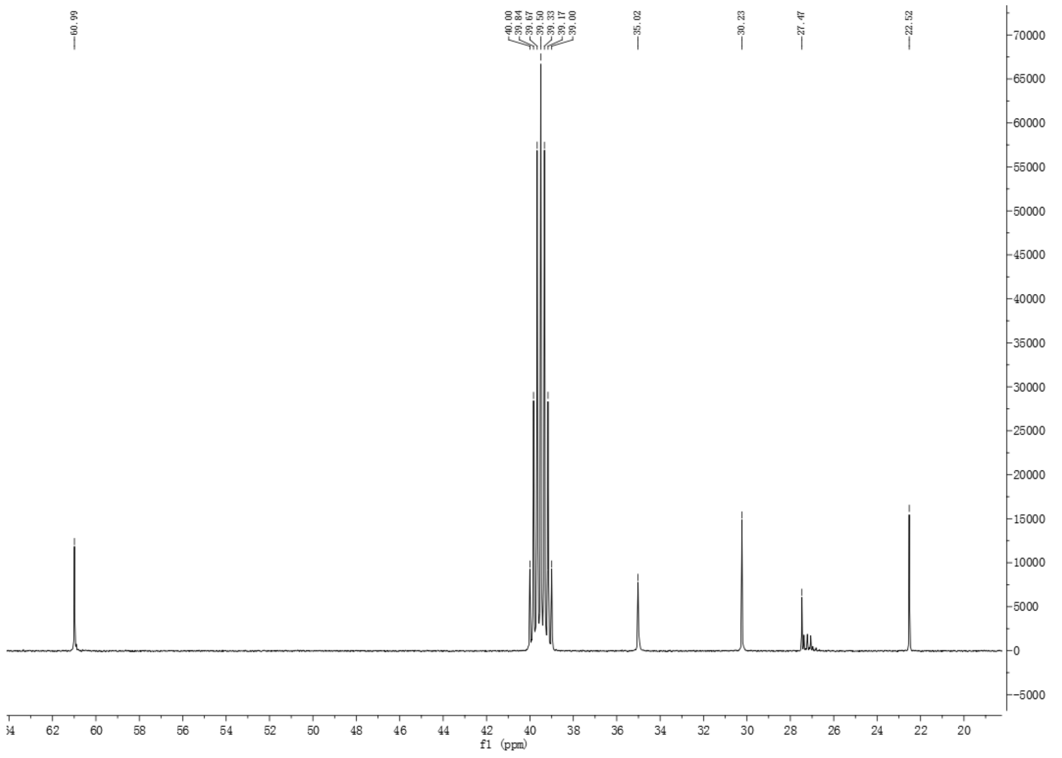

Figure S87. ^{13}C NMR spectrum of compound **5** (in DMSO-*d*6).

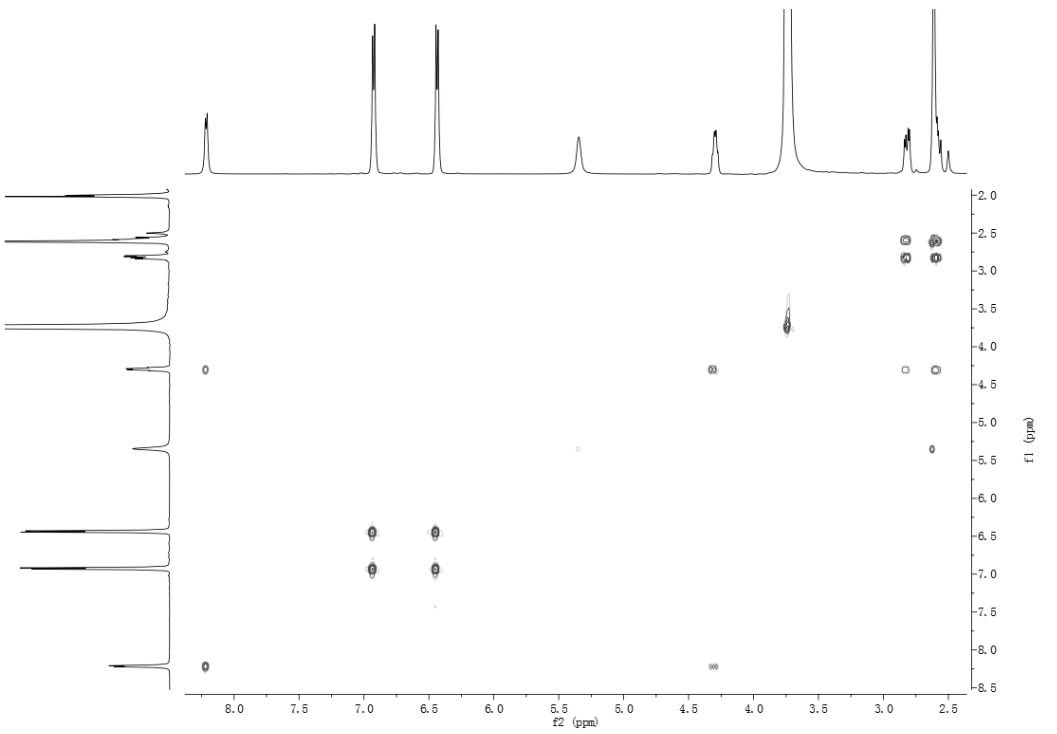

Figure S88. ^{1}H–^{1}H COSY spectrum of compound **5** (in DMSO-*d*6).

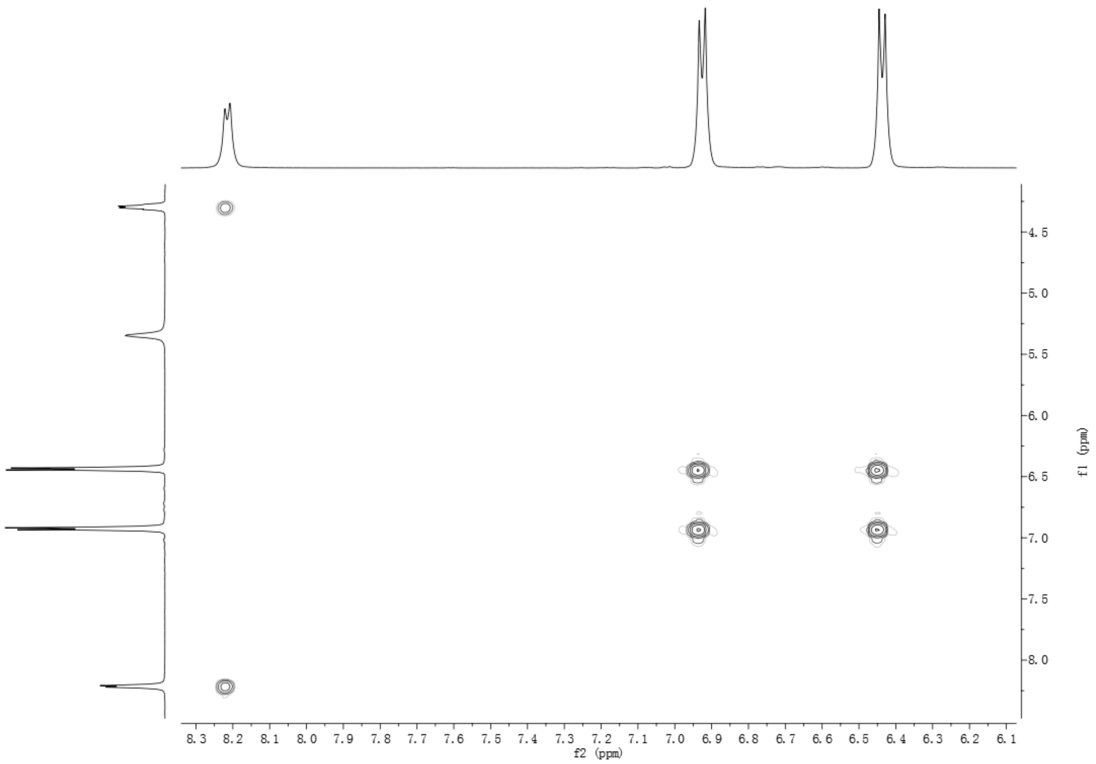

Figure S89. ^1H–^1H COSY spectrum of compound **5** (in DMSO-*d6*).

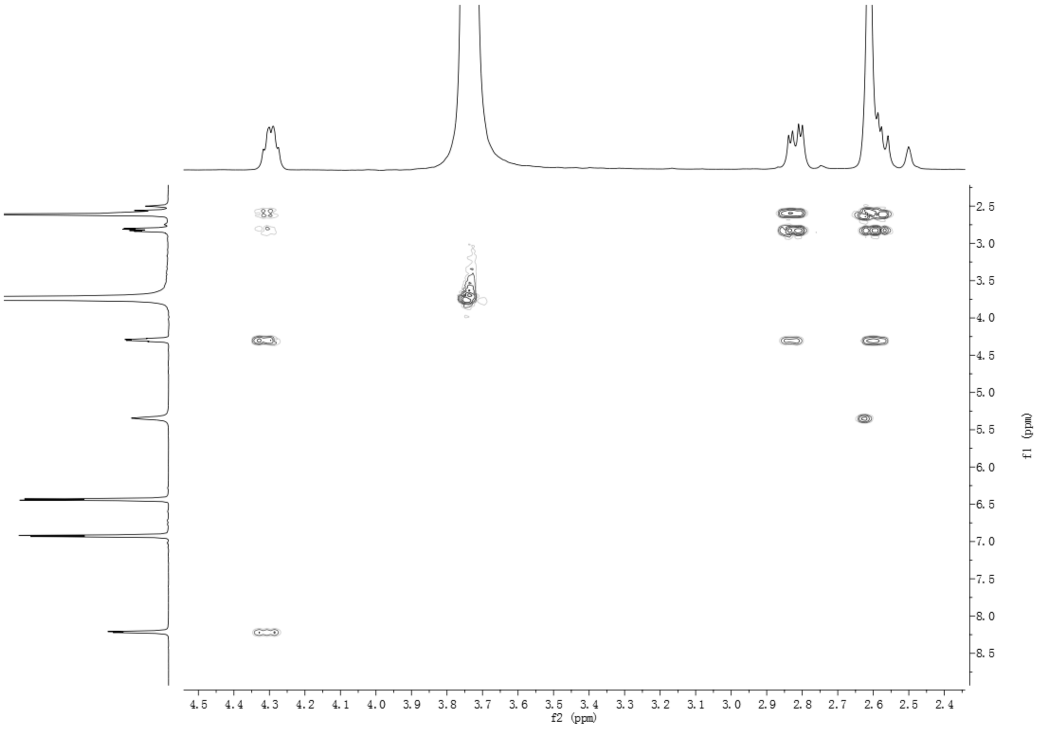

Figure S90. ^1H–^1H COSY spectrum of compound **5** (in DMSO-*d6*).

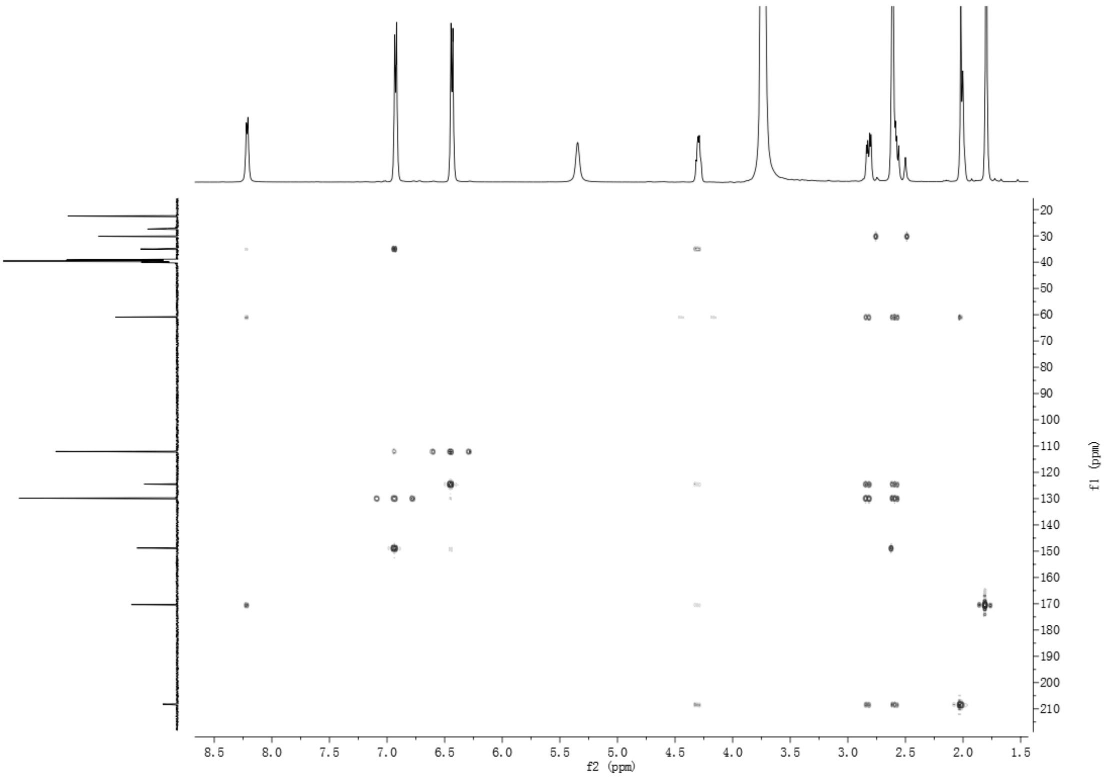

Figure S91. HMBC spectrum of compound **5** (in DMSO-*d*6).

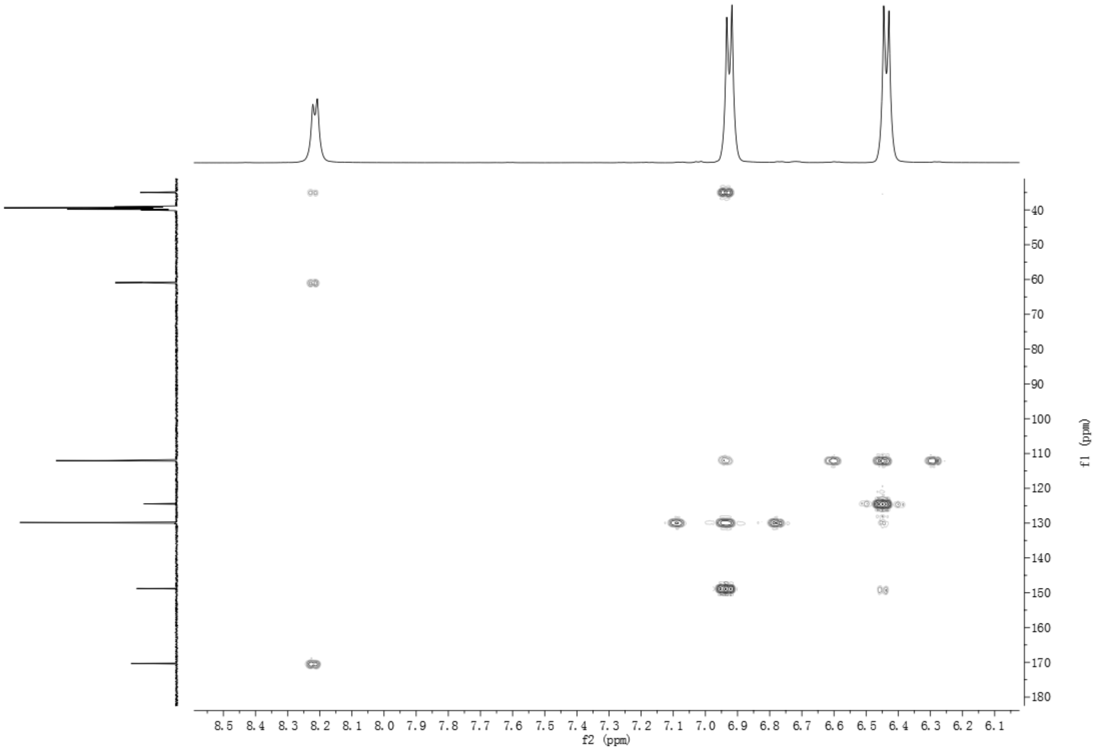

Figure S92. HMBC spectrum of compound **5** (in DMSO-*d*6).

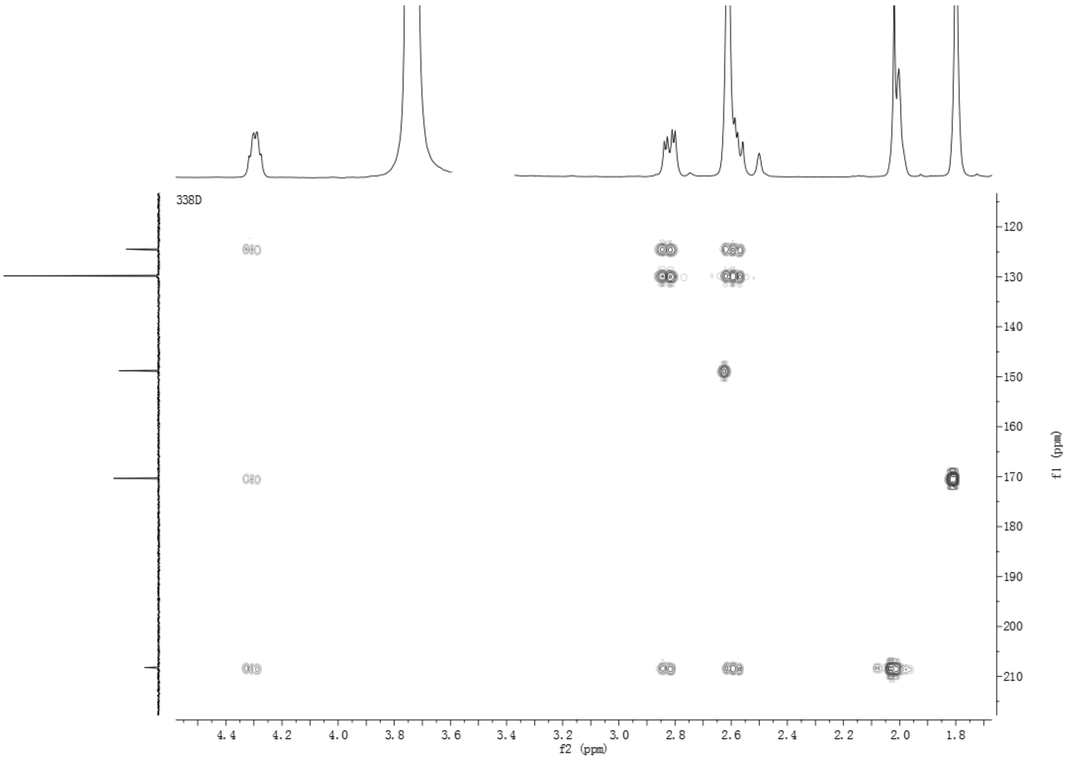

Figure S93. HMBC spectrum of compound **5** (in DMSO-*d*6).

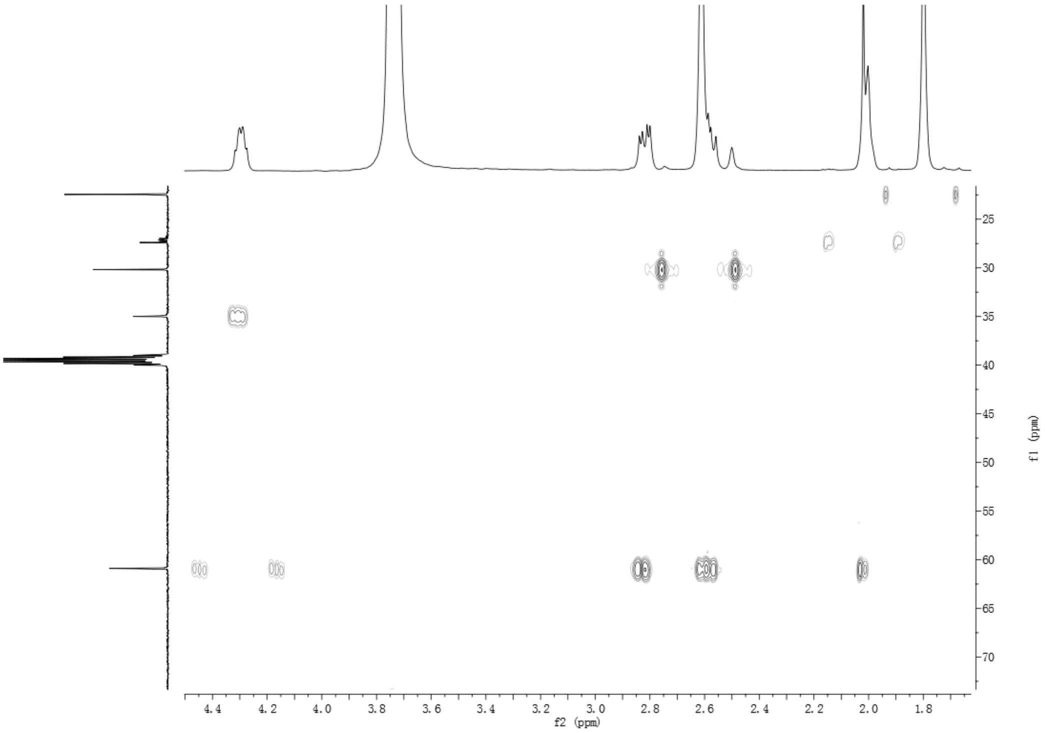

Figure S94. HMBC spectrum of compound **5** (in DMSO-*d*6).

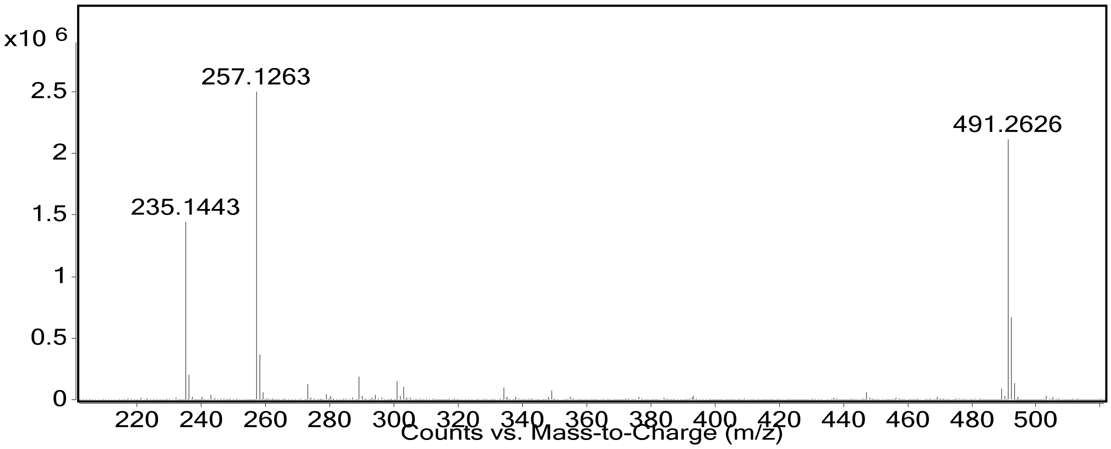

Figure S95. HRESIMS of compound **5** (in DMSO-*d6*).
